# Discovery of 2-Phenylquinoline-4-Carboxylic Acid Derivatives as Novel Histone Deacetylase Inhibitors

**DOI:** 10.3389/fchem.2022.937225

**Published:** 2022-07-14

**Authors:** Qian Hui, Lihui Zhang, Jinhong Feng, Lei Zhang

**Affiliations:** ^1^ Department of Medicinal Chemistry, School of Pharmacy, Weifang Medical University, Weifang, China; ^2^ School of Stomatology, Weifang Medical University, Weifang, China; ^3^ Shandong Analysis and Test Center, Qilu University of Technology (Shandong Academy of Sciences), Jinan, China

**Keywords:** histone deacetylase, inhibitor, anticancer, selectivity, cell cycle, apoptosis

## Abstract

Inhibition of histone deacetylases (HDACs) has been extensively studied in the development of anticancer drugs. In the discovery of potent HDAC inhibitors with novel structures, the 2-substituted phenylquinoline-4-carboxylic acid group was introduced to the cap moiety of HDAC inhibitors. In total, 30 compounds were synthesized with hydroxamic acid or hydrazide zinc-binding groups. In the enzyme inhibitory test, active compound **D28** and its analog **D29** exhibited significant HDAC3 selectivity against HDAC1, 2, 3, and 6. However, compared with **D28**, the hydrazide-bearing compounds (**D29** and **D30**) with remarkably improved enzyme inhibitory activities did not exhibit significant antiproliferative potency in the *in vitro* anticancer study. Further K562 cell-based mechanistic results revealed that induction of G2/M cell cycle arrest and promotion of apoptosis make important contributions to the anticancer effects of molecule **D28**. Collectively, an HDAC3 selective inhibitor (**D28**) with potent *in vitro* anticancer activity was developed as a lead compound for the treatment of cancer.

## Introduction

Histone deacetylases (HDACs) are a group of enzymes that are responsible for the removal of acetyl group from ɛ-N-acetyl-lysine amino groups of histone proteins (Bernstein et al., 2000; de Ruijter et al., 2003). Regulated by HDACs and histone acetyltransferases (HATs), the balance of acetylation levels significantly contributes to the modulation of cellular functions and activities. In humans, a total of 18 HDAC isoforms have been identified and classified into four classes (Foglietti et al., 2006; Zhang et al., 2015). Among them, class I HDACs (HDACs 1, 2, 3, and 8), class II HDACs (HDACs 4, 5, 6, 7, 9, and 10), and class IV HDAC (HDAC 11) are zinc-dependent enzymes. In contrast, class III (sirtuins, Sirt1-7) HDACs are a family of NAD^+^-dependent enzymes which have remarkable structural differences from other classes of HDACs.

Overexpression and aberrant recruitment of HDACs are closely related to tumorigenesis and cancer aggravation (Marks et al., 2001; Zhang et al., 2019). Therefore, inhibition of HDACs has been extensively studied as a potential therapeutic target in the development of anticancer drugs. Vorinostat (SAHA) (Marks, 2007), romidepsin (FK-228) (Ueda et al., 1994), belinostat (PXD101) (Yang et al., 2010), and panobinostat (LBH589) (Neri et al., 2012) have been approved by United States FDA for the treatment of cutaneous T-cell lymphoma (CTCL), peripheral T-cell lymphoma (PTCL), and multiple myeloma, respectively. An increasing number of anticancer agents targeting HDACs are currently being developed in various stages.

The pharmacophores of a typical HDAC inhibitor consists of a zinc-binding group (ZBG), a linker, and a cap region. According to ZBGs and chemical structures, HDAC inhibitors can be categorized into four types, including hydroxamic acids, benzamides, cyclic peptides, and aliphatic carboxylic acids (Zhang et al., 2010). Among the available ZBGs, the hydroxamic acid group had been widely utilized for the design of HDAC inhibitors. Cap moiety responsible for binding to the hydrophobic region at the opening of the HDAC active site, can also block other substrates from entering the HDAC catalytic pocket. The linker located in the channel of the HDAC active site was used to connect the ZBG and cap structures.

**FIGURE 1:**
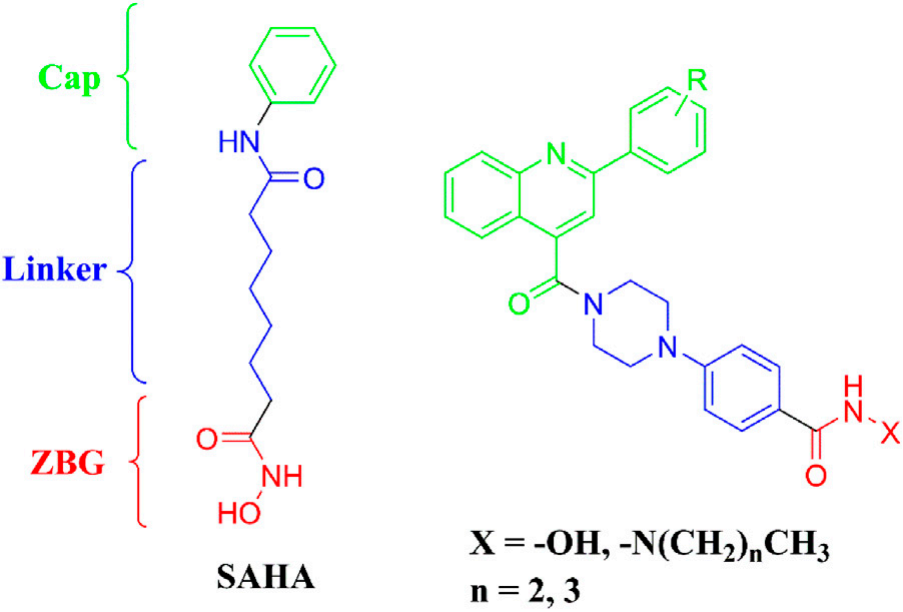
Design of 2-substituted phenylquinoline-4-carboxylic acid bearing HDAC inhibitors.

Cap group is an important feature in the design of novel HDAC inhibitors. Different cap structures play critical roles in the structural diversity of HDAC inhibitors. In the present study, the 2-substituted phenylquinoline-4-carboxylic acid group was introduced to the cap region for potent new HDAC inhibitors (Figure 1). The structure with multiple aromatic rings was designed to form strong hydrophobic interactions with residues in the opening of HDAC active site. The hydroxamic acid group was incorporated as the most commonly used ZBG and has exhibited advantages of both high HDAC (or zinc ion) affinity and potent antiproliferative activities (Zhang et al., 2018). Hydrazides as ZBGs have been revealed to have inhibitory selectivity of class I HDACs, especially in the inhibition of HDAC3 (Jiang et al., 2022). Therefore, hydrazides and the frequently used hydroxamic acid were utilized as ZBGs in the design of target compounds. The phenylpiperazine group was used as the linker to connect the substituted quinoline-4-carboxylic acid and ZBGs. The designed molecules were synthesized and evaluated in the *in vitro* enzyme inhibitory assay, antiproliferative study, cell cycle, and apoptosis tests.

## Chemistry

**SCHEME 1:**
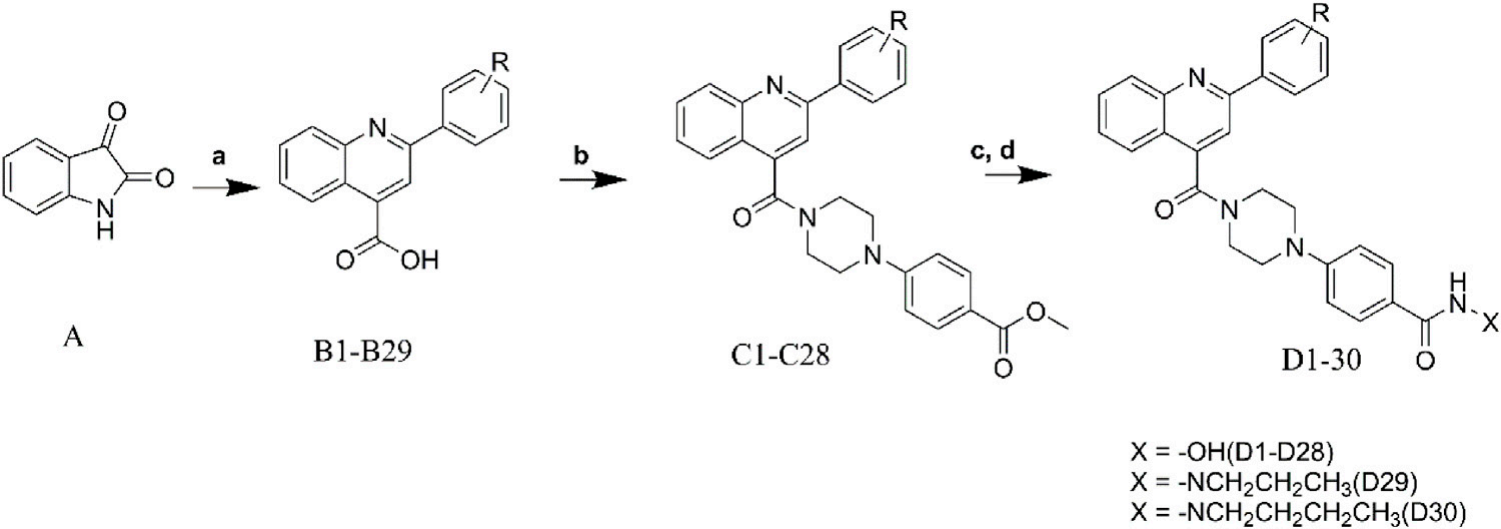
Synthesis of the designed compounds. Reagents and conditions: **(A)** substituted acetophenone, KOH, EtOH, 80°C; **(B)** TBTU, Et_3_N, DCM, 0°C; **(C)** NH_2_OK, CH_3_OH, rt; **(D)** NH_2_NH_2_.H_2_O, MeOH, reflux, 12–48 h; aliphatic aldehydes, NaBH_4_, rt, 4 h.

Target compounds were synthesized as described in Scheme 1. The commercially available isatin (**A**) was used as the starting material for the compound synthesis. Intermediates **B1-B29** were obtained by the coupling of isatin with substituted acetophenone through the Pfitzinger reaction (Swain et al., 2022). After that, key intermediates **C1-C28** were derived by the condensation of the intermediates **B1-B29** with 4-(piperazine-1-yl) methyl benzoate (Yamada et al., 2017). Target molecules **D1-D28** were synthesized by treatment of intermediates **C1-C28** with NH_2_OK in methanol. As to compounds **D29-D30**, hydrazides were introduced by hydrazinolysis of **C28**, and the subsequent condensation and reduction reactions.

## Results and Discussion

### Enzymatic Inhibition Assay

**FIGURE 2:**
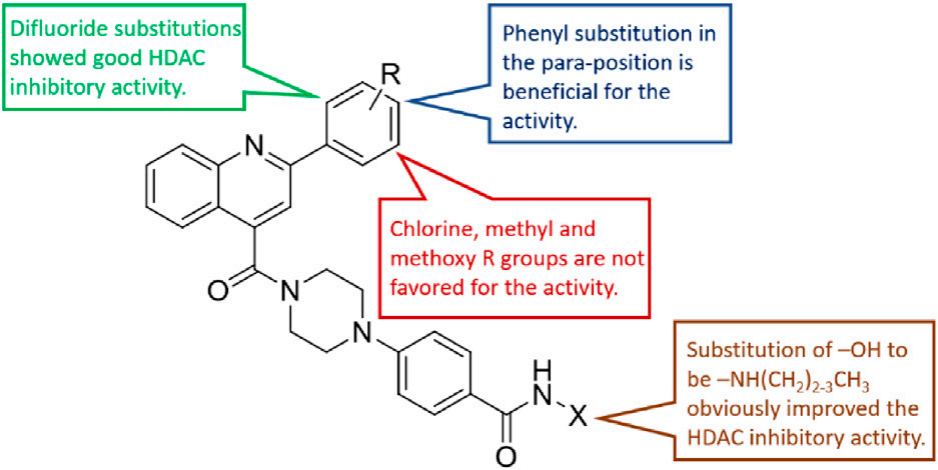
SAR analysis of the derived compounds based on the HDAC enzyme inhibitory results.

The HDAC enzyme inhibitory activities of synthesized compounds were evaluated against Hela nucleus extract containing a mixture of HDAC isoforms. Percentage inhibitory rate (PIR) was calculated to determine the activity of tested compounds (Table 1). The compounds **(D1-28)** were firstly synthesized for the activity screening. The results showed that compounds **D11**, **D12**, **D23**, **D24,** and **D28** exhibited good HDAC inhibitory activity with PIR of 63.49%, 74.91%, 66.16%, and 68.00%, respectively, at a concentration of 2 μM. Compared with the unsubstituted **D1**, the difluoride-substitution and phenyl substitution can be conducive to the inhibitory activity. However, the chlorine-substituted compounds, such as **D3** (PIR of 49.43%), **D8** (PIR of 51.81%), and **D14** (PIR of 49.74%), showed decreased activity compared with **D1**. Similarly, the methyl substitution and methoxy substitution in the phenyl ring reduced the HDAC inhibitory potency. As illustrated in Figure 2, the SAR analysis results provided evidence for further structural modification of current compounds. To avoid excessive molecular weight and increase the HDAC inhibitory activity, the R group with small size and molecular weight could be reserved for further structural modification.

**TABLE 1:** Structure and inhibitory activity of target compounds at 2 µM against HDACs and K562 cells.

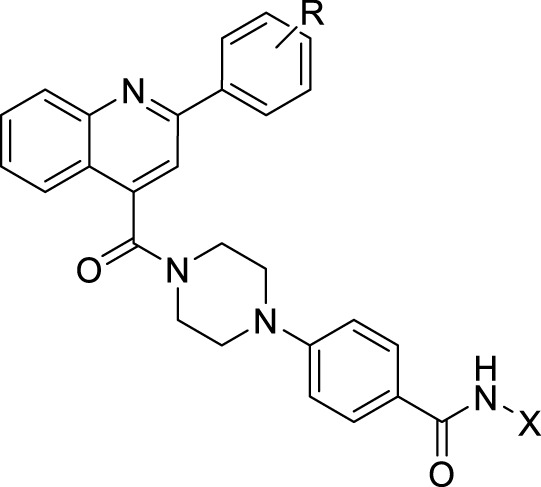				
Compound	R	X	HDACs^a^	K562^a^
D1	-H	-OH	57.96 ± 0.43	46.58 ± 0.35
D2	4-Br	-OH	59.50 ± 0.35	48.84 ± 1.32
D3	4-Cl	-OH	49.43 ± 3.73	46.38 ± 0.53
D4	4-F	-OH	47.79 ± 1.34	32.43 ± 0.20
D5	4-S-CH_3_	-OH	50.93 ± 0.93	47.39 ± 1.11
D6	4-OCH_3_	-OH	52.31 ± 3.09	51.55 ± 0.25
D7	4-CF_3_	-OH	52.60 ± 2.37	40.33 ± 1.20
D8	3-Cl	-OH	51.81 ± 4.07	42.86 ± 1.02
D9	3-CH_3_	-OH	49.33 ± 0.82	45.81 ± 0.33
D10	2-F	-OH	58.52 ± 0.64	40.88 ± 1.22
D11	3-OCH_3_	-OH	63.49 ± 4.61	46.09 ± 0.43
D12	3,5-2F	-OH	74.91 ± 0.35	51.81 ± 1.35
D13	2-OCH_3_	-OH	54.24 ± 0.18	43.81 ± 0.26
D14	2-Cl	-OH	49.74 ± 2.06	48.32 ± 1.02
D15	3-F	-OH	58.80 ± 2.95	26.24 ± 0.62
D16	4-CH_3_	-OH	49.04 ± 31.22	15.26 ± 0.33
D17	3-CH_3_-5-F	-OH	47.41 ± 1.03	41.91 ± 0.13
D18	4-N(CH_3_)_2_	-OH	48.37 ± 0.35	48.3 ± 0.31
D19	4-NHCO-phenyl	-OH	45.31 ± 0.68	59.19 ± 0.35
D20	4-NHCO-4-F-phenyl	-OH	48.46 ± 0.68	45.93 ± 1.34
D21	4-NHCO-3-Br-phenyl	-OH	50.26 ± 1.41	47.61 ± 0.92
D22	4-NHCO-2-F-phenyl	-OH	56.86 ± 1.79	65.07 ± 1.34
D23	4-NHCO-2-Cl-phenyl	-OH	62.91 ± 2.26	61.92 ± 0.42
D24	4-NHCO-2,4-2F-phenyl	-OH	66.16 ± 1.57	31.08 ± 0.54
D25	4-NHCO-2,5-2F-phenyl	-OH	54.74 ± 1.41	37.64 ± 0.13
D26	4-NHCO-3-F-phenyl	-OH	49.99 ± 0.32	43.07 ± 0.64
D27	3,6-2F	-OH	59.30 ± 0.74	63.79 ± 0.13
D28	4-Phenyl	-OH	68.00 ± 2.49	71.92 ± 2.32
D29	4-Phenyl	-NCH_2_CH_2_CH_3_	62.14 ± 2.11	62.36 ± 3.87
D30	4-Phenyl	-NCH_2_CH_2_CH_2_CH_3_	69.35 ± 3.44	65.77 ± 2.95
SAHA			50.12 ± 0.74	53.07 ± 0.25

a Illustrated as percentage inhibitory rate at concentration of 2.0 µM, and each value is the mean of three experiments.

Molecule **D28** exhibited significant activities in both enzymatic and K562 cell-based antiproliferative screening. Therefore, to evaluate the effects of different kinds of ZBGs, hydrazides were introduced to the ZBG part of **D28**. The synthesized **D28** derivative **D29** and **D30** did not show obvious activity improvement in the Hela nucleus extract screening (Table 1). To study the inhibitory pattern of the derived molecules, the selectivities of molecules **D28**, **D29**, and **D30** were investigated against various isoforms of HDACs using SAHA as a positive control (Table 2). As shown in Table 2, the tested compounds exhibited potential selectivity in inhibition of class I HDACs (HDAC1, 2, and 3) over HDAC6. Remarkably, molecules **D29** and **D30** showed improved inhibitory activities against HDAC 1, 2, and 3 compared with **D28**. The results also revealed that all the selected compounds, especially **D28** and **D29**, have an inhibitory pattern of remarkable HDAC3 selectivity. It is indicated that hydrazide ZBGs are good at increasing the activity and selectivity of HDAC inhibitors.

**TABLE 2:** Enzyme inhibitory selectivity of representative compounds compared with SAHA (IC_50_, µM^a^).

	HDAC1	HDAC2	HDAC3	HDAC6
D28	>1,000	>1,000	24.45 ± 1.24	>1,000
D29	32.59 ± 1.97	183.5 ± 4.32	0.477 ± 0.01	>1,000
D30	1.427 ± 0.02	8.127 ± 0.26	0.100 ± 0.003	>1,000
SAHA	0.0539 ± 0.002	0.152 ± 0.01	0.0397 ± 0.001	ND

a Each value is the mean of three experiments.

### *In Vitro* Antiproliferative Test

To evaluate the *in vitro* anticancer activity of the active compounds, a CCK-8 assay was performed against a series of cancer cell lines. In the K652 cell-based screening, molecule **D28** exhibited antiproliferative activity at a concentration of 2 µM. The hydrazide derivatives of **D28** (**D29** and **D30**) also showed *in vitro* anticancer potential in the test. Therefore, **D28**, **D29,** and **D30** were selected for further evaluation. In screening of cell types that are sensitive to the derived compounds, both solid cancer cell lines were selected in the antiproliferative test (Table 3). Similar to the positive control SAHA, the selected compounds (**D28**, **D29,** and **D30**) showed higher inhibitory potency against hematologic cancer cells than the activity against solid cancer cell lines. Compared with SAHA, molecule **D28** exhibited potency in inhibiting the growth of the test cell lines, especially in inhibition of the hematologic K562, U266 and U937 cell lines. Although compound **D29** and **D30** exhibited significantly increased enzyme inhibitory activities compared with the hydroxamic acid containing **D28**, the antiproliferative activities of **D29** and **D30** decreased markedly in the cancer cell-based test. Compound **D29** and **D30** did not show effective potency in the growth inhibition of the tested solid tumor cell lines, such as Fadu, MDA-MB-231, MDA-MB-468, A549, A2780, and HepG2 cells. The results revealed that hydrazide substitutions of **D28** lead to a decrease in *in vitro* anticancer effects. Among the derived compounds, molecule **D28** could be used as a lead compound for further evaluation.

**TABLE 3:** Antiproliferative activities of representative molecules against various cancer cell lines (IC_50_, μM^a^).

Cell	Cancer type	D28	D29	D30	SAHA
Hematologic cancer cells					
K562	Leukemia	1.02 ± 0.01	5.27 ± 0.14	7.66 ± 0.25	1.55 ± 0.02
U266	Leukemia	1.08 ± 0.02	2.87 ± 0.11	3.68 ± 0.16	1.24 ± 0.08
U937	Leukemia	1.11 ± 0.03	4.22 ± 0.08	5.87 ± 0.24	1.68 ± 0.03
Solid cancer cells					
MCF-7	Breast cancer	5.66 ± 0.26	12.55 ± 0.97	18.76 ± 1.22	5.77 ± 0.15
Fadu	Hypopharyngeal carcinoma	3.22 ± 0.14	>50	>50	2.17 ± 0.92
MDA-MB-231	Breast cancer	4.15 ± 0.22	>50	>50	4.21 ± 0.35
MDA-MB-468	Breast cancer	2.89 ± 0.13	>50	>50	3.75 ± 0.21
A549	Lung carcinoma	2.83 ± 0.17	>50	>50	5.88 ± 0.45
A2780	Ovarian cancer	3.86 ± 0.29	>50	>50	4.56 ± 0.24
HepG2	Hepatocellular carcinoma	2.16 ± 0.13	>50	>50	1.87 ± 0.11

a Each value is the mean of three experiments.

### Cell Cycle Analysis

**FIGURE 3:**
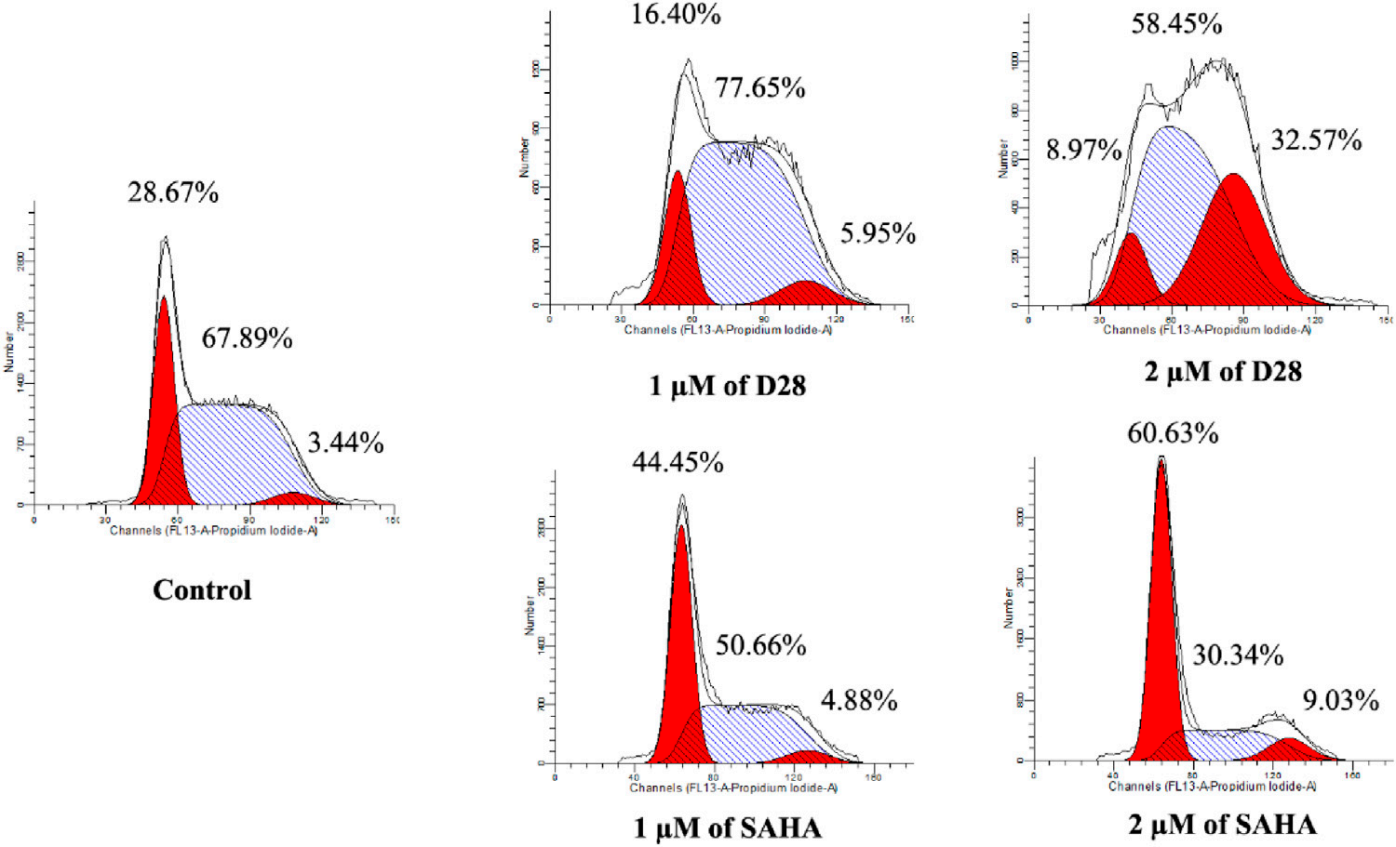
Molecule **D28** induces G2/M cell cycle arrest in K562 cells. Cells were treated with 1.0 and 2.0 µM of **D28** and SAHA for 24 h, and then flow cytometry was used to detect the percentage of cells in various phases of the cell cycle.

Deregulation of the cell cycle divided into G0/G1 phase, S phase, and G2/M phase is one of the most frequent alterations during cancer development. Thus, the cell cycle analysis is usually performed in the anticancer evaluation. Herein, the most sensitive K562 cell line was utilized to investigate the effects of active molecule **D28** on the cell cycle. After treating K562 cells with 1 and 2 µM of **D28** and SAHA for 24 h, the cell cycle distribution was analyzed by flow cytometry. The results revealed different effects of **D28** on the K562 cell cycle compared with SAHA (Figure 3). Molecule **D28** increased cell proportion at the G2/M phase in a dose-dependent manner, while SAHA induced cell cycle arrest at G0/G1 phase. As shown in Figure 3, molecule **D28** increased the G2/M phase ratio of K562 cells from 3.44% of the control to 5.95% and 32.57% at a dose of 1 and 2 μM, respectively. It is suggested that the promotion of G2/M phase cell cycle arrest contributes to the anticancer effects of compound **D28**.

### Cellular Apoptosis Study

**FIGURE 4:**
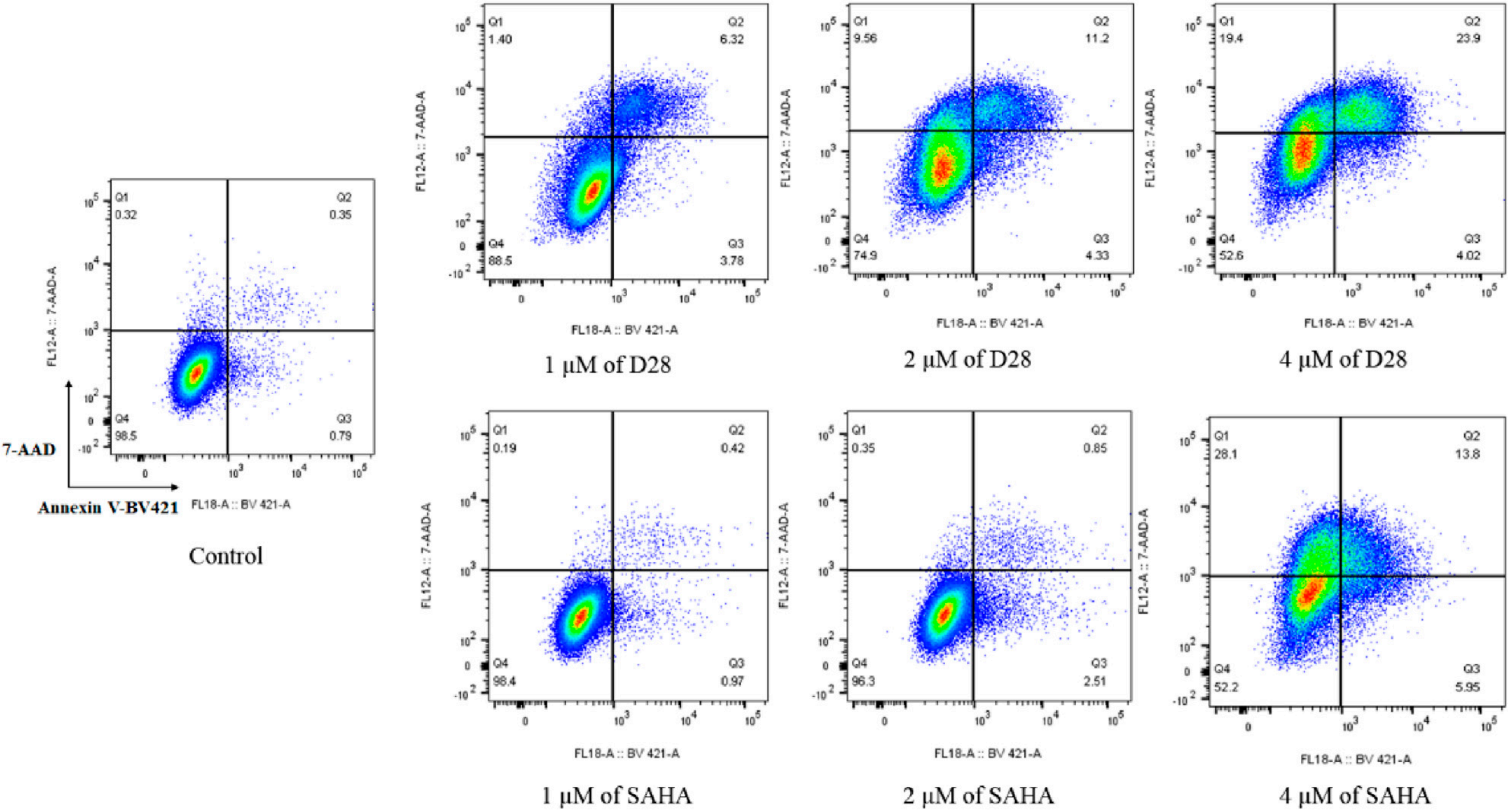
Molecule **D28** induces cellular apoptosis of K562 cells. Cells were treated with 1.0, 2.0 and 4.0 µM of **D28** and SAHA for 24 h, and cell apoptosis was determined by flow cytometric analysis.

Cancer is characterized by too little occurrence of apoptosis. Therefore, induction of apoptosis plays an important role in the treatment of cancer. To evaluate the effects of **D28** on cell apoptosis, K562 cells were treated with various concentrations (1 μM, 2, and 4 µM) of **D28** and SAHA, respectively. The results showed that molecule **D28** promoted apoptosis K562 cells in a dose-dependent manner compared with SAHA (Figure 4). Significantly, molecule **D28** increased apoptotic cell proportion from 1.14% of the control to 10.10%, 15.53%, and 27.92% at a dose of 1, 2, and 4 μM, respectively, compared with SAHA (apoptotic rate of 1.39%, 3.36%, 19.75% at doses of 1, 2, and 4 μM, respectively). The results revealed the important role of apoptosis in the anticancer effects of molecule **D28**.

## Conclusion

Inhibition of HDACs has been extensively studied in the development of anticancer drugs. The discovery of selective HDAC inhibitors has emerged as a hotspot in the epigenetic therapy (He et al., 2020). Selective inhibitors are considered to be promising for cancer treatment by targeting a particular tumor type or a specific mechanism in the tumorigenesis. Compared with non-selective inhibitors, the isoform-selective HDAC inhibitors are considered to be characterized by therapeutic specificity and good safety profiles. HDAC3, the unique HDAC isoform that binds to the nuclear receptor corepressor NCOR1/SMRT, has been revealed to play a specific role in the emergence and development of cancer (Zhang et al., 2019). Selective inhibition of HDAC3 has shown a vast prospect in the treatment of cancer.

In the discovery of novel HDAC inhibitors for the treatment of cancer, the 2-substituted phenylquinoline-4-carboxylic acid group was introduced to the cap moiety of HDAC inhibitors. A total of 30 final compounds were synthesized for the SAR analysis, and various types of ZBGs (hydroxamic acid and hydrazide) were evaluated in the activity test. In the HDAC inhibitory assay, molecule **D28** exhibited HDAC3 selectivity (IC_50_ value of 24.45 µM) without inhibition of HDAC1, 2, and 6. The hydrazide derivative of **D28** showed remarkably improved inhibitory activities compared with **D28**, especially **D29** with IC_50_ values of 32.59, 183.5, 0.477, and >1,000 µM against HDAC1, 2, 3, and 6, respectively. It is indicated that the hydrazide ZBG has the advantage of high potency and HDAC3 selectivity. In the *in vitro* anticancer test, molecule **D28** exhibited good potency in the growth inhibition of tested cell lines with IC_50_ values of 1.02, 1.08, 1.11, 5.66, 3.22, 4.15, 2.89, 2.83, 3.86, and 2.16 µM against K562, U266, U937, MCF-7, Fadu, MDA-MB-231, MDA-MB-468, A549, A2780 and HepG2 cells compared with SAHA. However, molecules **D29** and **D30** with hydrazide ZBG exhibited reduced antiproliferative activities compared with **D28** in the *in vitro* anticancer test. In the flow cytometry, promotion of G2/M cell cycle arrest and induction of apoptosis were revealed to be involved in the anticancer effects of molecule **D28**. Collectively, the current results exhibited the potential of HDAC3 selective inhibition in the treatment of cancer. The discovered HDAC3 selective inhibitors **D28** and **D29** could be utilized as lead compounds for further structural modification in the anticancer drug development.

## Materials and Methods

All chemicals were obtained from commercial suppliers and can be used without further refinement. All reactions were detected by TLC using a 0.25 mm silica gel plate (60GF-254). UV light and ferric chloride were used to show TLC spots. All the synthesized target compounds were classified to be of new structures that have not been previously synthesized. ^1^H NMR and ^13^C NMR spectra were recorded on a Bruker DRX spectrometer at 500 MHz, using TMS as an internal standard. High-resolution mass spectra were recorded using a Thermo Scientific Q Exactive hybrid quadrupole-orbitrap mass spectrometer from Weifang Medical University.

Preparation of **B1** and its analogs: derivatives **B2**–**B28** were prepared as described for **B1** (see below).

2-Phenylquinoline-4-carboxylic acid (**B1**). Isatin (0.5 g, 3.4 mmol) was dissolved in 10 ml 33% KOH solution. Then, 20 ml of acetophenone (0.45 g, 3.74 mmol) ethanol solution was slowly added, and the mixture refluxed at 85°C for 8 h. The solvent was removed by rotary evaporator, and 100 ml of water was added, then 10 ml of 3 M HCl was used to adjust the PH to 5–6. Compound **B1** was obtained by filtration as yellow powder (0.3 g, 35% yield). HRMS C_16_H_11_NO_2_ [M + H^+^] calc. 250.08233 found 250.08180. ^1^H NMR (400 MHz, DMSO) δ 8.67 (d, *J* = 8.5 Hz, 1H), 8.48 (s, 1H), 8.31 (d, *J* = 7.4 Hz, 2H), 8.18 (d, *J* = 8.4 Hz, 1H), 7.87 (t, *J* = 7.6 Hz, 1H), 7.72 (t, *J* = 7.6 Hz, 1H), 7.57 (dq, *J* = 14.1, 7.0 Hz, 3H).

### 2-(4-Bromophenyl)Quinoline-4-Carboxylic Acid (**B2**)

Crystallization in EtOAc as a white solid (0.27g, 21.2% yield). HRMS C_16_H_10_BrNO_2_ [M + H^+^] calc. 327.99285 found 327.99585. ^1^H NMR (400 MHz, DMSO) δ 14.07 (s, 1H), 8.65 (d, *J* = 8.5 Hz, 1H), 8.48 (s, 1H), 8.28 (d, *J* = 8.0 Hz, 2H), 8.18 (d, *J* = 8.4 Hz, 1H), 7.87 (s, 1H), 7.82–7.67 (m, 3H).

### 2-(4-Chlorophenyl)Quinoline-4-Carboxylic Acid (**B-3**).

Crystallization in EtOAc as a white solid (0.28 g, 29.0% yield). HRMS C_16_H_10_ClNO_2_ [M + H^+^] calc. 284.04336 found 284.04630. ^1^H NMR (400 MHz, DMSO) δ 8.66 (d, *J* = 8.5 Hz, 1H), 8.48 (s, 1H), 8.35 (d, *J* = 8.2 Hz, 2H), 8.18 (d, *J* = 8.4 Hz, 1H), 7.88 (t, *J* = 7.6 Hz, 1H), 7.73 (t, *J* = 7.6 Hz, 1H), 7.64 (d, *J* = 8.2 Hz, 2H).

### 2-(4-Fluorophenyl)Quinoline-4-Carboxylic Acid (**B4**).

Crystallization in EtOAc as a white solid (0.26 g, 28.5% yield). HRMS C_16_H_10_FNO_2_ [M + H^+^] calc. 268.07291 found 268.07605. ^1^H NMR (400 MHz, DMSO) δ 14.00 (s, 1H), 8.69–8.56 (m, 1H), 8.39 (dd, *J* = 23.2, 12.3 Hz, 2H), 8.26 (d, *J* = 8.1 Hz, 1H), 8.13 (dd, *J* = 17.7, 8.5 Hz, 1H), 7.94–7.77 (m, 1H), 7.72–7.62 (m, 1H), 7.40 (t, *J* = 8.6 Hz, 1H), 7.10 (d, *J* = 8.6 Hz, 1H).

### 2-(4-(Methylthio)Phenyl)Quinoline-4-Carboxylic Acid (**B5**)

Crystallization in EtOAc as a yellow solid (0.52 g, 51.7% yield). HRMS C_17_H_13_NO_2_S [M + H^+^] calc. 296.07005 found 296.07318. ^1^H NMR (400 MHz, DMSO) δ 8.63 (d, *J* = 8.5 Hz, 1H), 8.39 (s, 1H), 8.26 (d, *J* = 8.2 Hz, 2H), 8.12 (d, *J* = 8.2 Hz, 1H), 7.82 (s, 1H), 7.66 (s, 1H), 7.44 (d, *J* = 8.0 Hz, 2H), 2.56 (s, 3H).

### 2-(4-Methoxyphenyl)Quinoline-4-Carboxylic Acid (**B6**)

Crystallization in EtOAc as a white solid (0.60 g, 63.0% yield). HRMS C_17_H_13_NO_3_ [M + H^+^] calc. 280.09290 found 280.09592. ^1^H NMR (400 MHz, DMSO) δ 13.97 (s, 1H), 8.62 (d, *J* = 8.5 Hz, 1H), 8.41 (s, 1H), 8.28 (d, *J* = 8.5 Hz, 2H), 8.12 (d, *J* = 8.4 Hz, 1H), 7.83 (t, *J* = 7.6 Hz, 1H), 7.66 (t, *J* = 7.6 Hz, 1H), 7.13 (d, *J* = 8.5 Hz, 2H), 3.85 (d, *J* = 7.7 Hz, 3H).

### 2-(4-(Trifluoromethyl)Phenyl)Quinoline-4-Carboxylic Acid (**B7**)

Crystallization in EtOAc as a white solid (0.36 g, 33.4% yield). HRMS C_17_H_10_F_3_NO_2_ [M + H^+^] calc. 318.06972 found 318.07202. ^1^H NMR (400 MHz, DMSO) δ 14.13 (s, 1H), 8.68 (d, *J* = 8.5 Hz, 1H), 8.53 (d, *J* = 8.7 Hz, 3H), 8.21 (d, *J* = 8.4 Hz, 1H), 7.91 (dd, *J* = 20.8, 8.0 Hz, 3H), 7.76 (t, *J* = 7.6 Hz, 1H).

### 2-(3-Chlorophenyl)Quinoline-4-Carboxylic Acid (**B8**)

Crystallization in EtOAc as a white solid (0.56 g, 58.0% yield). HRMS C_16_H_10_ClNO_2_ [M + H^+^] calc. 284.04336 found 284.04587. ^1^H NMR (400 MHz, DMSO) δ 14.09 (s, 1H), 8.64 (d, *J* = 8.4 Hz, 1H), 8.49 (s, 1H), 8.37 (s, 1H), 8.28 (d, *J* = 3.5 Hz, 1H), 8.20 (d, *J* = 8.4 Hz, 1H), 7.88 (t, *J* = 7.6 Hz, 1H), 7.73 (t, *J* = 7.6 Hz, 1H), 7.61 (d, *J* = 4.5 Hz, 2H).

### 2-(m-Tolyl)Quinoline-4-Carboxylic Acid (**B9**)

Crystallization in EtOAc as a white solid (0.44 g, 49.0% yield). HRMS C_17_H_13_NO_2_ [M + H^+^] calc. 264.09798 found 264.10056. ^1^H NMR (400 MHz, DMSO) δ 14.00 (s, 1H), 8.65 (d, *J* = 8.6 Hz, 1H), 8.45 (s, 1H), 8.21–8.05 (m, 3H), 7.86 (t, *J* = 7.5 Hz, 1H), 7.71 (t, *J* = 7.6 Hz, 1H), 7.47 (t, *J* = 7.5 Hz, 1H), 7.36 (d, *J* = 7.6 Hz, 1H), 2.46 (s, 3H).

### 2-(2-Fluorophenyl)Quinoline-4-Carboxylic Acid (**B10**)

Crystallization in EtOAc as a white solid (0.76 g, 59.7% yield). HRMS C_16_H_10_FNO_2_ [M + H^+^] calc. 268.07291 found 268.07291. ^1^H NMR (400 MHz, DMSO) δ 14.02 (s, 1H), 8.74 (d, *J* = 8.4 Hz, 1H), 8.31 (s, 1H), 8.19 (d, *J* = 8.4 Hz, 1H), 8.11 (t, *J* = 7.8 Hz, 1H), 7.89 (t, *J* = 7.7 Hz, 1H), 7.76 (t, *J* = 7.7 Hz, 1H), 7.65–7.54 (m, 1H), 7.42 (dd, *J* = 13.4, 5.8 Hz, 2H).

### 2-(3-Methoxyphenyl)Quinoline-4-Carboxylic Acid (**B11**)

Crystallization in EtOAc as a white solid (0.74 g, 65.5% yield). HRMS C_17_H_13_NO_3_ [M + H^+^] calc. 280.09290 found 280.09586. ^1^H NMR (400 MHz, DMSO) δ 14.03 (s, 1H), 8.64 (d, *J* = 8.5 Hz, 1H), 8.44 (s, 1H), 8.17 (d, *J* = 8.5 Hz, 1H), 7.93–7.80 (m, 3H), 7.71 (t, *J* = 7.6 Hz, 1H), 7.50 (t, *J* = 7.9 Hz, 1H), 7.11 (d, *J* = 8.3 Hz, 1H), 3.89 (s, 3H).

### 2-(3,5-Difluorophenyl)Quinoline-4-Carboxylic Acid (**B12**)

Crystallization in EtOAc as a white solid (0.84 g, 67.9% yield). HRMS C_16_H_9_F_2_NO_2_ [M + H^+^] calc. 286.06349 found 286.16583. ^1^H NMR (400 MHz, DMSO) δ 14.09 (s, 1H), 8.63 (d, *J* = 8.5 Hz, 1H), 8.52 (s, 1H), 8.20 (d, *J* = 8.4 Hz, 1H), 8.06 (d, *J* = 7.9 Hz, 2H), 7.89 (t, *J* = 7.7 Hz, 1H), 7.75 (t, *J* = 7.7 Hz, 1H), 7.44 (t, *J* = 8.8 Hz, 1H).

### 2-(2-Methoxyphenyl)Quinoline-4-Carboxylic Acid (**B13**)

Crystallization in EtOAc as a white solid (0.68 g, 51.1% yield). HRMS C_17_H_13_NO_3_ [M + H^+^] calc. 280.09290 found 280.09583. ^1^H NMR (400 MHz, DMSO) δ 13.88 (s, 1H), 8.70 (d, *J* = 8.5 Hz, 1H), 8.35 (s, 1H), 8.14 (d, *J* = 8.4 Hz, 1H), 7.84 (t, *J* = 6.4 Hz, 2H), 7.72 (t, *J* = 7.7 Hz, 1H), 7.51 (t, *J* = 7.8 Hz, 1H), 7.24 (d, *J* = 8.3 Hz, 1H), 7.14 (t, *J* = 7.4 Hz, 1H), 3.88 (s, 3H).

### 2-(2-Chlorophenyl)Quinoline-4-Carboxylic Acid (**B14**)

Crystallization in EtOAc as a white solid (0.78 g, 49.0% yield). HRMS C_16_H_10_ClNO_2_ [M + H^+^] calc. 284.04336 found 284.04639. ^1^H NMR (400 MHz, DMSO) δ 14.04 (s, 1H), 8.75 (d, *J* = 8.5 Hz, 1H), 8.16 (d, *J* = 9.4 Hz, 2H), 7.89 (t, *J* = 7.5 Hz, 1H), 7.81–7.71 (m, 2H), 7.66 (d, *J* = 7.8 Hz, 1H), 7.55 (dd, *J* = 9.3, 5.7 Hz, 2H).

### 2-(3-Fluorophenyl)Quinoline-4-Carboxylic Acid (**B15**)

Crystallization in EtOAc as a white solid (0.64 g, 49.0% yield). HRMS C_16_H_10_FNO_2_ [M + H^+^] calc. 268.07291 found 268.07581. ^1^H NMR (400 MHz, DMSO) δ 14.06 (s, 1H), 8.65 (d, *J* = 8.5 Hz, 1H), 8.50 (s, 1H), 8.17 (dd, *J* = 21.4, 11.4 Hz, 3H), 7.88 (t, *J* = 7.6 Hz, 1H), 7.74 (t, *J* = 7.7 Hz, 1H), 7.63 (dd, *J* = 14.6, 7.3 Hz, 1H), 7.39 (t, *J* = 8.2 Hz, 1H).

### 2-(o-Tolyl)Quinoline-4-Carboxylic Acid (**B16**)

Crystallization in EtOAc as a white solid (0.66 g, 52.6% yield). HRMS C_17_H_13_NO_2_ [M + H^+^] calc. 264.09798 found 264.10110. ^1^H NMR (400 MHz, DMSO) δ 13.96 (s, 1H), 8.72 (d, *J* = 8.2 Hz, 1H), 8.14 (d, *J* = 8.2 Hz, 1H), 8.04 (s, 1H), 7.87 (t, *J* = 7.3 Hz, 1H), 7.75 (t, *J* = 7.3 Hz, 1H), 7.56 (d, *J* = 6.7 Hz, 1H), 7.35 (d, *J* = 34.3 Hz, 3H), 2.41 (s, 3H).

### 2-(4-Fluoro-3-Methylphenyl)Quinoline-4-Carboxylic Acid (**B17**)

Crystallization in EtOAc as a white solid (0.54 g, 56.3% yield). HRMS C_17_H_12_FNO_2_ [M + H^+^] calc. 282.08856 found 282.08722. ^1^H NMR (400 MHz, DMSO) δ 8.62 (t, *J* = 9.7 Hz, 1H), 8.44 (s, 1H), 8.26 (d, *J* = 7.4 Hz, 1H), 8.21–8.10 (m, 2H), 7.85 (t, *J* = 7.6 Hz, 1H), 7.69 (dd, *J* = 16.9, 9.3 Hz, 1H), 7.33 (t, *J* = 9.1 Hz, 1H), 2.38 (s, 3H).

### 2-(4-(Dimethylamino)Phenyl)Quinoline-4-Carboxylic Acid (**B18**)

Crystallization in EtOAc as a red solid (0.55 g, 55.2% yield). HRMS C_18_H_16_N_2_O_2_ [M + H^+^] calc. 293.12453 found 293.12701. ^1^H NMR (400 MHz, DMSO) δ 8.58 (d, *J* = 8.4 Hz, 1H), 8.33 (s, 1H), 8.17 (d, *J* = 8.4 Hz, 2H), 8.05 (d, *J* = 8.4 Hz, 1H), 7.77 (t, *J* = 7.5 Hz, 1H), 7.59 (t, *J* = 7.6 Hz, 1H), 6.85 (d, *J* = 8.4 Hz, 2H).

### 2-(4-Benzamidophenyl)Quinoline-4-CarboxylicAcid (**B19**)

Crystallization in EtOAc as a white solid (0.27 g, 64.4% yield). HRMS C_23_H_16_N_2_O_3_ [M + H^+^] calc. 369.11945 found 369.12335. ^1^H NMR (400 MHz, DMSO) δ 10.49 (s, 1H), 8.64 (d, *J* = 8.5 Hz, 1H), 8.34–8.20 (m, 3H), 8.07 (d, *J* = 8.3 Hz, 1H), 8.01 (d, *J* = 7.9 Hz, 4H), 7.95 (d, *J* = 8.7 Hz, 1H), 7.76 (t, *J* = 7.5 Hz, 1H), 7.63–7.51 (m, 4H).

### 2-(4-(4-Fluorobenzamido)Phenyl)Quinoline-4-Carboxylic Acid (**B20**)

Crystallization in EtOAc as a white solid (0.41 g, 93.2% yield). HRMS C_23_H_15_FN_2_O_3_ [M + H^+^] calc. 387.11003 found 387.10703. ^1^H NMR (400 MHz, DMSO) δ 10.51 (s, 1H), 8.65 (d, *J* = 8.5 Hz, 1H), 8.48 (s, 1H), 8.34 (d, *J* = 8.3 Hz, 2H), 8.22–7.95 (m, 5H), 7.85 (t, *J* = 7.6 Hz, 1H), 7.69 (t, *J* = 7.6 Hz, 1H), 7.40 (t, *J* = 8.6 Hz, 2H).

### 2-(4-(3-Bromobenzamido)Phenyl)Quinoline-4-Carboxylic Acid (**B21**)

Crystallization in EtOAc as a white solid (0.36 g, 70.1% yield). HRMS C_23_H_15_BrN_2_O_3_ [M + H^+^] calc. 447.02996 found 447.03238. ^1^H NMR (400 MHz, DMSO) δ 10.60 (s, 1H), 8.64 (d, *J* = 7.8 Hz, 1H), 8.50–8.29 (m, 3H), 8.21 (s, 1H), 8.13 (d, *J* = 8.0 Hz, 1H), 7.99 (t, *J* = 14.1 Hz, 4H), 7.82 (s, 2H), 7.66 (d, *J* = 6.6 Hz, 1H), 7.54 (d, *J* = 7.5 Hz, 1H).

### 2-(4-(2-Fluorobenzamido)Phenyl)Quinoline-4-Carboxylic Acid (**B22**)

Crystallization in EtOAc as a white solid (0.44 g, 85.5% yield). HRMS C_23_H_15_FN_2_O_3_ [M + H^+^] calc. 387.11003 found 387.10721. ^1^H NMR (400 MHz, DMSO) δ 10.69 (s, 1H), 8.63 (d, *J* = 7.8 Hz, 1H), 8.58–8.22 (m, 3H), 8.14 (d, *J* = 7.8 Hz, 1H), 7.95 (d, *J* = 7.0 Hz, 2H), 7.82 (d, *J* = 6.6 Hz, 1H), 7.77–7.49 (m, 3H), 7.50–7.14 (m, 2H).

### 2-(4-(2-Chlorobenzamido)Phenyl)Quinoline-4-Carboxylic Acid (**B23**)

Crystallization in EtOAc as a white solid (0.73 g, 68.6% yield). HRMS C_23_H_15_FN_2_O_3_ [M + H^+^] calc. 403.08047 found 403.07718. ^1^H NMR (400 MHz, DMSO) δ 10.80 (s, 1H), 8.68 (d, *J* = 7.5 Hz, 1H), 8.27 (d, *J* = 7.2 Hz, 2H), 8.14 (s, 1H), 8.03 (d, *J* = 7.7 Hz, 1H), 7.93 (d, *J* = 7.1 Hz, 2H), 7.71 (s, 1H), 7.63 (s, 1H), 7.58 (s, 1H), 7.42 (dd, *J* = 65.7, 22.8 Hz, 4H).

### 2-(4-(2,4-Difluorobenzamido)Phenyl)Quinoline-4-Carboxylic Acid (**B24**)

Crystallization in EtOAc as a white solid (0.47 g, 94.7% yield). HRMS C_23_H_14_F_2_N_2_O_3_ [M + H^+^] calc. 405.10060 found 405.09412. ^1^H NMR (400 MHz, DMSO) δ 10.68 (s, 1H), 8.64 (d, *J* = 8.4 Hz, 1H), 8.43 (s, 1H), 8.33 (d, *J* = 8.3 Hz, 2H), 8.14 (d, *J* = 8.4 Hz, 1H), 7.93 (d, *J* = 8.4 Hz, 2H), 7.88–7.76 (m, 2H), 7.67 (t, *J* = 7.6 Hz, 1H), 7.46 (t, *J* = 9.9 Hz, 1H), 7.26 (t, *J* = 8.4 Hz, 1H), 5.29–5.25 (m, 1H).

### 2-(4-(3,5-Difluorobenzamido)Phenyl)Quinoline-4-Carboxylic Acid (**B25**)

Crystallization in EtOAc as a white solid (0.96 g, 89.4% yield). HRMS C_23_H_14_F_2_N_2_O_3_ [M + H^+^] calc. 405.10060 found 405.10413. ^1^H NMR (400 MHz, DMSO) δ 10.61 (s, 1H), 8.64 (d, *J* = 8.4 Hz, 1H), 8.43 (s, 1H), 8.35 (d, *J* = 8.2 Hz, 2H), 8.14 (d, *J* = 8.3 Hz, 1H), 8.01 (d, *J* = 8.2 Hz, 2H), 7.83 (t, *J* = 7.5 Hz, 1H), 7.80–7.60 (m, 3H), 7.55 (t, *J* = 8.5 Hz, 1H).

### 2-(4-(3-Fluorobenzamido)Phenyl)Quinoline-4-Carboxylic Acid (**B26**)

Crystallization in EtOAc as a white solid (0.67 g, 91.6% yield). HRMS C_23_H_15_FN_2_O_3_ [M + H^+^] calc. 387.11003 found 387.10410. ^1^H NMR (400 MHz, DMSO) δ 10.57 (s, 1H), 8.63 (d, *J* = 8.4 Hz, 1H), 8.41–8.28 (m, 3H), 8.11 (d, *J* = 8.4 Hz, 1H), 8.01 (d, *J* = 8.1 Hz, 2H), 7.90–7.75 (m, 3H), 7.62 (dd, *J* = 14.6, 7.2 Hz, 2H), 7.48 (t, *J* = 8.5 Hz, 1H).

### 2-(2,5-Difluorophenyl)Quinoline-4-Carboxylic Acid (**B27**)

Crystallization in EtOAc as a white solid (0.83 g, 85.3% yield). HRMS C_16_H_9_F_2_NO_2_ [M + H^+^] calc. 286.06349 found 286.06192. ^1^H NMR (400 MHz, DMSO) δ 8.75 (d, *J* = 8.5 Hz, 1H), 8.35 (s, 1H), 8.21 (d, *J* = 8.4 Hz, 1H), 7.91 (t, *J* = 7.5 Hz, 2H), 7.79 (t, *J* = 7.7 Hz, 1H), 7.59–7.33 (m, 2H).

### 2-([1,1′-Biphenyl]-4-yl)Quinoline-4-Carboxylic Acid (**B28**)

Crystallization in EtOAc as a yellow solid (0.98 g, 88.4% yield). HRMS C_22_H_15_NO_2_ [M + H^+^] calc. 326.11363 found 326.11176. ^1^H NMR (400 MHz, DMSO) δ 8.66 (d, *J* = 8.5 Hz, 1H), 8.52 (s, 1H), 8.42 (d, *J* = 7.9 Hz, 2H), 8.19 (d, *J* = 8.5 Hz, 1H), 7.88 (dd, *J* = 14.6, 7.9 Hz, 3H), 7.79 (d, *J* = 7.7 Hz, 2H), 7.71 (t, *J* = 7.6 Hz, 1H), 7.53 (t, *J* = 7.5 Hz, 2H), 7.43 (t, *J* = 7.2 Hz, 1H).

### 2-(4-Amino-Phenyl)-Quinoline-4-Carboxylic Acid (**B29**)

Crystallization in EtOAc as a white solid (0.54 g, 59.9% yield). HRMS C_16_H_12_N_2_O_2_ [M + H]^+^ calc. 265.0320, found 265.0960. ^1^H NMR (400 MHz, DMSO) δ 8.58 (d, J = 8.3 Hz, 1H), 8.32 (s, 1H), 8.05 (d, J = 8.1 Hz, 3H), 7.77 (t, J = 7.3 Hz, 1H), 7.59 (t, J = 7.4 Hz, 1H), 6.72 (d, J = 8.1 Hz, 2H).

### Methyl 4-(4-(2-Phenylquinoline-4-Carbonyl)Piperazin-1-yl)Benzoate (**C1**)

2-Phenylquinoline-4-carboxylic acid (**B1**) (0.51 g, 2.06 mmol) was dissolved in 20 ml of DCM. Under ice bath condition Et_3_N (0.48 g, 4.71 mmol) and TBTU (0.73 g, 2.27 mmol) were added. After 20 min, the ice bath was removed and 4-(Piperazine-1-yl) Methyl Benzoate (0.5 g, 2.27 mmol) was added. After 8 h, the solvent is removed by rotary evaporation, and the concentrated solution was dissolved in 100 ml of EtOAc, washed with NaHCO_3_ (3 × 20 ml) and NaCl (3 × 20 ml), and dried with MgSO_4_. The desired compound (**C1**) was obtained by filtration and recrystallization in EtOAc as a white solid (0.21 g, 23.1% yield). HRMS C_28_H_25_N_3_O_3_ [M + H^+^] calc. 452.19295 found 452.19568. ^1^H NMR (400 MHz, DMSO) δ 8.33 (d, *J* = 7.5 Hz, 2H), 8.20 (s, 1H), 8.16 (d, *J* = 8.3 Hz, 1H), 7.91–7.75 (m, 4H), 7.66 (t, *J* = 7.6 Hz, 1H), 7.57 (dd, *J* = 16.0, 8.8 Hz, 3H), 6.99 (d, *J* = 8.7 Hz, 2H), 3.95 (d, *J* = 19.7 Hz, 2H), 3.77 (s, 3H), 3.59 (s, 2H), 3.35 (s, 2H), 3.29–3.12 (m, 3H).

### Methyl 4-(4-(2-(4-Bromophenyl)Quinoline-4-Carbonyl)Piperazin-1-yl)Benzoate (**C2**)

Crystallization in EtOAc as a yellow solid (0.17 g, 25.1% yield). HRMS C_28_H_24_BrN_3_O_3_ [M + H^+^] calc. 530.10346 found 530.10614. ^1^H NMR (400 MHz, DMSO) δ 8.30 (d, *J* = 8.2 Hz, 2H), 8.23 (s, 1H), 8.16 (d, *J* = 8.8 Hz, 1H), 7.82 (dt, *J* = 18.3, 8.4 Hz, 6H), 7.67 (t, *J* = 7.6 Hz, 1H), 6.99 (d, *J* = 8.6 Hz, 2H), 3.95 (d, *J* = 16.8 Hz, 2H), 3.77 (s, 3H), 3.59 (s, 2H), 3.33 (s, 2H), 3.25 (d, *J* = 18.2 Hz, 2H).

### Methyl 4-(4-(2-(4-Chlorophenyl)Quinoline-4-Carbonyl)Piperazin-1-yl)Benzoate (**C3**)

Crystallization in EtOAc as a white solid (0.33 g, 41.1% yield). HRMS C_28_H_24_ClN_3_O_3_ [M + H^+^] calc. 486.15397 found 486.15885. ^1^H NMR (400 MHz, DMSO) δ 8.37 (d, *J* = 8.2 Hz, 1H), 8.14 (dd, *J* = 12.0, 8.6 Hz, 1H), 7.86 (t, *J* = 9.6 Hz, 3H), 7.80 (d, *J* = 8.8 Hz, 2H), 7.68 (d, *J* = 8.0 Hz, 1H), 7.64 (d, *J* = 8.6 Hz, 1H), 7.52 (t, *J* = 7.6 Hz, 1H), 7.06 (d, *J* = 8.7 Hz, 1H), 6.99 (d, *J* = 8.5 Hz, 2H), 3.78 (d, *J* = 7.6 Hz, 4H), 3.60 (dd, *J* = 21.8, 17.3 Hz, 4H), 3.35 (s, 4H).

### Methyl 4-(4-(2-(4-Fluorophenyl)Quinoline-4-Carbonyl)Piperazin-1-yl)Benzoate (**C4**)

Crystallization in EtOAc as a white solid (0.13 g, 57.6% yield). HRMS C_28_H_24_FN_3_O_3_ [M + H^+^] calc. 470.18352 found 470.18607. ^1^H NMR (400 MHz, DMSO) δ 8.48–8.35 (m, 1H), 8.29 (d, *J* = 8.5 Hz, 1H), 8.25–8.06 (m, 2H), 7.90–7.73 (m, 4H), 7.64 (dd, *J* = 19.5, 7.7 Hz, 1H), 7.40 (t, *J* = 8.5 Hz, 1H), 7.09 (d, *J* = 8.6 Hz, 1H), 6.99 (d, *J* = 8.5 Hz, 2H), 3.95 (d, *J* = 17.2 Hz, 2H), 3.77 (s, 3H), 3.59 (s, 2H), 3.35 (s, 2H), 3.25 (d, *J* = 19.8 Hz, 2H).

### Methyl 4-(4-(2-(4-(Methylthio)Phenyl)Quinoline-4-Carbonyl)Piperazin-1-yl)Benzoate (**C5**)

Crystallization in EtOAc as a yellow solid (0.21 g, 41.7% yield). HRMS C_29_H_27_N_3_O_3_S [M + H^+^] calc. 498.18067 found 498.18253. ^1^H NMR (400 MHz, DMSO) δ 8.29 (d, *J* = 8.1 Hz, 2H), 8.21–8.07 (m, 2H), 7.82 (dd, *J* = 19.3, 8.4 Hz, 4H), 7.64 (t, *J* = 7.6 Hz, 1H), 7.43 (d, *J* = 8.3 Hz, 2H), 6.99 (d, *J* = 8.6 Hz, 2H), 3.95 (d, *J* = 22.5 Hz, 2H), 3.78 (d, *J* = 8.2 Hz, 3H), 3.59 (s, 2H), 3.29–3.12 (m, 4H).

### Methyl 4-(4-(2-(4-Methoxyphenyl)Quinoline-4-Carbonyl)Piperazin-1-yl)Benzoate (**C6**)

Crystallization in EtOAc as a white solid (0.18 g, 30.3% yield). HRMS C_29_H_27_N_3_O_4_ [M + H^+^] calc. 482.20351 found 482.19928. ^1^H NMR (400 MHz, DMSO) δ 8.32 (d, *J* = 8.2 Hz, 2H), 8.17–8.07 (m, 2H), 7.82 (dd, *J* = 11.9, 8.5 Hz, 4H), 7.63 (d, *J* = 7.7 Hz, 1H), 7.12 (d, *J* = 8.3 Hz, 2H), 6.99 (d, *J* = 8.5 Hz, 2H), 4.01–3.74 (m, 8H), 3.60 (d, *J* = 4.6 Hz, 2H), 3.35 (s, 1H), 3.33–3.14 (m, 3H).

### Methyl 4-(4-(2-(4-(Trifluoromethyl)Phenyl)Quinoline-4-Carbonyl)Piperazin-1-yl)Benzoate (**C7**)

Crystallization in EtOAc as a white solid (0.16 g, 32.4% yield). HRMS C_29_H_24_F_3_N_3_O_3_ [M + H^+^] calc. 520.18033 found 520.18274. ^1^H NMR (400 MHz, DMSO) δ 8.56 (d, *J* = 8.1 Hz, 2H), 8.31 (s, 1H), 8.20 (d, *J* = 8.7 Hz, 1H), 8.00–7.83 (m, 4H), 7.80 (d, *J* = 8.4 Hz, 2H), 7.71 (t, *J* = 7.5 Hz, 1H), 6.99 (d, *J* = 8.7 Hz, 2H), 3.96 (d, *J* = 18.8 Hz, 2H), 3.77 (s, 3H), 3.60 (s, 2H), 3.33 (s, 2H), 3.21 (s, 2H).

### Methyl 4-(4-(2-(3-Chlorophenyl)Quinoline-4-Carbonyl)Piperazin-1-yl)Benzoate (**C8**)

Crystallization in EtOAc as a white solid (0.25 g, 48.5% yield). HRMS C_28_H_24_ClN_3_O_3_ [M + H^+^] calc. 486.15397 found 486.15674. ^1^H NMR (400 MHz, DMSO) δ 8.40 (s, 1H), 8.30 (d, *J* = 17.4 Hz, 2H), 8.19 (d, *J* = 8.7 Hz, 1H), 7.87 (t, *J* = 7.2 Hz, 2H), 7.80 (d, *J* = 8.6 Hz, 2H), 7.68 (t, *J* = 7.7 Hz, 1H), 7.61 (d, *J* = 4.6 Hz, 2H), 6.99 (d, *J* = 8.6 Hz, 2H), 3.95 (d, *J* = 18.4 Hz, 2H), 3.77 (s, 3H), 3.60 (s, 2H), 3.34 (s, 2H), 3.22 (s, 2H).

### Methyl 4-(4-(2-(m-Tolyl)Quinoline-4-Carbonyl)Piperazin-1-yl)Benzoate (**C9**)

Crystallization in EtOAc as a white solid (0.24 g, 45.2% yield). HRMS C_29_H_27_N_3_O_3_ [M + H^+^] calc. 466.20880 found 466.21140. ^1^H NMR (400 MHz, DMSO) δ 8.22–8.08 (m, 4H), 7.90–7.76 (m, 4H), 7.65 (t, *J* = 7.5 Hz, 1H), 7.45 (t, *J* = 7.6 Hz, 1H), 7.35 (d, *J* = 7.5 Hz, 1H), 6.99 (d, *J* = 8.6 Hz, 2H), 3.95 (d, *J* = 29.7 Hz, 2H), 3.77 (s, 3H), 3.59 (s, 2H), 3.33 (s, 2H), 3.25 (d, *J* = 21.0 Hz, 2H), 2.45 (s, 3H).

### Methyl 4-(4-(2-(2-Fluorophenyl)Quinoline-4-Carbonyl)Piperazin-1-yl)Benzoate (**C10**)

Crystallization in EtOAc as a white solid (0.13 g, 24.7% yield). HRMS C_28_H_24_FN_3_O_3_ [M + H^+^] calc. 470.18352 found 470.18607. ^1^H NMR (400 MHz, DMSO) δ 8.18 (d, *J* = 8.3 Hz, 1H), 8.08 (t, *J* = 7.8 Hz, 1H), 7.90 (dd, *J* = 14.5, 7.3 Hz, 3H), 7.79 (d, *J* = 8.5 Hz, 2H), 7.71 (t, *J* = 7.5 Hz, 1H), 7.63–7.54 (m, 1H), 7.42 (dd, *J* = 14.0, 7.0 Hz, 2H), 6.98 (d, *J* = 8.6 Hz, 2H), 4.02–3.83 (m, 2H), 3.77 (s, 3H), 3.58 (s, 2H), 3.34 (s, 1H), 3.22 (s, 3H).

### Methyl 4-(4-(2-(3-Methoxyphenyl)Quinoline-4-Carbonyl)Piperazin-1-yl)Benzoate (**C11**)

Crystallization in EtOAc as a white solid (0.20 g, 38.8% yield). HRMS C_29_H_27_N_3_O_4_ [M + H^+^] calc. 482.20351 found 482.20605. ^1^H NMR (400 MHz, DMSO) δ 8.21 (s, 1H), 8.16 (d, *J* = 8.4 Hz, 1H), 7.91 (d, *J* = 7.7 Hz, 1H), 7.90–7.82 (m, 3H), 7.80 (d, *J* = 8.6 Hz, 2H), 7.66 (t, *J* = 7.5 Hz, 1H), 7.48 (t, *J* = 8.0 Hz, 1H), 7.11 (d, *J* = 7.4 Hz, 1H), 6.99 (d, *J* = 8.7 Hz, 2H), 3.89 (s, 5H), 3.77 (s, 3H), 3.60 (s, 2H), 3.34 (s, 1H), 3.24 (d, *J* = 21.3 Hz, 2H), 1.99 (s, 1H).

### Methyl 4-(4-(2-(3,5-Difluorophenyl)Quinoline-4-Carbonyl)Piperazin-1-yl)Benzoate (**C12**)

Crystallization in EtOAc as a white solid (0.20 g, 39.0% yield). HRMS C_28_H_23_F_2_N_3_O_3_ [M + H^+^] calc. 488.17410 found 488.17651. ^1^H NMR (400 MHz, DMSO) δ 8.31 (s, 1H), 8.19 (d, *J* = 8.7 Hz, 1H), 8.09 (d, *J* = 7.3 Hz, 2H), 7.94–7.85 (m, 2H), 7.80 (d, *J* = 8.6 Hz, 2H), 7.70 (t, *J* = 7.8 Hz, 1H), 7.42 (d, *J* = 9.5 Hz, 1H), 7.00 (d, *J* = 8.8 Hz, 2H), 3.95 (s, 2H), 3.77 (s, 3H), 3.60 (s, 2H), 3.34 (s, 2H), 3.25 (d, *J* = 16.6 Hz, 2H).

### Methyl 4-(4-(2-(2-Methoxyphenyl)Quinoline-4-Carbonyl)Piperazin-1-yl)Benzoate (**C13**)

Crystallization in EtOAc as a white solid (0.21 g, 40.7% yield). HRMS C_29_H_27_N_3_O_4_ [M + H^+^] calc. 482.20351 found 482.20609. ^1^H NMR (400 MHz, DMSO) δ 8.12 (t, *J* = 10.6 Hz, 1H), 7.86 (ddd, *J* = 26.1, 16.7, 10.5 Hz, 6H), 7.66 (t, *J* = 7.6 Hz, 1H), 7.49 (t, *J* = 7.8 Hz, 1H), 7.21 (d, *J* = 8.3 Hz, 1H), 7.14 (t, *J* = 7.4 Hz, 1H), 6.98 (d, *J* = 8.4 Hz, 2H), 3.95 (d, *J* = 6.9 Hz, 2H), 3.87 (s, 3H), 3.78 (s, 3H), 3.56 (t, *J* = 4.6 Hz, 2H), 3.47 (s, 1H), 3.18 (d, *J* = 4.8 Hz, 3H).

### Methyl 4-(4-(2-(2-Chlorophenyl)Quinoline-4-Carbonyl)Piperazin-1-yl)Benzoate (**C14**)

Crystallization in EtOAc as a white solid (0.20 g, 38.9% yield). HRMS C_28_H_24_ClN_3_O_3_ [M + H^+^] calc. 486.15397 found 486.15686. ^1^H NMR (400 MHz, DMSO) δ 8.15 (d, *J* = 8.5 Hz, 1H), 7.98–7.85 (m, 2H), 7.80 (d, *J* = 10.2 Hz, 3H), 7.73 (t, *J* = 7.6 Hz, 2H), 7.65 (d, *J* = 4.8 Hz, 1H), 7.57–7.51 (m, 2H), 6.99 (d, *J* = 8.7 Hz, 2H), 3.94 (s, 2H), 3.77 (s, 3H), 3.57 (s, 2H), 3.34 (s, 2H), 3.31–3.17 (m, 2H).

### Methyl 4-(4-(2-(3-Fluorophenyl)Quinoline-4-Carbonyl)Piperazin-1-yl)Benzoate (**C15**)

Crystallization in EtOAc as a white solid (0.30 g, 57.0% yield). HRMS C_28_H_24_FN_3_O_3_ [M + H^+^] calc. 470.18352 found 470.18597. ^1^H NMR (400 MHz, DMSO) δ 8.26 (s, 1H), 8.24–8.12 (m, 3H), 7.87 (t, *J* = 7.2 Hz, 2H), 7.80 (d, *J* = 8.7 Hz, 2H), 7.65 (dt, *J* = 14.6, 7.8 Hz, 2H), 7.38 (t, *J* = 8.4 Hz, 1H), 6.99 (d, *J* = 8.8 Hz, 2H), 3.94 (s, 2H), 3.77 (s, 3H), 3.60 (s, 2H), 3.34 (s, 2H), 3.25 (d, *J* = 19.1 Hz, 2H).

### Methyl 4-(4-(2-(o-Tolyl)Quinoline-4-Carbonyl)Piperazin-1-yl)Benzoate (**C16**)

Crystallization in EtOAc as a white solid (0.28 g, 52.7% yield). HRMS C_29_H_27_N_3_O_3_ [M + H^+^] calc. 466.20860 found 466.21133. ^1^H NMR (400 MHz, DMSO) δ 8.12 (d, *J* = 8.4 Hz, 1H), 7.93–7.82 (m, 2H), 7.80 (d, *J* = 8.6 Hz, 2H), 7.70 (dd, *J* = 15.2, 7.8 Hz, 2H), 7.56 (d, *J* = 7.2 Hz, 1H), 7.44–7.31 (m, 3H), 6.99 (d, *J* = 8.7 Hz, 2H), 3.93 (d, *J* = 37.1 Hz, 2H), 3.77 (s, 3H), 3.54 (d, *J* = 28.2 Hz, 2H), 3.34 (s, 2H), 3.31–3.10 (m, 2H), 2.42 (d, *J* = 10.6 Hz, 3H).

### Methyl 4-(4-(2-(4-Fluoro-3-Methylphenyl)Quinoline-4-Carbonyl)Piperazin-1-yl)Benzoate (**C17**)

Crystallization in EtOAc as a white solid (0.34 g, 43.9% yield). HRMS C_29_H_26_FN_3_O_3_ [M + H^+^] calc. 484.19917 found 484.20154. ^1^H NMR (400 MHz, DMSO) δ 8.29 (d, *J* = 7.2 Hz, 1H), 8.25–8.09 (m, 3H), 7.90–7.77 (m, 4H), 7.65 (t, *J* = 7.5 Hz, 1H), 7.32 (t, *J* = 9.0 Hz, 1H), 6.98 (d, *J* = 8.6 Hz, 2H), 4.07–3.84 (m, 2H), 3.77 (s, 4H), 3.59 (t, *J* = 4.7 Hz, 2H), 3.31–3.13 (m, 3H), 2.37 (s, 3H).

### Methyl 4-(4-(2-(4-(Dimethylamino)Phenyl)Quinoline-4-Carbonyl)Piperazin-1-yl)Benzoate (**C18**)

Crystallization in EtOAc as a red solid (0.30 g, 35.4% yield). HRMS C_30_H_30_N_4_O_3_ [M + H^+^] calc. 495.23515 found 495.23041. ^1^H NMR (400 MHz, DMSO) δ 8.21 (d, *J* = 8.4 Hz, 2H), 8.05 (d, *J* = 10.8 Hz, 2H), 7.78 (dd, *J* = 15.2, 7.5 Hz, 4H), 7.55 (t, *J* = 7.5 Hz, 1H), 6.99 (d, *J* = 8.5 Hz, 2H), 6.85 (d, *J* = 8.5 Hz, 2H), 3.95 (d, *J* = 26.6 Hz, 2H), 3.78 (s, 3H), 3.59 (s, 2H), 3.25 (dd, *J* = 37.0, 14.9 Hz, 3H), 3.02 (s, 6H).

### Methyl 4-(4-(2-(4-Benzamidophenyl)Quinoline-4-Carbonyl)Piperazin-1-yl)Benzoate (**C19**)

Crystallization in EtOAc as a white solid (0.28 g, 75.8% yield). HRMS C_35_H_30_N_4_O_4_ [M + H^+^] calc. 571.23006 found 571.23431. ^1^H NMR (400 MHz, DMSO) δ 10.49 (s, 1H), 8.37 (d, *J* = 8.5 Hz, 2H), 8.24–8.07 (m, 2H), 8.01 (dd, *J* = 7.8, 3.9 Hz, 4H), 7.83 (dd, *J* = 19.6, 8.3 Hz, 4H), 7.68–7.49 (m, 4H), 7.00 (d, *J* = 8.7 Hz, 2H), 3.96 (d, *J* = 16.8 Hz, 2H), 3.77 (s, 3H), 3.60 (s, 2H), 3.34 (d, *J* = 3.4 Hz, 3H), 3.24 (s, 1H).

### Methyl 4-(4-(2-(4-(4-Fluorobenzamido)Phenyl)Quinoline-4-Carbonyl)Piperazin-1-yl)Benzoate (**C20**)

Crystallization in EtOAc as a white solid (0.33 g, 71.8% yield). HRMS C_35_H_29_FN_4_O_4_ [M + H^+^] calc. 589.22064 found 589.22241. ^1^H NMR (400 MHz, DMSO) δ 10.49 (s, 1H), 8.36 (d, *J* = 8.5 Hz, 2H), 8.19 (s, 1H), 8.16–8.05 (m, 3H), 7.99 (d, *J* = 8.3 Hz, 2H), 7.83 (dd, *J* = 19.7, 8.1 Hz, 4H), 7.64 (t, *J* = 7.6 Hz, 1H), 7.40 (t, *J* = 8.4 Hz, 2H), 7.00 (d, *J* = 8.6 Hz, 2H), 3.94 (s, 2H), 3.77 (s, 3H), 3.60 (s, 2H), 3.36 (s, 2H), 3.17 (s, 2H).

### Methyl 4-(4-(2-(4-(3-Bromobenzamido)Phenyl)Quinoline-4-Carbonyl)Piperazin-1-yl)Benzoate (**C21**)

Crystallization in EtOAc as a white solid (0.23 g, 52.9% yield). HRMS C_35_H_29_BrN_4_O_4_ [M + H^+^] calc. 649.14057 found 649.14526. ^1^H NMR (400 MHz, DMSO) δ 10.58 (s, 1H), 8.37 (d, *J* = 8.4 Hz, 2H), 8.20 (s, 2H), 8.14 (d, *J* = 8.4 Hz, 1H), 8.00 (d, *J* = 8.1 Hz, 3H), 7.83 (dd, *J* = 20.0, 8.7 Hz, 5H), 7.64 (t, *J* = 7.4 Hz, 1H), 7.53 (t, *J* = 7.9 Hz, 1H), 7.00 (d, *J* = 8.7 Hz, 2H), 3.96 (d, *J* = 16.3 Hz, 2H), 3.77 (s, 3H), 3.60 (s, 2H), 3.35 (s, 2H), 3.24 (s, 2H).

### Methyl 4-(4-(2-(4-(2-Fluorobenzamido)Phenyl)Quinoline-4-Carbonyl)Piperazin-1-yl)Benzoate (**C22**)

Crystallization in EtOAc as a white solid (0.21 g, 46.3% yield). HRMS C_35_H_29_FN_4_O_4_ [M + H^+^] calc. 589.22064 found 589.22437. ^1^H NMR (400 MHz, DMSO) δ 10.67 (s, 1H), 8.36 (d, *J* = 8.3 Hz, 2H), 8.19 (s, 1H), 8.14 (d, *J* = 8.6 Hz, 1H), 7.93 (d, *J* = 8.4 Hz, 2H), 7.83 (dd, *J* = 20.0, 8.3 Hz, 4H), 7.71 (t, *J* = 7.3 Hz, 1H), 7.68–7.57 (m, 2H), 7.44–7.31 (m, 2H), 7.00 (d, *J* = 8.6 Hz, 2H), 4.01–3.86 (m, 2H), 3.77 (s, 3H), 3.60 (s, 2H), 3.35 (s, 2H), 3.24 (s, 2H).

### Methyl 4-(4-(2-(4-(2-Chlorobenzamido)Phenyl)Quinoline-4-Carbonyl)Piperazin-1-yl)Benzoate (**C23**)

Crystallization in EtOAc as a white solid (0.27 g, 45.1% yield). HRMS C_35_H_29_ClN_4_O_4_ [M + H^+^] calc. 605.19109 found 605.19525. ^1^H NMR (400 MHz, DMSO) δ 10.75 (s, 1H), 8.36 (d, *J* = 8.3 Hz, 2H), 8.18 (s, 1H), 8.14 (d, *J* = 8.6 Hz, 1H), 7.92 (d, *J* = 8.3 Hz, 2H), 7.83 (dd, *J* = 20.1, 8.3 Hz, 5H), 7.60 (d, *J* = 7.8 Hz, 2H), 7.51 (dt, *J* = 21.1, 7.3 Hz, 2H), 7.00 (d, *J* = 8.5 Hz, 2H), 3.93 (s, 2H), 3.77 (s, 3H), 3.60 (s, 2H), 3.35 (s, 2H), 3.17 (d, *J* = 5.2 Hz, 2H).

### Methyl 4-(4-(2-(4-(2,4-Difluorobenzamido)Phenyl)Quinoline-4-Carbonyl)Piperazin-1-yl)Benzoate (**C24**)

Crystallization in EtOAc as a white solid (0.14 g, 31.2% yield). HRMS C_35_H_28_F_2_N_4_O_4_ [M + H^+^] calc. 607.21122 found 607.21466. ^1^H NMR (400 MHz, DMSO) δ 10.66 (s, 1H), 8.36 (d, *J* = 8.0 Hz, 2H), 8.26–8.05 (m, 2H), 7.98–7.71 (m, 7H), 7.64 (t, *J* = 7.7 Hz, 1H), 7.46 (t, *J* = 10.5 Hz, 1H), 7.26 (t, *J* = 8.6 Hz, 1H), 7.00 (d, *J* = 8.5 Hz, 2H), 4.06–3.87 (m, 2H), 3.77 (s, 3H), 3.60 (s, 2H), 3.40 (s, 1H), 3.17 (d, *J* = 5.1 Hz, 3H).

### Methyl 4-(4-(2-(4-(3,5-Difluorobenzamido)Phenyl)Quinoline-4-Carbonyl)Piperazin-1-yl)Benzoate (**C25**)

Crystallization in EtOAc as a white solid (0.18 g, 40.1% yield). HRMS C_35_H_28_F_2_N_4_O_4_ [M + H^+^] calc. 607.21122 found 607.21472. ^1^H NMR (400 MHz, DMSO) δ 10.58 (s, 1H), 8.39 (d, *J* = 8.2 Hz, 2H), 8.20 (s, 1H), 8.14 (d, *J* = 8.4 Hz, 1H), 7.99 (d, *J* = 8.2 Hz, 2H), 7.89–7.77 (m, 4H), 7.74 (d, *J* = 7.2 Hz, 2H), 7.64 (t, *J* = 7.6 Hz, 1H), 7.56 (t, *J* = 9.1 Hz, 1H), 6.99 (d, *J* = 8.4 Hz, 2H), 3.96 (d, *J* = 15.3 Hz, 2H), 3.78 (s, 3H), 3.60 (s, 2H), 3.35 (s, 2H), 3.27 (d, *J* = 22.5 Hz, 2H).

### Methyl 4-(4-(2-(4-(3-Fluorobenzamido)Phenyl)Quinoline-4-Carbonyl)piperazin-1-yl)Benzoate (**C26**)

Crystallization in EtOAc as a white solid (0.38 g, 53.3% yield). HRMS C_35_H_29_FN_4_O_4_ [M + H^+^] calc. 589.22064 found 589.21490. ^1^H NMR (400 MHz, DMSO) δ 10.55 (s, 1H), 8.37 (d, *J* = 8.3 Hz, 2H), 8.23–8.11 (m, 2H), 8.00 (d, *J* = 8.3 Hz, 2H), 7.86 (dd, *J* = 7.7, 4.3 Hz, 3H), 7.81 (s, 1H), 7.79 (s, 1H), 7.69–7.57 (m, 2H), 7.48 (t, *J* = 8.3 Hz, 1H), 7.00 (d, *J* = 8.5 Hz, 2H), 3.94 (s, 1H), 3.77 (s, 4H), 3.60 (s, 3H), 3.24 (s, 3H).

### Methyl 4-(4-(2-(2,5-Difluorophenyl)Quinoline-4-Carbonyl)Piperazin-1-yl)Benzoate (**C27**)

Crystallization in EtOAc as a white solid (0.28 g, 36.3% yield). HRMS C_28_H_23_F_2_N_3_O_3_ [M + H^+^] calc. 488.17410 found 488.17487. ^1^H NMR (400 MHz, DMSO) δ 8.19 (d, *J* = 8.3 Hz, 1H), 7.92 (dd, *J* = 16.9, 7.9 Hz, 4H), 7.85–7.63 (m, 4H), 7.58–7.39 (m, 2H), 6.98 (d, *J* = 8.5 Hz, 2H), 3.94 (d, *J* = 23.1 Hz, 3H), 3.77 (s, 3H), 3.58 (s, 2H), 3.17 (d, *J* = 5.5 Hz, 3H).

### Methyl 4-(4-(2-([1,1′-Biphenyl]-4-yl)Quinoline-4-Carbonyl)Piperazin-1-yl)Benzoate (**C28**)

Crystallization in EtOAc as a yellow solid (0.33 g, 40.6% yield). HRMS C_34_H_29_N_3_O_3_ [M + H^+^] calc. 528.22425 found 528.22449. ^1^H NMR (400 MHz, DMSO) δ 8.45 (d, *J* = 8.0 Hz, 2H), 8.27 (s, 1H), 8.18 (d, *J* = 8.3 Hz, 1H), 7.88 (d, *J* = 8.5 Hz, 4H), 7.83–7.76 (m, 4H), 7.67 (t, *J* = 7.5 Hz, 1H), 7.52 (t, *J* = 7.4 Hz, 2H), 7.42 (t, *J* = 7.2 Hz, 1H), 6.99 (d, *J* = 8.5 Hz, 2H), 3.97 (d, *J* = 16.6 Hz, 2H), 3.77 (s, 4H), 3.61 (d, *J* = 4.4 Hz, 2H), 3.23 (s, 3H).

### N-Hydroxy-4-(4-(2-Phenylquinoline-4-Carbonyl)Piperazin-1-yl)Benzamide (**D1**)

Methyl 4-(4-(2-phenylquinoline-4-carbonyl)piperazin-1-yl)benzoate (**C1**) (0.26 g, 0.58 mmol) and 10 ml NH_2_OK methanol solution (3.76 M) was added as solvent. After 8 h, the solvent was removed by a rotary evaporator, and 3 mol/L HCl (50 ml) was added. The solution was extracted with EtOAc (3 × 30 ml) and washed with saturated NaCl solution, dried with MgSO_4_. The desired compound (**D1**) was obtained by filtration and recrystallization in EtOAc as a white solid (0.25 g, 95.0% yield).

Mp: 213.8–214.8°C; HRMS C_27_H_24_N_4_O_3_ [M + H^+^] calc. 453.18820, found 453.19052. ^1^H NMR (400 MHz, DMSO) δ 10.96 (s, 1H), 8.80 (s, 1H), 8.34 (d, *J* = 7.6 Hz, 2H), 8.24–8.10 (m, 2H), 7.85 (t, *J* = 7.4 Hz, 2H), 7.59 (tt, *J* = 14.3, 7.8 Hz, 7H), 6.96 (d, *J* = 8.4 Hz, 2H), 3.92 (s, 1H), 3.51 (s, 2H), 3.28 (s, 2H), 3.11 (s, 1H), 2.55 (d, *J* = 15.4 Hz, 1H). ^13^C NMR (101 MHz, DMSO) δ 171.78, 166.28, 156.37, 152.76, 148.14, 143.80, 138.54, 131.05, 130.45, 130.20, 129.38, 128.61, 127.91 (d, *J* = 19.9 Hz), 125.23, 123.35, 122.88, 116.08, 114.67, 48.03, 47.61, 46.60, 45.02, 41.35.

### 4-(4-(2-(4-Bromophenyl)Quinoline-4-Carbonyl)Piperazin-1-yl)-N-Hydroxybenzamide (**D2**)

Crystallization in EtOAc as a white solid (0.13 g, 56.5% yield). Mp: 231.7–232.8°C; HRMS C_27_H_23_BrN_4_O_3_ [M + H^+^] calc. 531.09871, found 531.10126. ^1^H NMR (400 MHz, DMSO) δ 10.96 (s, 1H), 8.80 (s, 1H), 8.30 (d, *J* = 8.3 Hz, 2H), 8.22 (s, 1H), 8.16 (d, *J* = 8.7 Hz, 1H), 7.86 (d, *J* = 8.3 Hz, 2H), 7.77 (d, *J* = 8.2 Hz, 2H), 7.66 (dd, *J* = 15.4, 8.1 Hz, 3H), 6.96 (d, *J* = 8.5 Hz, 2H), 4.01–3.85 (m, 2H), 3.50 (s, 2H), 3.27 (s, 2H), 3.11 (s, 1H), 2.69 (dd, *J* = 41.1, 15.7 Hz, 1H). ^13^C NMR (101 MHz, DMSO) δ 166.17, 155.23, 152.76, 148.08, 144.00, 137.70, 132.34, 131.20, 130.19, 129.83, 128.60, 128.24, 125.26, 124.24, 123.45, 122.89, 115.91, 114.68, 48.03, 47.61, 46.58, 41.36.

### 4-(4-(2-(4-Chlorophenyl)Quinoline-4-Carbonyl)Piperazin-1-yl)-N-Hydroxybenzamide (**D3**)

Crystallization in EtOAc as a white solid (0.17 g, 68.1% yield). Mp: 228.1–229.2°C; HRMS C_27_H_23_ClN_4_O_3_ [M + H^+^] calc. 487.14922, found 487.15167. ^1^H NMR (400 MHz, DMSO) δ 10.95 (s, 1H), 8.80 (s, 1H), 8.37 (d, *J* = 8.2 Hz, 2H), 8.23 (s, 1H), 8.16 (d, *J* = 8.6 Hz, 1H), 7.87 (d, *J* = 8.3 Hz, 2H), 7.72–7.58 (m, 6H), 6.96 (d, *J* = 8.6 Hz, 2H), 4.01–3.86 (m, 2H), 3.50 (s, 2H), 3.27 (s, 2H), 3.12 (d, *J* = 7.4 Hz, 1H). ^13^C NMR (101 MHz, DMSO) δ 166.18, 155.14, 152.76, 148.07, 143.99, 137.34, 135.38, 131.19, 130.19, 129.49 (d, *J* = 16.2 Hz), 128.60, 128.22, 125.25, 123.42, 122.89, 115.96, 114.68, 60.23, 48.03, 47.62, 46.59, 41.36.

### 4-(4-(2-(4-Fluorophenyl)Quinoline-4-Carbonyl)Piperazin-1-yl)-N-Hydroxybenzamide (**D4**)

Crystallization in EtOAc as a white solid (0.12 g, 54.5% yield). Mp: 163.5–164.8°C; HRMS C_27_H_23_FN_4_O_3_ [M + H^+^] calc. 470.17542, found 471.18057. ^1^H NMR (400 MHz, DMSO) δ 10.96 (s, 1H), 8.80 (s, 1H), 8.45–8.34 (m, 1H), 8.29 (d, *J* = 8.5 Hz, 1H), 8.22–8.04 (m, 2H), 7.84 (dd, *J* = 14.6, 9.9 Hz, 2H), 7.72–7.55 (m, 3H), 7.40 (t, *J* = 8.7 Hz, 1H), 7.09 (d, *J* = 8.5 Hz, 1H), 6.96 (d, *J* = 8.7 Hz, 2H), 3.92 (s, 2H), 3.50 (s, 2H), 3.19 (d, *J* = 64.9 Hz, 4H). ^13^C NMR (101 MHz, DMSO) δ 166.23, 155.33, 152.75, 148.07, 143.90, 131.11, 130.16, 129.29, 128.58, 128.03, 125.23, 123.26, 116.38, 116.17, 115.92, 115.15, 114.68, 63.75, 48.04, 47.62, 46.59, 41.35.

### N-Hydroxy-4-(4-(2-(4-(Methylthio)Phenyl)Quinoline-4-Carbonyl)Piperazin-1-yl)Benzamide (**D5**)

Crystallization in EtOAc as a white solid (0.11 g, 73.3% yield). Mp: 177.2–179.6°C; HRMS C_28_H_26_N_4_O_3_S [M + H^+^] calc. 499.17592, found 499.17838. ^1^H NMR (400 MHz, DMSO) δ 10.95 (s, 1H), 8.80 (s, 1H), 8.30 (d, *J* = 8.0 Hz, 2H), 8.20–8.09 (m, 2H), 7.84 (d, *J* = 8.8 Hz, 2H), 7.65 (d, *J* = 8.2 Hz, 3H), 7.43 (d, *J* = 8.0 Hz, 2H), 6.96 (d, *J* = 8.4 Hz, 2H), 3.91 (s, 1H), 3.51 (s, 2H), 3.28–3.18 (m, 3H), 3.11 (s, 2H), 2.56 (s, 3H). ^13^C NMR (101 MHz, DMSO) δ 166.30, 155.80, 152.77, 148.14, 143.75, 141.47, 134.84, 131.03, 130.08, 128.60, 128.16, 127.82, 126.20, 125.20, 123.26, 122.89, 115.73, 114.67, 60.23, 48.03, 47.61, 46.59, 41.35, 21.24, 14.78, 14.56.

### N-Hydroxy-4-(4-(2-(4-Methoxyphenyl)Quinoline-4-Carbonyl)Piperazin-1-yl)Benzamide (**D6**)

Crystallization in EtOAc as a white solid (0.11 g, 43.9% yield). Mp: 184.3–186.8°C; HRMS C_28_H_26_N_4_O_4_ [M + H^+^] calc. 483.19876, found 483.20083. ^1^H NMR (400 MHz, DMSO) δ 10.95 (s, 1H), 8.80 (s, 1H), 8.31 (d, *J* = 8.5 Hz, 2H), 8.18–8.05 (m, 2H), 7.82 (t, *J* = 6.5 Hz, 2H), 7.63 (dd, *J* = 15.7, 8.0 Hz, 3H), 7.11 (d, *J* = 8.4 Hz, 2H), 6.96 (d, *J* = 8.6 Hz, 2H), 3.99 (d, *J* = 8.2 Hz, 1H), 3.91 (s, 1H), 3.86 (s, 3H), 3.50 (s, 2H), 3.27 (s, 3H), 3.11 (s, 1H). ^13^C NMR (101 MHz, DMSO) δ 166.37, 161.39, 156.03, 152.77, 148.14, 143.60, 130.95 (d, *J* = 6.6 Hz), 129.97, 129.29, 128.60, 127.52, 125.17, 122.94 (d, *J* = 12.4 Hz), 115.59, 114.71 (d, *J* = 7.4 Hz), 60.23, 55.82, 48.03, 47.62, 46.59, 41.33.

### N-Hydroxy-4-(4-(2-(4-(Trifluoromethyl)Phenyl)Quinoline-4-Carbonyl)Piperazin-1-yl)Benzamide (**D7**)

Crystallization in EtOAc as a white solid (0.09 g, 90.0% yield). Mp: 212.7–213.9°C; HRMS C_28_H_23_F_3_N_4_O_3_ [M + H^+^] calc. 521.17558, found 521.17804. ^1^H NMR (400 MHz, DMSO) δ 10.96 (s, 1H), 8.80 (s, 1H), 8.56 (d, *J* = 8.1 Hz, 2H), 8.31 (s, 1H), 8.20 (d, *J* = 8.7 Hz, 1H), 7.96–7.86 (m, 4H), 7.74–7.62 (m, 3H), 6.96 (d, *J* = 8.5 Hz, 2H), 3.96 (d, *J* = 30.8 Hz, 2H), 3.51 (s, 2H), 3.31–3.05 (m, 4H). ^13^C NMR (101 MHz, DMSO) δ 166.12, 154.87, 152.76, 148.09, 144.14, 142.33, 131.34, 130.35, 128.59, 126.28, 125.28, 123.66, 122.87, 116.37, 114.69, 60.25, 48.03, 47.61, 46.59, 41.38.

### 4-(4-(2-(3-Chlorophenyl)Quinoline-4-Carbonyl)Piperazin-1-yl)-N-Hydroxybenzamide (**D8**)

Crystallization in EtOAc as a white solid (0.06 g, 40.0% yield). Mp: 207.1–208.9°C; HRMS C_27_H_23_ClN_4_O_3_ [M + H^+^] calc. 487.14922, found 487.15164. ^1^H NMR (400 MHz, DMSO) δ 10.95 (s, 1H), 8.80 (s, 1H), 8.40 (s, 1H), 8.30 (d, *J* = 18.8 Hz, 2H), 8.19 (d, *J* = 8.7 Hz, 1H), 7.91–7.82 (m, 2H), 7.73–7.56 (m, 6H), 6.96 (d, *J* = 8.4 Hz, 2H), 3.92 (s, 1H), 3.51 (s, 2H), 3.28 (s, 3H), 3.11 (s, 1H). _13_C NMR (101 MHz, DMSO) δ 166.13, 154.78, 152.75, 148.05, 144.07, 140.62, 134.39, 131.29, 130.25 (d, *J* = 7.0 Hz), 128.60, 128.39, 127.41, 126.43, 125.25, 123.60, 122.88, 116.14, 114.67, 48.02, 47.60, 46.58, 41.35.

### N-Hydroxy-4-(4-(2-(m-Tolyl)Quinoline-4-Carbonyl)Piperazin-1-yl)Benzamide (**D9**)

Crystallization in EtOAc as a white solid (0.08 g, 53.3% yield). Mp: 199.2–199.7°C; HRMS C_28_H_26_N_4_O_3_ [M + H^+^] calc. 467.20385, found 467.20642. ^1^H NMR (400 MHz, DMSO) δ 10.96 (s, 1H), 8.80 (s, 1H), 8.14 (dd, *J* = 15.5, 9.3 Hz, 4H), 7.85 (t, *J* = 7.3 Hz, 2H), 7.65 (t, *J* = 8.2 Hz, 3H), 7.46 (t, *J* = 7.7 Hz, 1H), 7.35 (d, *J* = 7.2 Hz, 1H), 6.96 (d, *J* = 8.8 Hz, 2H), 3.90 (s, 1H), 3.51 (s, 2H), 3.27 (s, 3H), 3.11 (s, 2H), 2.45 (s, 3H). ^13^C NMR (101 MHz, DMSO) δ 166.30, 156.48, 152.75, 148.13, 143.74, 138.55 (d, *J* = 10.5 Hz), 131.05 (d, *J* = 7.2 Hz), 130.18, 129.28, 128.60, 128.32, 127.94, 125.10 (d, *J* = 19.6 Hz), 123.32, 122.87, 116.13, 114.67, 48.04, 47.60, 46.59, 41.33, 21.60.

### 4-(4-(2-(2-Fluorophenyl)Quinoline-4-Carbonyl)Piperazin-1-yl)-N-Hydroxybenzamide (**D10**)

Crystallization in EtOAc as a white solid (0.08 g, 80.0% yield). Mp: 172.5–173.3°C; HRMS C_27_H_23_FN_4_O_3_ [M + H^+^] calc. 471.17877, found 471.18130. ^1^H NMR (400 MHz, DMSO) δ 10.95 (s, 1H), 8.80 (s, 1H), 8.18 (d, *J* = 8.4 Hz, 1H), 8.08 (t, *J* = 7.9 Hz, 1H), 7.89 (d, *J* = 14.4 Hz, 3H), 7.77–7.52 (m, 4H), 7.42 (dd, *J* = 14.0, 7.1 Hz, 2H), 6.95 (d, *J* = 8.4 Hz, 2H), 3.89 (s, 2H), 3.49 (s, 2H), 3.19 (d, *J* = 64.2 Hz, 4H). ^13^C NMR (101 MHz, DMSO) δ 166.08, 161.82, 159.35, 152.76, 148.23, 143.13, 132.31, 131.81, 131.20, 130.20, 128.61, 127.30, 125.49, 125.24, 123.21, 122.82, 119.33, 117.01, 116.79, 114.69, 60.28, 48.03, 47.57, 46.58, 41.38, 14.54.

### N-Hydroxy-4-(4-(2-(3-Methoxyphenyl)Quinoline-4-Carbonyl)Piperazin-1-yl)Benzamide (**D11**)

Crystallization in EtOAc as a white solid (0.10 g, 66.7% yield). Mp: 179.8–180.9°C; HRMS C_28_H_26_N_4_O_4_ [M + H^+^] calc. 483.19876, found 483.20111. ^1^H NMR (400 MHz, DMSO) δ 10.96 (s, 1H), 8.80 (s, 1H), 8.24–8.11 (m, 2H), 7.99–7.77 (m, 4H), 7.66 (t, *J* = 9.2 Hz, 3H), 7.48 (t, *J* = 8.0 Hz, 1H), 7.11 (d, *J* = 8.0 Hz, 1H), 6.96 (d, *J* = 8.4 Hz, 2H), 3.89 (s, 4H), 3.51 (s, 3H), 3.19 (d, *J* = 67.9 Hz, 4H). ^13^C NMR (101 MHz, DMSO) δ 166.27, 164.73, 160.29, 156.14, 152.75, 148.06, 143.77, 140.02, 131.04, 130.47, 130.23, 128.60, 128.05, 125.20, 123.43, 122.88, 120.22, 116.24, 114.67, 112.94, 55.80, 48.02, 47.60, 46.59, 41.35.

### 4-(4-(2-(3,5-Difluorophenyl)Quinoline-4-Carbonyl)Piperazin-1-yl)-N-Hydroxybenzamide (**D12**)

Crystallization in EtOAc as a white solid (0.09 g, 60.0% yield). Mp: 221.8–222.0°C; HRMS C_28_H_26_N_4_O_4_ [M + H^+^] calc. 489.16935, found 489.17206. ^1^H NMR (400 MHz, DMSO) δ 10.96 (s, 1H), 8.80 (s, 1H), 8.31 (s, 1H), 8.19 (d, *J* = 8.6 Hz, 1H), 8.10 (d, *J* = 7.7 Hz, 2H), 7.94–7.60 (m, 6H), 7.44 (t, *J* = 8.9 Hz, 1H), 6.96 (d, *J* = 8.6 Hz, 2H), 3.95 (d, *J* = 18.2 Hz, 2H), 3.51 (s, 2H), 3.20 (d, *J* = 62.7 Hz, 3H). ^13^C NMR (101 MHz, DMSO) δ 166.03, 162.15, 152.75, 147.90, 144.21, 131.36, 130.36, 128.65 (d, *J* = 8.0 Hz), 125.28, 123.82, 122.91, 116.13, 114.68, 111.12–110.93 (m), 110.79 (d, *J* = 26.8 Hz), 105.74, 48.01, 47.62, 46.58, 41.38. ^13^C NMR (101 MHz, DMSO) δ 166.03, 162.15, 152.75, 147.90, 144.21, 131.36, 130.36, 128.65 (d, *J* = 8.0 Hz), 125.28, 123.82, 122.91, 116.13, 114.68, 111.12–110.93 (m), 110.79 (d, *J* = 26.8 Hz), 105.74, 48.01, 47.62, 46.58, 41.38.

### N-Hydroxy-4-(4-(2-(2-Methoxyphenyl)Quinoline-4-Carbonyl)Piperazin-1-yl)Benzamide (**D13**)

Crystallization in EtOAc as a white solid (0.11 g, 73.3% yield). Mp: 224.0–224.9°C; HRMS C_28_H_26_N_4_O_4_ [M + H^+^] calc. 483.19876, found 483.20151. ^1^H NMR (400 MHz, DMSO) δ 10.96 (s, 1H), 8.81 (s, 1H), 8.13 (d, *J* = 8.4 Hz, 1H), 7.90–7.79 (m, 4H), 7.66 (t, *J* = 8.9 Hz, 3H), 7.50 (t, *J* = 7.8 Hz, 1H), 7.21 (d, *J* = 8.3 Hz, 1H), 7.14 (t, *J* = 7.4 Hz, 1H), 6.96 (d, *J* = 8.4 Hz, 2H), 3.94 (d, *J* = 21.6 Hz, 2H), 3.87 (s, 3H), 3.48 (s, 3H), 3.23 (d, *J* = 52.7 Hz, 3H). ^13^C NMR (101 MHz, DMSO) δ 166.34, 157.51, 156.51, 152.75, 148.33, 141.55, 131.49 (d, *J* = 5.0 Hz), 130.64, 130.09, 128.62, 127.91, 125.22, 122.99 (d, *J* = 17.6 Hz), 121.28, 120.48, 114.66, 112.49, 56.27, 48.10, 47.64, 46.61, 41.36.

### 4-(4-(2-(2-Chlorophenyl)Quinoline-4-Carbonyl)Piperazin-1-yl)-N-Hydroxybenzamide (**D14**)

Crystallization in EtOAc as a white solid (0.09 g, 60.0% yield). Mp: 235.0–236.4°C; HRMS C_27_H_23_ClN_4_O_3_ [M + H^+^] calc. 487.14922, found 487.15176. ^1^H NMR (400 MHz, DMSO) δ 10.95 (s, 1H), 8.80 (s, 1H), 8.15 (d, *J* = 8.4 Hz, 1H), 7.90 (dd, *J* = 18.0, 8.2 Hz, 2H), 7.80 (s, 1H), 7.73 (t, *J* = 7.5 Hz, 2H), 7.64 (d, *J* = 7.9 Hz, 3H), 7.58–7.51 (m, 2H), 6.95 (d, *J* = 8.6 Hz, 2H), 3.90 (s, 1H), 3.48 (s, 3H), 3.32–3.21 (m, 3H), 3.15 (s, 1H). ^13^C NMR (101 MHz, DMSO) δ 165.93, 157.01, 152.75, 148.09, 142.42, 139.13, 132.30, 131.82, 131.13 (d, *J* = 9.9 Hz), 130.40, 130.15, 128.58, 128.01, 125.39, 123.25, 122.89, 119.91, 114.69, 48.07, 47.59, 46.62, 41.39.

### 4-(4-(2-(3-Fluorophenyl)Quinoline-4-Carbonyl)Piperazin-1-yl)-N-Hydroxybenzamide (**D15**)

Crystallization in EtOAc as a white solid (0.06 g, 40.0% yield). Mp: 225.0–226.3°C; HRMS C_27_H_23_FN_4_O_3_ [M + H^+^] calc. 471.17877, found 471.18146. ^1^H NMR (400 MHz, DMSO) δ 10.96 (s, 1H), 8.80 (s, 1H), 8.29–8.13 (m, 4H), 7.92–7.84 (m, 2H), 7.74–7.57 (m, 4H), 7.38 (t, *J* = 8.0 Hz, 1H), 6.96 (d, *J* = 8.6 Hz, 2H), 3.95 (d, *J* = 21.9 Hz, 2H), 3.51 (s, 2H), 3.20 (d, *J* = 65.1 Hz, 4H). ^13^C NMR (101 MHz, DMSO) δ 166.15, 154.94, 152.76, 148.03, 144.02, 141.07, 131.29 (d, *J* = 16.5 Hz), 130.28, 128.60, 128.35, 125.25, 123.85, 123.59, 122.89, 117.34, 116.14, 114.68, 114.44, 48.01, 47.61, 46.59, 41.37.

### N-Hydroxy-4-(4-(2-(o-Tolyl)Quinoline-4-Carbonyl)Piperazin-1-yl)Benzamide (**D16**)

Crystallization in EtOAc as a white solid (0.07 g, 46.7% yield). Mp: 223.4–223.9°C; HRMS C_28_H_26_N_4_O_3_ [M + H^+^] calc. 467.20385, found 467.20605. ^1^H NMR (400 MHz, DMSO) δ 10.95 (s, 1H), 8.80 (s, 1H), 8.12 (d, *J* = 8.3 Hz, 1H), 7.87 (dd, *J* = 16.8, 8.3 Hz, 2H), 7.75–7.60 (m, 4H), 7.56 (d, *J* = 7.0 Hz, 1H), 7.39 (d, *J* = 7.9 Hz, 3H), 6.95 (d, *J* = 8.3 Hz, 2H), 3.86 (s, 1H), 3.49 (s, 4H), 3.13 (s, 2H), 2.43 (s, 4H). ^13^C NMR (101 MHz, DMSO) δ 166.18, 159.67, 152.77, 147.88, 142.82, 140.03, 136.23, 131.31, 130.95, 130.35, 130.03, 129.26, 128.61, 128.11, 126.49, 125.22, 122.79, 119.48, 114.69, 60.28, 48.06, 47.56, 46.60, 41.36, 20.66, 14.54.

### 4-(4-(2-(4-Fluoro-3-Methylphenyl)Quinoline-4-Carbonyl)Piperazin-1-yl)-N-Hydroxybenzamide (**D17**)

Crystallization in EtOAc as a white solid (0.16 g, 53.3% yield). Mp: 176.6–186.2°C; HRMS C_28_H_25_FN_4_O_3_ [M + H^+^] calc. 485.19442, found 485.19553. ^1^H NMR (400 MHz, DMSO) δ 10.96 (s, 1H), 8.81 (s, 1H), 8.30 (d, *J* = 7.4 Hz, 1H), 8.25–8.09 (m, 3H), 7.93–7.79 (m, 2H), 7.65 (d, *J* = 8.3 Hz, 3H), 7.32 (t, *J* = 9.1 Hz, 1H), 6.96 (d, *J* = 8.4 Hz, 2H), 3.91 (s, 1H), 3.51 (s, 3H), 3.32–3.02 (m, 4H), 2.42–2.24 (m, 3H). ^13^C NMR (101 MHz, DMSO) δ 166.26, 163.70, 161.25, 155.47, 152.75, 148.07, 143.83, 134.67, 131.16 (d, *J* = 17.0 Hz), 130.11, 128.61, 127.96, 127.45, 125.28 (d, *J* = 15.1 Hz), 123.21, 122.89, 115.91, 114.67, 48.04, 47.60, 46.59, 41.33.

### 4-(4-(2-(4-(Dimethylamino)Phenyl)Quinoline-4-Carbonyl)Piperazin-1-yl)-N-Hydroxybenzamide (**D18**)

Crystallization in EtOAc as a red solid (0.05 g, 16.7% yield). Mp: 200.6–201.2°C; HRMS C_29_H_29_N_5_O_3_ [M + H^+^] calc. 496.23039, found 496.23093. ^1^H NMR (400 MHz, DMSO) δ 10.95 (s, 1H), 8.80 (s, 1H), 8.20 (d, *J* = 8.5 Hz, 2H), 8.04 (d, *J* = 7.7 Hz, 2H), 7.80–7.67 (m, 2H), 7.65 (d, *J* = 8.4 Hz, 2H), 7.55 (t, *J* = 7.4 Hz, 1H), 6.95 (d, *J* = 8.4 Hz, 2H), 6.84 (d, *J* = 8.6 Hz, 2H), 3.94 (d, *J* = 32.6 Hz, 2H), 3.50 (s, 2H), 3.27 (s, 3H), 3.02 (s, 6H), -1.66 – -1.87 (m, 1H). ^13^C NMR (101 MHz, DMSO) δ 166.55, 156.52, 152.78, 151.97, 148.27, 143.20, 130.71, 128.68 (d, *J* = 16.6 Hz), 126.81, 125.70, 125.09, 122.78 (d, *J* = 19.0 Hz), 115.15, 114.66, 112.33, 47.61, 46.60, 41.31.

### 4-(4-(2-(4-Benzamidophenyl)Quinoline-4-Carbonyl)Piperazin-1-yl)-N-Hydroxybenzamide (**D19**)

Crystallization in EtOAc as a yellow solid (0.07 g, 46.7% yield). Mp: 173.9–174.8°C; HRMS C_34_H_29_N_5_O_4_ [M + H^+^] calc. 572.22531, found 572.22815. ^1^H NMR (400 MHz, DMSO) δ 10.96 (s, 1H), 10.48 (s, 1H), 8.80 (s, 1H), 8.37 (d, *J* = 8.4 Hz, 2H), 8.19 (s, 1H), 8.14 (d, *J* = 8.6 Hz, 1H), 8.05–7.97 (m, 4H), 7.85 (d, *J* = 7.9 Hz, 2H), 7.69–7.52 (m, 6H), 6.96 (d, *J* = 8.7 Hz, 2H), 3.93 (s, 1H), 3.51 (s, 4H), 3.13 (s, 1H), 2.67 (s, 2H). ^13^C NMR (101 MHz, DMSO) δ 207.54–131.11 (m), 207.54–128.98 (m), 207.54–129.02 (m), 207.54–128.67 (m), 207.54–127.84 (m), 207.54–125.39 (m), 207.54–123.27 (m), 218.32–122.78 (m), 120.69, 115.75, 114.68, 47.63.

### 4-Fluoro-N-(4-(4-(4-(4-(Hydroxycarbamoyl)Phenyl)Piperazine-1-Carbonyl)Quinolin-2-yl)Phenyl)Benzamide (**D20**)

Crystallization in EtOAc as a yellow solid (0.07 g, 46.7% yield). Mp: 178.3–180.3°C; HRMS C_34_H_28_FN_5_O_4_ [M + H^+^] calc. 590.21589, found 590.21875. ^1^H NMR (400 MHz, DMSO) δ 10.96 (s, 1H), 10.49 (s, 1H), 8.80 (s, 1H), 8.35 (t, J = 9.7 Hz, 2H), 8.19 (s, 1H), 8.14 (d, J = 8.8 Hz, 1H), 8.10 (s, 1H), 8.08 (d, J = 5.7 Hz, 1H), 7.99 (d, J = 8.4 Hz, 2H), 7.90 (d, J = 8.3 Hz, 1H), 7.84 (d, J = 8.2 Hz, 2H), 7.65 (d, J = 8.3 Hz, 3H), 7.40 (t, J = 8.6 Hz, 1H), 6.96 (d, J = 8.5 Hz, 2H), 3.93 (s, 1H), 3.51 (s, 2H), 3.29–3.20 (m, 2H), 3.12 (s, 1H), 2.69 (dd, J = 41.1, 15.4 Hz, 2H). ^13^C NMR (101 MHz, DMSO) δ 171.76, 165.09, 152.78, 131.04, 128.61, 128.26, 122.88, 120.73, 115.99, 115.77, 114.68, 72.87, 43.21.

### 3-Bromo-N-(4-(4-(4-(4-(Hydroxycarbamoyl)Phenyl)Piperazine-1-Carbonyl)Quinolin-2-yl)Phenyl)Benzamide (**D21**)

Crystallization in EtOAc as a yellow solid (0.12 g, 80.0% yield). Mp: 170.5–170.8°C; HRMS C_34_H_28_BrN_5_O_4_ [M + H^+^] calc. 650.13582, found 650.13867. ^1^H NMR (400 MHz, DMSO) δ 10.96 (s, 1H), 10.58 (s, 1H), 8.80 (s, 1H), 8.37 (d, J = 8.5 Hz, 2H), 8.19 (s, 2H), 8.14 (d, J = 8.6 Hz, 1H), 8.00 (d, J = 8.3 Hz, 3H), 7.84 (t, J = 7.8 Hz, 3H), 7.65 (d, J = 8.1 Hz, 3H), 7.53 (t, J = 7.9 Hz, 1H), 6.96 (d, J = 8.3 Hz, 2H), 3.96 (d, J = 24.8 Hz, 2H), 3.51 (s, 2H), 3.30–3.20 (m, 2H), 3.12 (s, 1H), 2.67 (s, 1H). ^13^C NMR (101 MHz, DMSO) δ 166.34, 164.63, 155.86, 152.77, 148.16, 141.19, 137.35, 134.94, 133.81, 131.17, 130.84, 128.63, 128.25, 127.50, 123.18, 122.88, 122.19, 120.84, 115.77, 114.67.

### 2-Fluoro-N-(4-(4-(4-(4-(Hydroxycarbamoyl)Phenyl)Piperazine-1-Carbonyl)Quinolin-2-yl)Phenyl)Benzamide (**D22**)

Crystallization in EtOAc as a yellow solid (0.05 g, 33.3% yield). Mp: 163.5–164.2°C; HRMS C_34_H_28_FN_5_O_4_ [M + H^+^] calc. 589.21253, found 588.20569. ^1^H NMR (400 MHz, DMSO) δ 10.96 (s, 1H), 10.66 (s, 1H), 8.80 (s, 1H), 8.36 (d, J = 8.3 Hz, 2H), 8.18 (s, 1H), 8.14 (d, J = 8.8 Hz, 1H), 7.93 (d, J = 8.5 Hz, 2H), 7.88–7.81 (m, 2H), 7.72 (t, J = 7.5 Hz, 1H), 7.66 (s, 1H), 7.64 (s, 2H), 7.61 (d, J = 7.8 Hz, 1H), 7.43–7.33 (m, 2H), 6.96 (d, J = 8.6 Hz, 2H), 3.92 (s, 1H), 3.51 (s, 2H), 3.30–3.21 (m, 2H), 3.12 (s, 1H), 2.79–2.57 (m, 2H). ^13^C NMR (101 MHz, DMSO) δ 171.76, 166.34, 163.46, 155.82, 152.77, 148.16, 143.72, 141.06, 133.81, 130.42, 128.61, 128.41, 125.11, 123.18, 120.19, 116.58, 115.75, 114.68.

### 2-Chloro-N-(4-(4-(4-(4-(Hydroxycarbamoyl)Phenyl)Piperazine-1-Carbonyl)Quinolin-2-yl)Phenyl)Benzamide (**D23**)

Crystallization in EtOAc as a yellow solid (0.07 g, 46.7% yield). Mp: 167.2–167.8°C; HRMS C_34_H_28_ClN_5_O_4_ [M + H^+^] calc. 606.18634, found 606.18951. ^1^H NMR (400 MHz, DMSO) δ 10.96 (s, 1H), 10.75 (s, 1H), 8.80 (s, 1H), 8.36 (d, J = 8.4 Hz, 2H), 8.20–8.09 (m, 2H), 7.92 (d, *J* = 8.3 Hz, 2H), 7.84 (t, *J* = 6.8 Hz, 3H), 7.69–7.45 (m, 9H), 6.96 (d, *J* = 8.6 Hz, 2H), 3.93–3.80 (m, 1H), 3.51 (s, 2H), 3.12 (s, 2H). ^13^C NMR (101 MHz, DMSO) δ 152.76, 130.30 (d, *J* = 23.8 Hz), 130.16–130.08 (m), 129.47, 128.42, 127.79, 123.17, 120.06, 114.68.

### 2,4-Difluoro-N-(4-(4-(4-(4-(Hydroxycarbamoyl)Phenyl)Piperazine-1-Carbonyl)Quinolin-2-yl)Phenyl)Benzamide (**D24**)

Crystallization in EtOAc as a red solid (0.03 g, 42.9% yield). Mp: 175.3–176.3°C; HRMS C_34_H_27_F_2_N_5_O_4_ [M + H^+^] calc. 608.20647, found 608.20923. ^1^H NMR (400 MHz, DMSO) δ 10.95 (s, 1H), 10.52 (d, *J* = 8.4 Hz, 1H), 8.80 (s, 1H), 8.38 (d, *J* = 8.3 Hz, 2H), 8.21–8.10 (m, 2H), 8.10–7.96 (m, 2H), 7.92 (d, *J* = 8.5 Hz, 2H), 7.85 (d, *J* = 8.8 Hz, 2H), 7.65 (d, *J* = 8.1 Hz, 3H), 6.96 (d, *J* = 8.4 Hz, 2H), 6.85 (dd, *J* = 16.0, 8.9 Hz, 2H), 6.68 (d, *J* = 8.4 Hz, 1H), 3.94 (s, 1H), 3.51 (s, 4H), 3.13 (s, 1H). ^13^C NMR (101 MHz, DMSO) δ 175.03, 171.76, 166.33, 155.80, 152.77, 148.16, 143.74, 140.85, 134.05, 131.03, 128.61, 128.32, 127.79, 123.20, 122.88, 120.87, 114.68, 111.85, 111.59, 72.90, 48.05, 43.16.

### 3,5-Difluoro-N-(4-(4-(4-(4-(Hydroxycarbamoyl)Phenyl)Piperazine-1-Carbonyl)Quinolin-2-yl)Phenyl)Benzamide (**D25**)

Crystallization in EtOAc as a yellow solid (0.11 g, 43.9% yield). Mp: 171.8–172.2°C; HRMS C_34_H_27_F_2_N_5_O4 [M + H^+^] calc. 608.20647, found 608.20923. 1H NMR (400 MHz, DMSO) δ 10.96 (s, 1H), 10.58 (s, 1H), 8.80 (s, 1H), 8.38 (d, J = 8.4 Hz, 2H), 8.20 (s, 1H), 8.14 (d, J = 8.7 Hz, 1H), 7.99 (d, J = 8.4 Hz, 2H), 7.84 (t, J = 7.0 Hz, 2H), 7.74 (d, J = 6.7 Hz, 2H), 7.64 (t, J = 7.5 Hz, 3H), 7.56 (t, J = 9.0 Hz, 1H), 6.96 (d, J = 8.5 Hz, 2H), 3.51 (s, 2H), 3.30–3.20 (m, 2H), 3.13 (s, 1H), 2.71 (dd, J = 41.4, 15.4 Hz, 3H). ^13^C NMR (101 MHz, DMSO) δ 175.03, 171.76, 166.33, 155.80, 152.77, 148.16, 143.74, 140.85, 134.05, 131.03, 128.61, 128.32, 127.79, 123.20, 122.88, 120.87, 114.68, 111.85, 111.59, 72.90, 48.05, 43.16.

### 3-Fluoro-N-(4-(4-(4-(4-(Hydroxycarbamoyl)Phenyl)Piperazine-1-Carbonyl)Quinolin-2-yl)Phenyl)Benzamide (**D26**)

Crystallization in EtOAc as a yellow solid (0.08 g, 32.0% yield). Mp: 232.6–232.9°C; HRMS C_34_H_28_FN_5_O4 [M + H^+^] calc. 590.21589, found 590.21637. 1H NMR (400 MHz, DMSO) δ 10.96 (s, 1H), 10.54 (s, 1H), 8.80 (s, 1H), 8.38 (d, J = 8.2 Hz, 2H), 8.23–8.07 (m, 2H), 8.00 (d, *J* = 8.3 Hz, 2H), 7.93–7.75 (m, 4H), 7.72–7.40 (m, 5H), 6.96 (d, *J* = 8.3 Hz, 2H), 3.96 (d, *J* = 25.2 Hz, 2H), 3.51 (s, 3H), 3.21 (d, *J* = 65.5 Hz, 3H). ^13^C NMR (101 MHz, DMSO) δ 175.08, 166.36, 155.92, 148.15, 143.66, 141.73, 132.85, 130.94, 130.01, 128.31, 125.34, 123.13, 120.18, 119.48, 116.39, 115.61, 47.18, 45.42, 29.60, 25.71.

### 4-(4-(2-(2,5-Difluorophenyl)Quinoline-4-Carbonyl)Piperazin-1-yl)-N-Hydroxybenzamide (**D27**)

Crystallization in EtOAc as a white solid (0.15 g, 53.6% yield). Mp: 196.9–198.0°C; HRMS C_27_H_22_F_2_N_4_O_3_ [M + H^+^] calc. 488.16600, found 489.17139. ^1^H NMR (400 MHz, DMSO) δ 10.96 (s, 1H), 8.81 (s, 1H), 8.20 (d, *J* = 8.3 Hz, 1H), 7.91 (dd, *J* = 15.9, 7.5 Hz, 4H), 7.73 (t, *J* = 7.6 Hz, 1H), 7.65 (d, *J* = 8.5 Hz, 2H), 7.56–7.38 (m, 2H), 6.95 (d, *J* = 8.5 Hz, 2H), 3.94 (d, *J* = 30.4 Hz, 2H), 3.49 (s, 2H), 3.20 (d, *J* = 56.1 Hz, 4H). ^13^C NMR (101 MHz, DMSO) δ 165.97, 152.76, 148.12, 143.40, 131.36, 130.28, 129.06–128.94 (m), 128.73 (d, *J* = 25.7 Hz), 125.26, 123.40, 122.82, 119.08, 117.67, 114.70, 48.02, 47.56, 46.57, 41.39.

### 4-(4-(2-([1,1′-Biphenyl]-4-yl)Quinoline-4-Carbonyl)Piperazin-1-yl)-N-Hydroxybenzamide (**D28**)

Crystallization in EtOAc as a white solid (0.14 g, 56.9% yield). Mp: 203.5–205.6°C; HRMS C_33_H_28_N_4_O_3_ [M + H^+^] calc. 529.21950, found 529.22034. ^1^H NMR (400 MHz, DMSO) δ 10.96 (s, 1H), 8.81 (s, 1H), 8.45 (d, J = 8.0 Hz, 2H), 8.27 (s, 1H), 8.18 (d, *J* = 8.5 Hz, 1H), 7.92–7.82 (m, 4H), 7.79 (d, *J* = 7.6 Hz, 2H), 7.67 (t, *J* = 7.9 Hz, 3H), 7.52 (t, *J* = 7.4 Hz, 2H), 7.42 (t, *J* = 7.3 Hz, 1H), 6.96 (d, *J* = 8.4 Hz, 2H), 3.97 (d, *J* = 25.3 Hz, 2H), 3.55 (d, *J* = 25.0 Hz, 2H), 3.32–3.04 (m, 3H). ^13^C NMR (101 MHz, DMSO) δ 166.30, 155.91, 152.78, 148.20, 143.80, 141.98, 139.81, 137.51, 131.09, 130.20, 129.54, 128.61, 128.40, 128.02, 127.59, 127.23, 125.23, 123.39, 122.88, 116.04, 114.68, 48.04, 47.61, 46.61, 41.38.

### 4-(4-(2-([1,1′-Biphenyl]-4-yl)Quinoline-4-Carbonyl)Piperazin-1-yl)-N′-Propylbenzohydrazide (**D29**)

Crystallization in EtOAc as a white solid (0.14 g, 35.5% yield). Mp: 223.3–224.2°C; HRMS C_36_H_35_N_5_O2 [M + H^+^] calc. 570.28243, found 570.28455. 1H NMR (400 MHz, DMSO) δ 9.83 (s, 1H), 8.50 (d, J = 7.9 Hz, 2H), 8.32 (s, 1H), 8.23 (d, *J* = 8.5 Hz, 1H), 7.98–7.88 (m, 4H), 7.84 (d, *J* = 7.5 Hz, 3H), 7.78 (d, *J* = 8.3 Hz, 2H), 7.73 (d, *J* = 7.5 Hz, 1H), 7.57 (t, *J* = 7.4 Hz, 2H), 7.48 (d, *J* = 7.2 Hz, 1H), 7.01 (d, *J* = 8.4 Hz, 2H), 3.99 (s, 1H), 3.57 (s, 2H), 3.34 (s, 1H), 3.19 (d, *J* = 8.2 Hz, 1H), 2.76 (dd, *J* = 12.9, 5.8 Hz, 2H), 1.50 (dd, *J* = 14.4, 7.2 Hz, 2H), 1.24 (dd, *J* = 17.0, 10.0 Hz, 2H), 0.99–0.86 (m, 4H). ^13^C NMR (101 MHz, DMSO) δ 166.30, 165.56, 155.91, 152.82, 148.21, 143.80, 141.98, 139.81, 137.51, 131.08, 130.20, 129.53, 128.80, 128.39, 128.00, 127.59, 127.23, 125.24, 123.39, 116.04, 114.58, 60.24, 53.74, 47.61, 46.60, 41.38, 21.34, 12.16.

### 4-(4-(2-([1,1′-Biphenyl]-4-yl)Quinoline-4-Carbonyl)Piperazin-1-yl)-N′-Butylbenzohydrazide (**D30**)

Crystallization in EtOAc as a white solid (0.14 g, 43.9% yield). Mp: 225.3–227.3°C; HRMS C_37_H_37_N_5_O_2_ [M + H^+^] calc. 584.29808, found 584.29950. ^1^H NMR (400 MHz, DMSO) δ 8.45 (d, *J* = 8.0 Hz, 2H), 8.27 (s, 1H), 8.18 (d, *J* = 8.4 Hz, 1H), 7.87 (dd, *J* = 11.7, 7.9 Hz, 4H), 7.78 (t, *J* = 8.3 Hz, 3H), 7.73 (d, *J* = 8.3 Hz, 1H), 7.70–7.62 (m, 1H), 7.52 (t, *J* = 7.4 Hz, 2H), 7.42 (t, *J* = 7.2 Hz, 1H), 7.11–6.86 (m, 2H), 6.54 (s, 1H), 3.94 (s, 1H), 3.69–3.40 (m, 3H), 3.32–3.23 (m, 2H), 3.15 (s, 1H), 2.75 (t, *J* = 6.7 Hz, 1H), 2.22 (d, *J* = 5.8 Hz, 1H), 1.70–1.21 (m, 4H), 0.90 (dt, *J* = 14.2, 7.1 Hz, 4H). ^13^C NMR (101 MHz, DMSO) δ 166.35, 163.86, 155.85, 148.18, 143.69, 141.33, 139.30, 137.73, 133.49, 132.84, 131.63, 130.90 (d, *J* = 15.8 Hz), 130.05, 128.53, 127.71, 127.07, 125.20, 124.33, 123.19, 122.81, 120.19, 119.74, 115.68, 50.90, 41.75.

### *In Vitro* HDAC Inhibitory Assay

All HDAC enzymes were purchased from BPS Bioscience. In short, HeLa cell nuclear extract solution (60 μl) was mixed with a 2.0 μM compound sample (40 μl), 60 μl of recombinant HDAC enzyme solution was mixed with various concentrations of test compound (40 μl, with concentrations from 1 nM to 100 μM), and then incubated at 37°C for 30 min (Fan et al., 2021). The reaction was terminated by adding 100 μl of imaging agent containing trypsin and trichostatin A (TSA). After standing for 20 min, the fluorescence intensity was measured at the excitation and emission wavelengths of 360 and 460 nm with a microplate reader. The inhibition rate was calculated from the fluorescence intensity readings of the test wells relative to the control wells, and the IC_50_ curve and value were determined by GraphPad Prism 6.0 software.

### *In Vitro* Antiproliferative Assay

The proliferation of cancer cells was tested by CCK-8 assay. Briefly, cells were seeded in a 96-well plate with about 5 × 10^3^ cells in each well. In the K652 cell screening, cells were treated with 2.0 μM of tested compounds. In the IC_50_ calculation, cells were treated with tested compounds (with concentrations from 0.5 to 20 μM) after 24 h of incubation. CCK-8 reagent (10 ml) was added to each well after 72 h of incubation, and cells were incubated at 37°C for 4 h. The light absorbance at 450 nm was measured by using an Opsys microplate reader (Dynex Technologies, Chantilly, VA, United States). Results are illustrated as a percent of cell viability normalized to DMSO-treated control cells.

### Cell Cycle Analysis

K562 cells were incubated with different doses of molecule **D28** and SAHA for 24 h. After treatment, cells were collected and fixed with 70% pre-cold ethanol in PBS and stored at −20°C overnight. Then, the cells were washed with PBS twice and incubated with 100 μg/ml RNase I (Solarbio, China) at 37°C for 1 h and stained with propidium iodide (PI, 10 μg/ml, Solarbio, China) for 30 min avoiding light at room temperature. Finally, DNA content was measured by flow cytometry (FACSAriaIII, Becton Dickinson, United States). The data was analyzed and fitted by ModFit software.

### Cell Apoptosis Analysis

K562 cells were treated with various concentrations of molecule **D28** and SAHA for 24 h, cells were harvested and PBS washed twice, then resuspended with binding buffer (Becton Dickinson, United States). Cells were incubated with Annexin V-BV421(Becton Dickinson, United States) and 7-AAD (Becton Dickinson, United States) double labeling for 30 min in the dark at room temperature and measured by flow cytometry (FACSAriaIII, Becton Dickinson, United States). The data was analyzed by using Flowjo-V10 software.

### Statistical Analysis

All experiments were repeated at least three times unless otherwise stated. The data were represented as mean ± SD. Statistical analyses were performed with Student’s *t*-test for two-group comparisons and using one-way ANOVA with Tukey’s *post-hoc* test for multigroup comparisons.

## Data Availability

The datasets presented in this study can be found in online repositories. The names of the repository/repositories and accession number(s) can be found in the article/Supplementary Material.
