# Supplementary material for: Discovery of 2-Phenylquinoline-4-Carboxylic Acid Derivatives as Novel Histone Deacetylase Inhibitors
Source: Front Chem. 2022 Jul 14;10:937225. doi: 10.3389/fchem.2022.937225 (PMC9333195; doi:10.3389/fchem.2022.937225)
Supplement: Supplementary file 1 [file DataSheet1.PDF]

Discovery of 2-phenylquinoline-4-carboxylic acid Derivatives as Novel Histone Deacetylase Inhibitors

Qian Hui [a†], Lihui Zhang[b†], Jinhong Feng[c] and Lei Zhang[a*]

[a] *Department of Medicinal Chemistry, School of Pharmacy, Weifang Medical University, Weifang, Shandong, China;*

[b] *School of Stomatology, Weifang Medical University, Weifang, Shandong, China;*

[c] *Shandong Analysis and Test Center, Qilu University of Technology (Shandong Academy of sciences), Jinan, Shandong, China.*

[†]*These authors have contributed equally to this work.*

Author for correspondence: Lei Zhang, Tel./fax: +86-536-8462014, E-mail: leizhangchemical@gmail.com.

Content

HRMS and ^{1}H-NMR spectrum of D1 1

^{13}C-NMR spectrum of D1 and HRMS spectrum of D2 2

^{1}H-NMR spectrum and ^{13}C-NMR of D2 3

HRMS and ^{1}H-NMR spectrum of D3 4

^{13}C-NMR spectrum of D3 and HRMS spectrum of D4 5

^{1}H-NMR spectrum and ^{13}C-NMR of D4 6

HRMS and ^{1}H-NMR spectrum of D5 7

^{13}C-NMR spectrum of D5 and HRMS spectrum of D6 8

^{1}H-NMR spectrum and ^{13}C-NMR of D6 9

HRMS and ^{1}H-NMR spectrum of D7 10

^{13}C-NMR spectrum of D7 and HRMS spectrum of D8 11

^{1}H-NMR spectrum and ^{13}C-NMR of D8 12

HRMS and ^{1}H-NMR spectrum of D9 13

^{13}C-NMR spectrum of D9 and HRMS spectrum of D10 14

^{1}H-NMR spectrum and ^{13}C-NMR of D10 15

HRMS and ^{1}H-NMR spectrum of D11 16

^{13}C-NMR spectrum of D11 and HRMS spectrum of D12 17

^{1}H-NMR spectrum and ^{13}C-NMR of D12 18

HRMS and ^{1}H-NMR spectrum of D13 19

^{13}C-NMR spectrum of D13 and HRMS spectrum of D14 20

^{1}H-NMR spectrum and ^{13}C-NMR of D14 21

HRMS and ^{1}H-NMR spectrum of D15 22

^{13}C-NMR spectrum of D15 and HRMS spectrum of D16 23

^{1}H-NMR spectrum and ^{13}C-NMR of D16 24

HRMS and ^{1}H-NMR spectrum of D17 25

^{13}C-NMR spectrum of D17 and HRMS spectrum of D18 26

^{1}H-NMR spectrum and ^{13}C-NMR of D18 27

HRMS and ^{1}H-NMR spectrum of D19 28

^{13}C-NMR spectrum of D19 and HRMS spectrum of D20 29

^{1}H-NMR spectrum and ^{13}C-NMR of D20 30

HRMS and ^{1}H-NMR spectrum of D21 31

^{13}C-NMR spectrum of D21 and HRMS spectrum of D22 32

^{1}H-NMR spectrum and ^{13}C-NMR of D22 33

HRMS and ^{1}H-NMR spectrum of D23 34

^{13}C-NMR spectrum of D23and HRMS spectrum of D24 35

^{1}H-NMR spectrum and ^{13}C-NMR of D24 36

HRMS and ^{1}H-NMR spectrum of D25 37

^{13}C-NMR spectrum of D25 and HRMS spectrum of D26 38

^{1}H-NMR spectrum and ^{13}C-NMR of D26 39

HRMS and ^{1}H-NMR spectrum of D27 40

^{13}C-NMR spectrum of D27 and HRMS spectrum of D28 41

^{1}H-NMR spectrum and ^{13}C-NMR of D28 42

HRMS and ^{1}H-NMR spectrum of D29 43

^{13}C-NMR spectrum of D29 and HRMS spectrum of D30 44

^{13}C-NMR spectrum of D30 and HRMS spectrum of D30 45

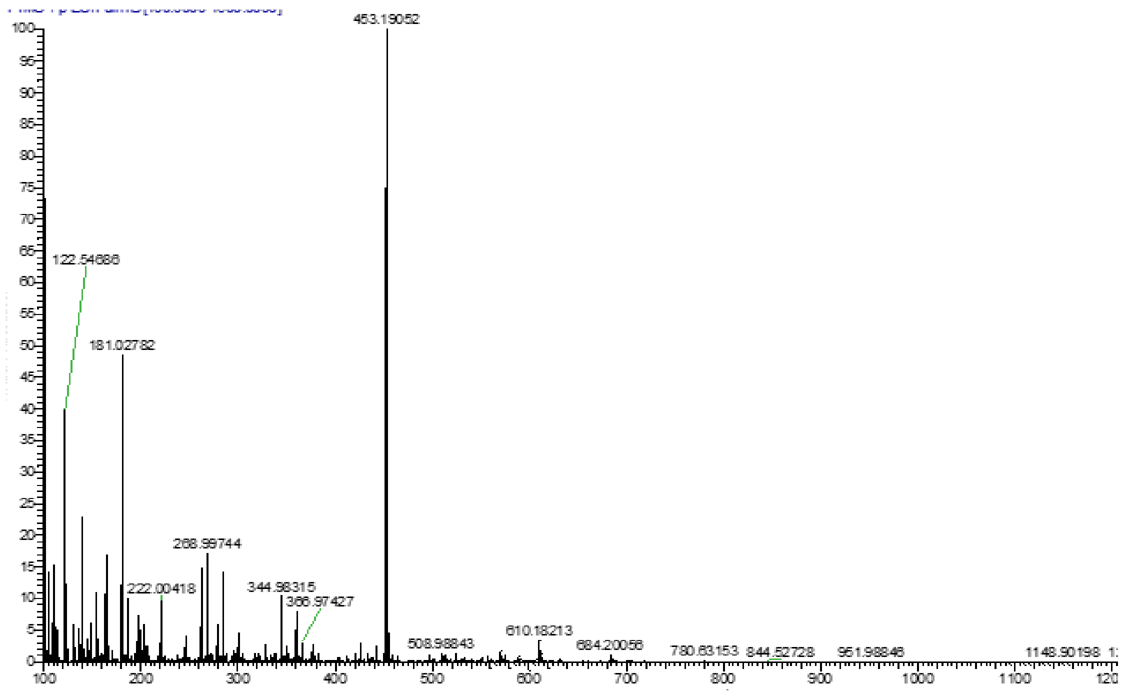

HRMS spectrum of D1

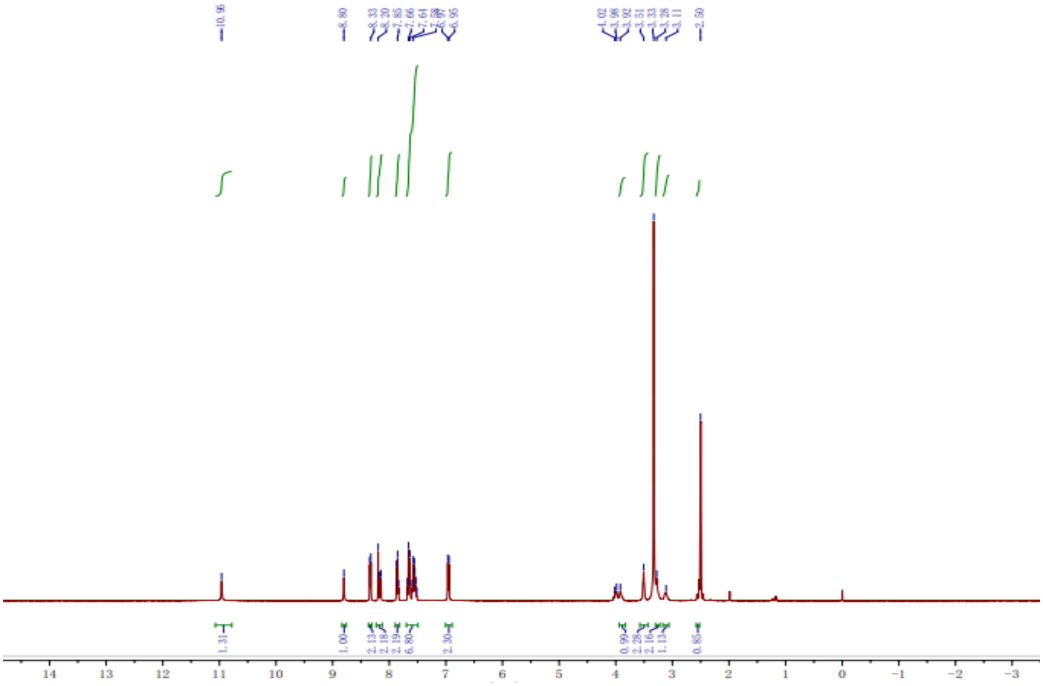

^{1}H-NMR spectrum of D1

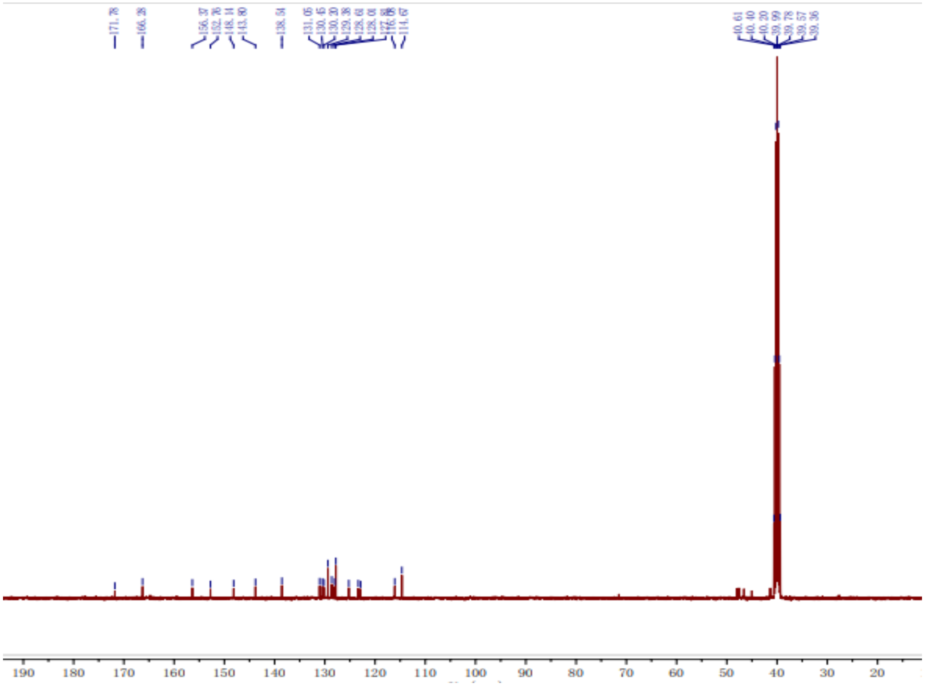

^{13}C-NMR spectrum of D1

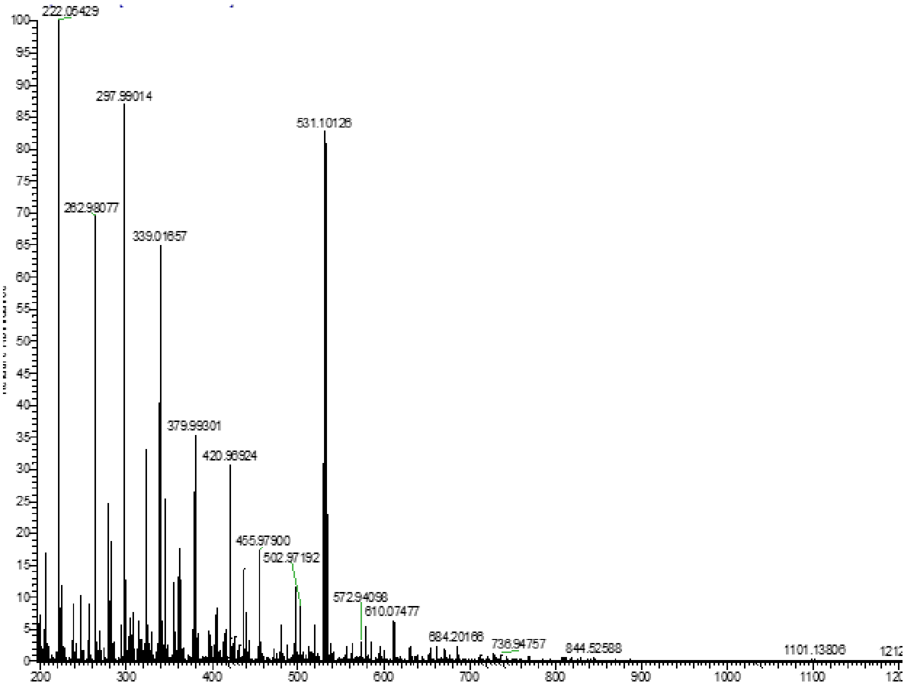

HRMS spectrum of D2

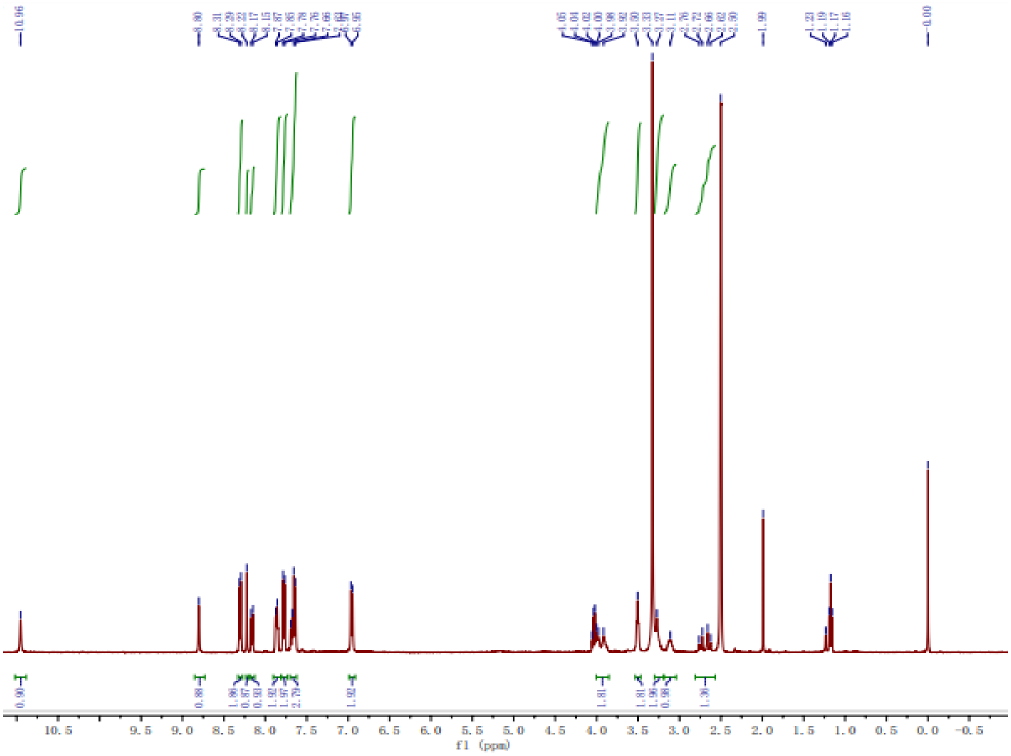

^{1}H-NMR spectrum of D2

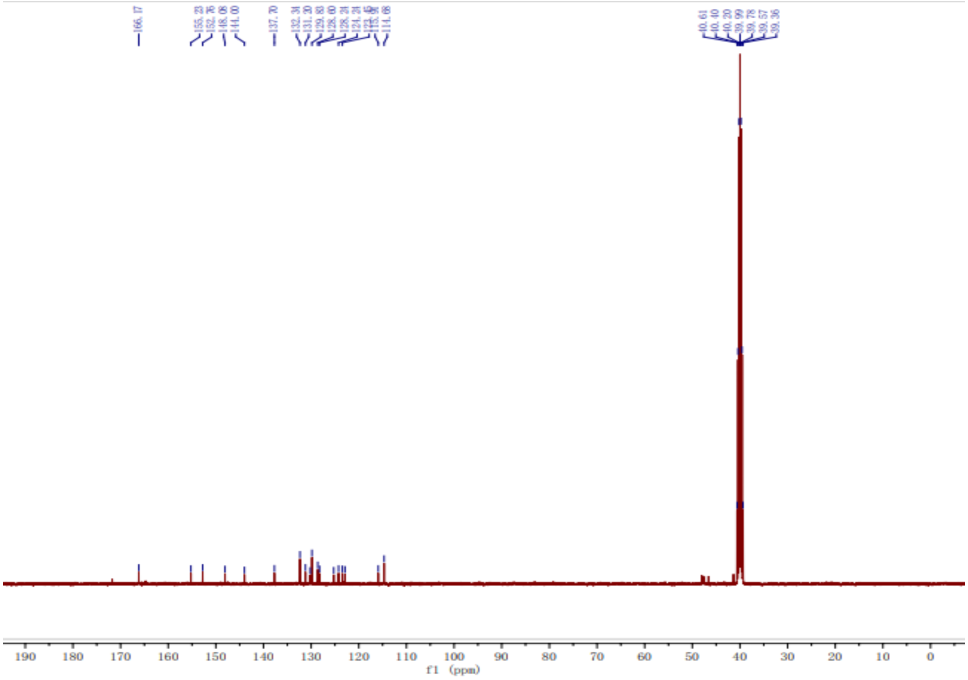

^{13}C-NMR spectrum of D2

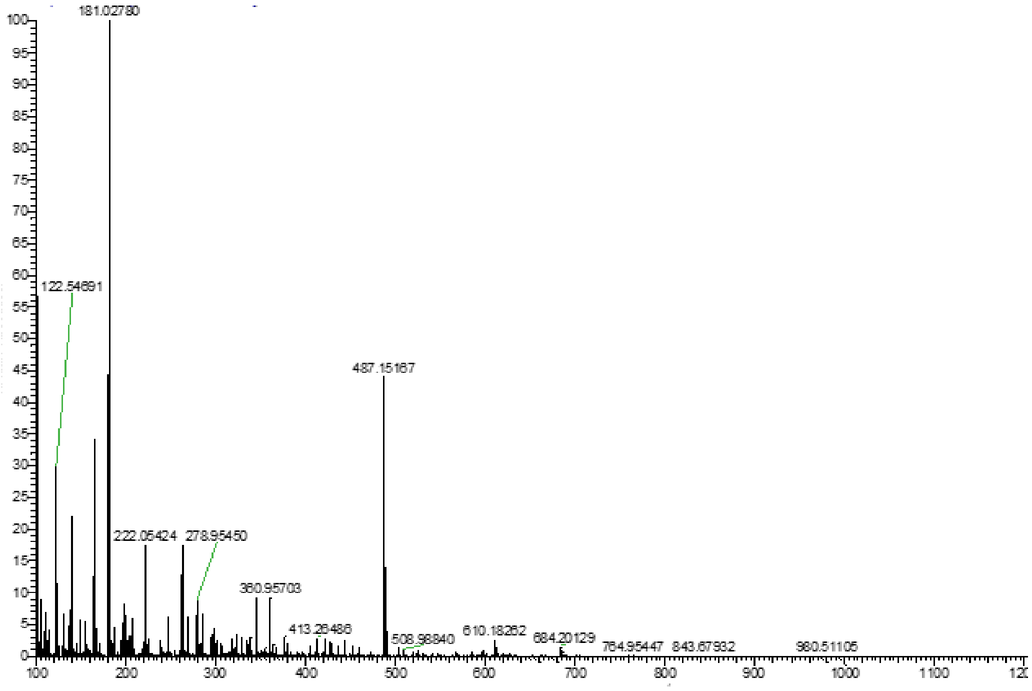

HRMS spectrum of D3

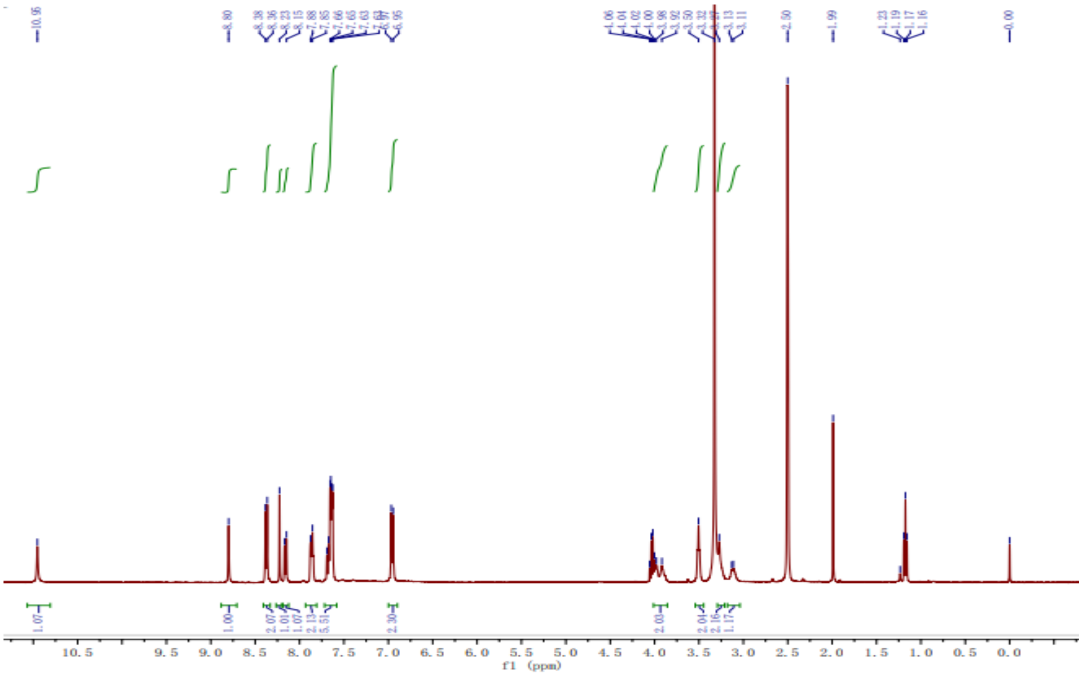

^{1}H-NMR spectrum of D3

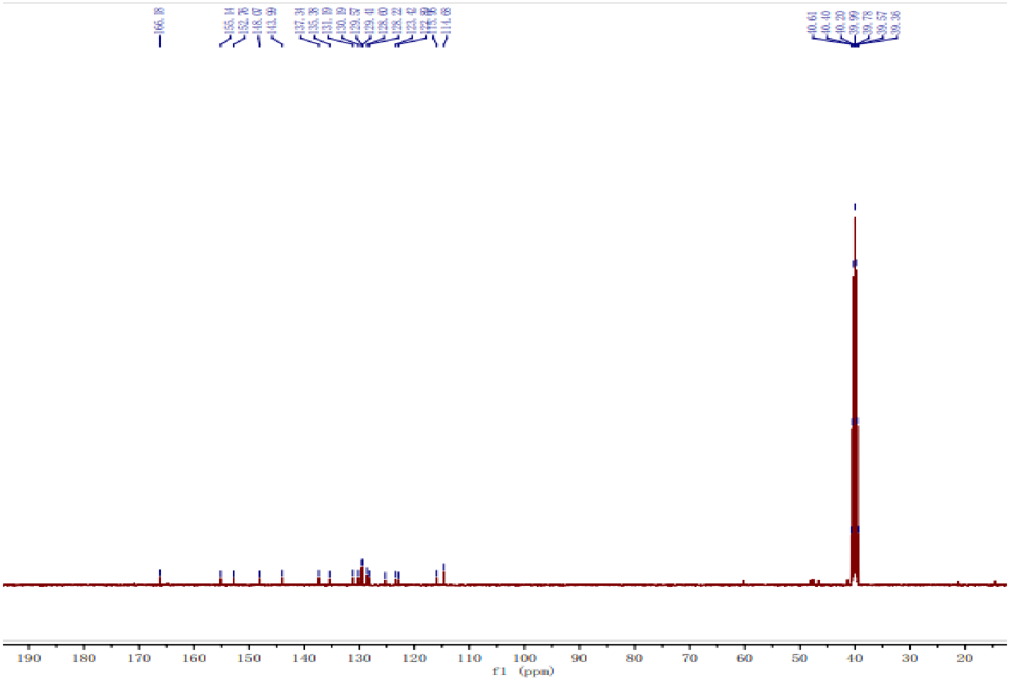

^{13}C-NMR spectrum of D3

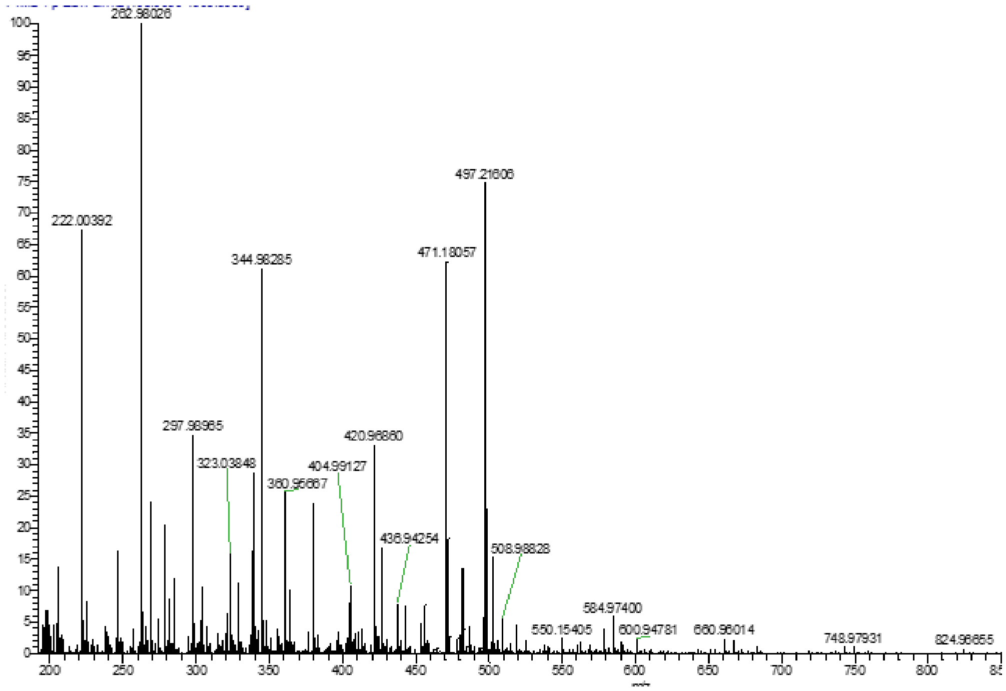

HRMS spectrum of D4

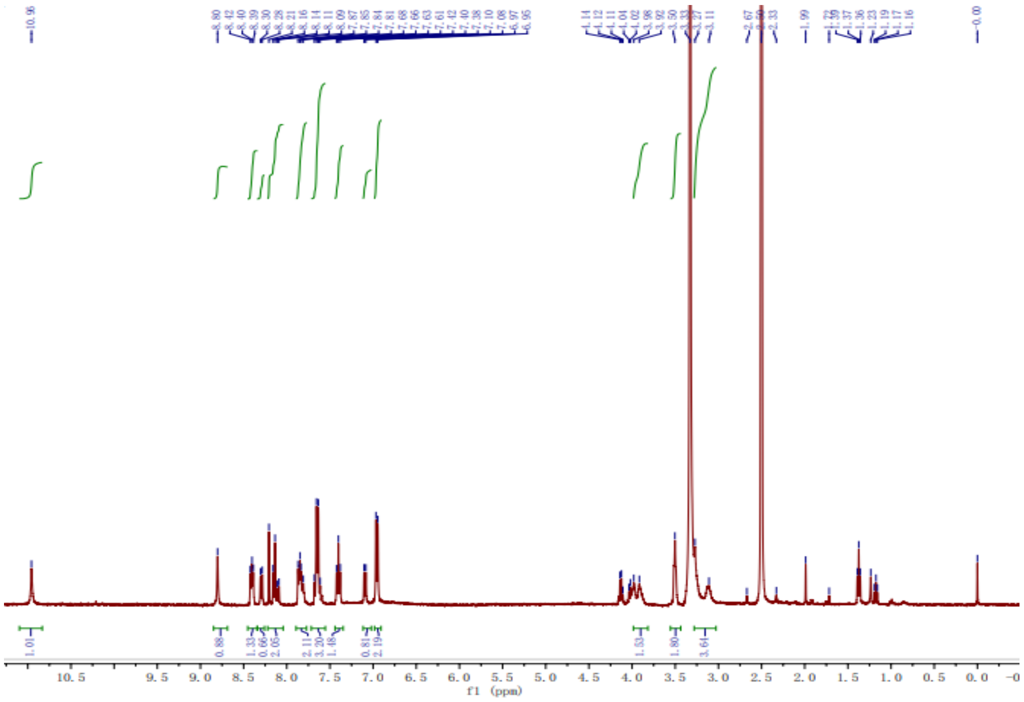

^{1}H-NMR spectrum of D4

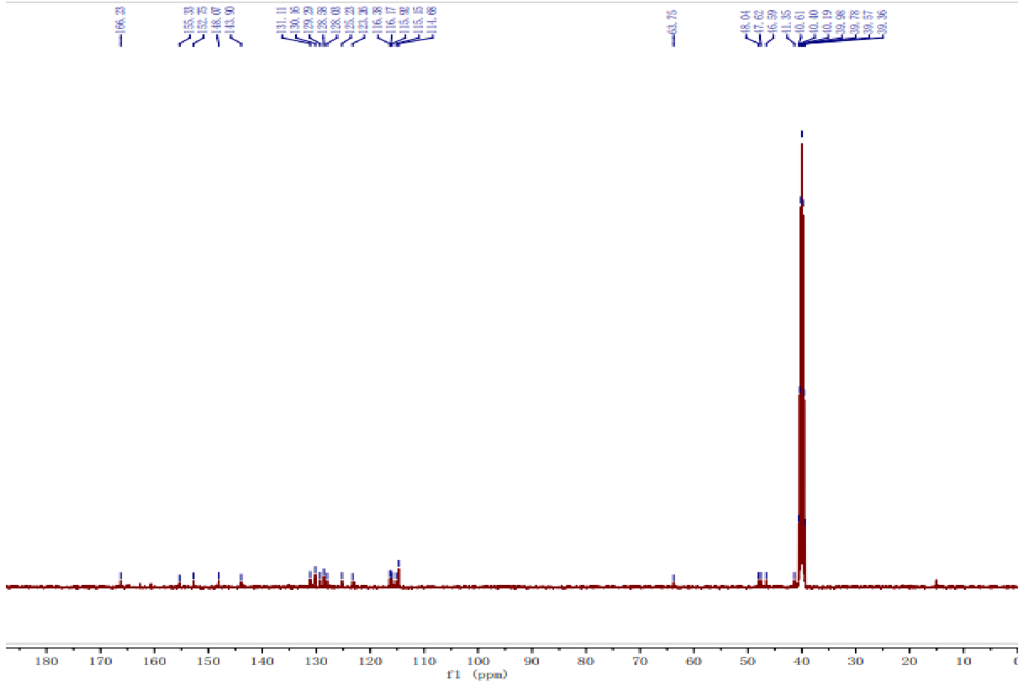

^{13}C-NMR spectrum of D4

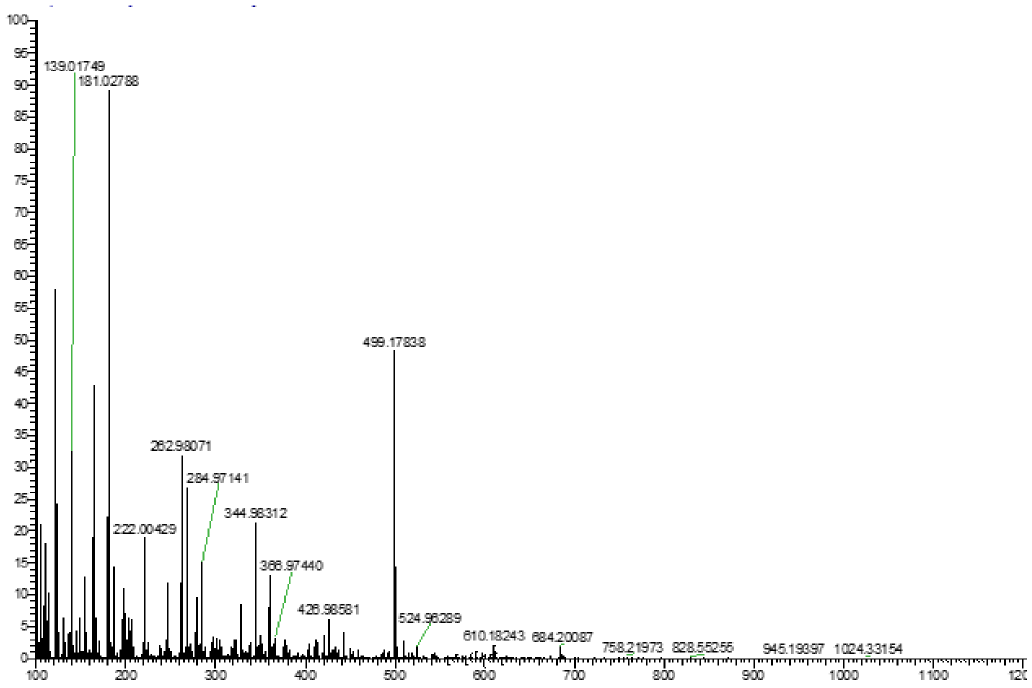

HRMS spectrum of D5

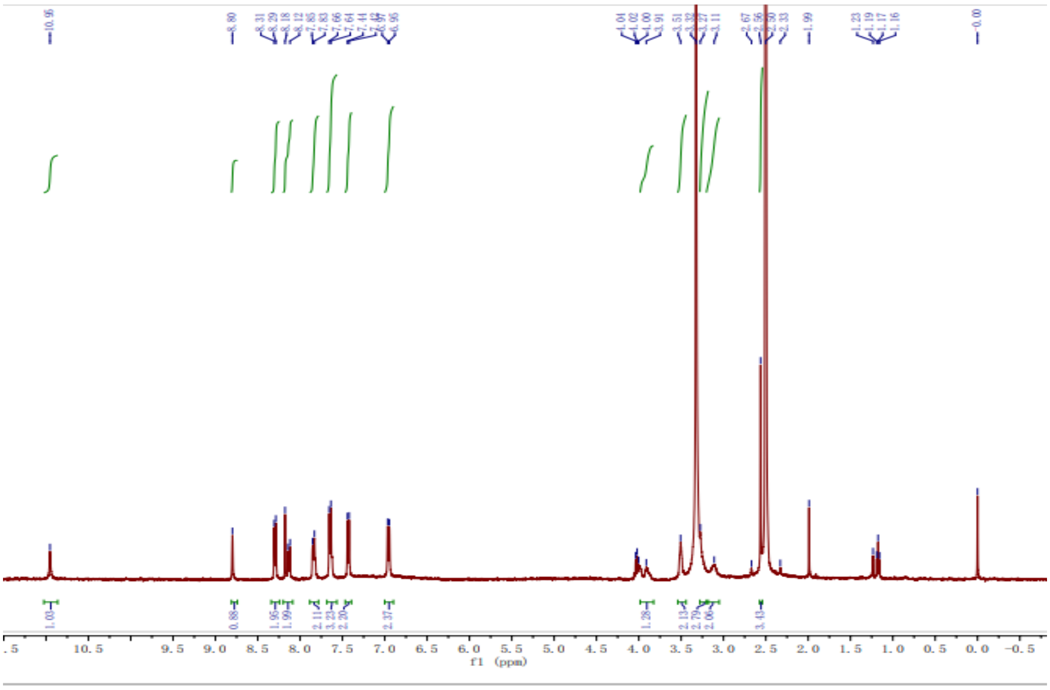

^{1}H-NMR spectrum of D5

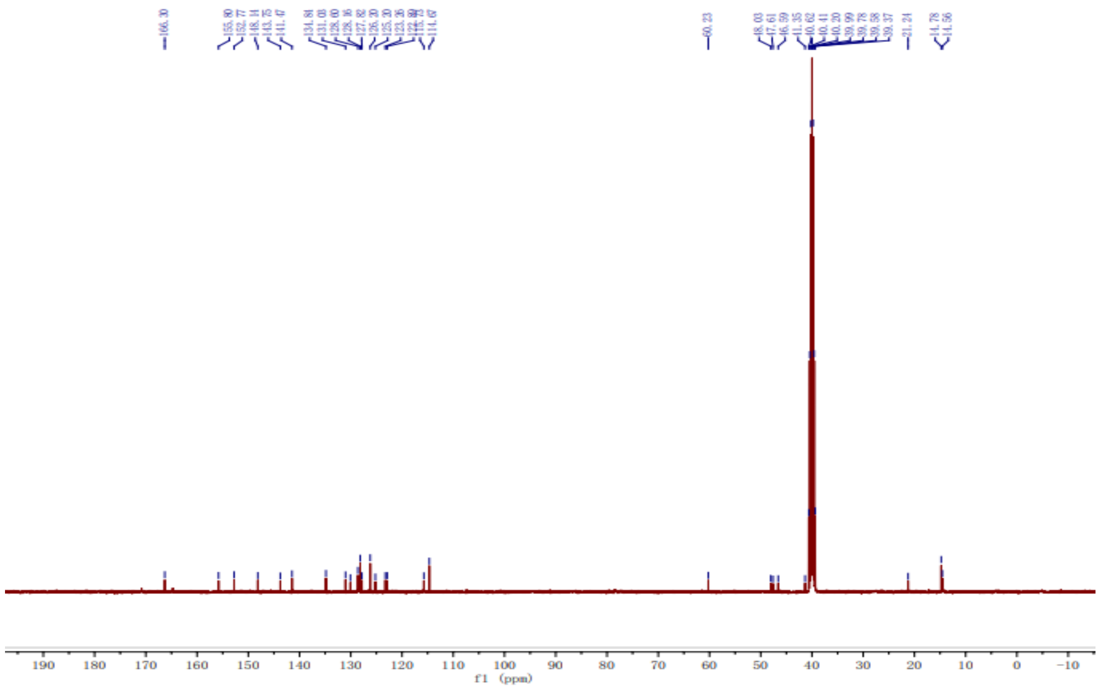

^{13}C-NMR spectrum of D5

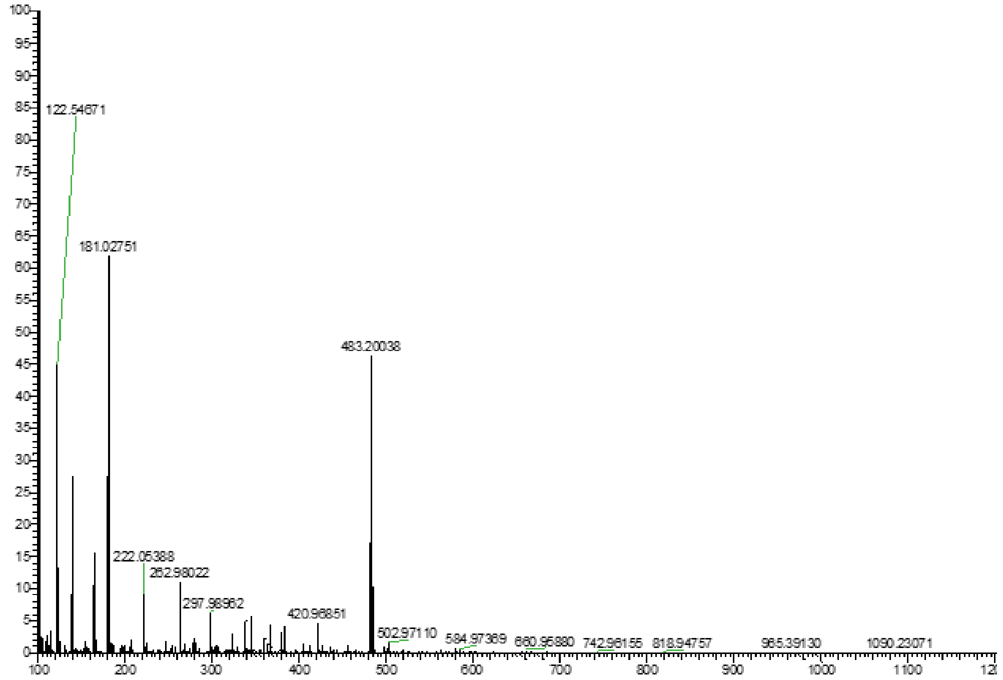

HRMS spectrum of D6

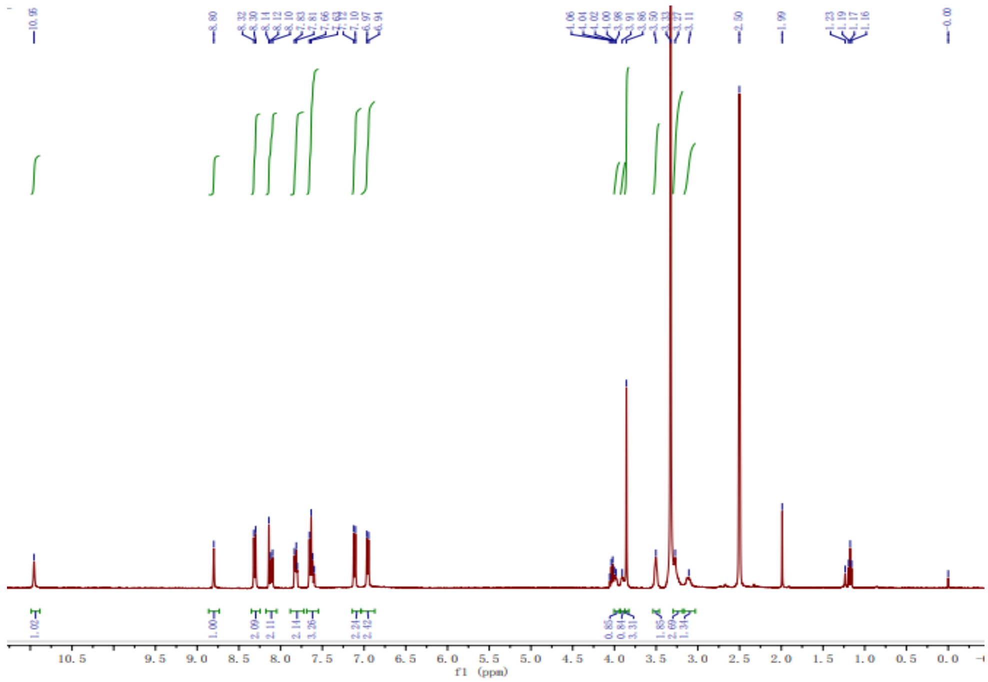

^{1}H-NMR spectrum of D6

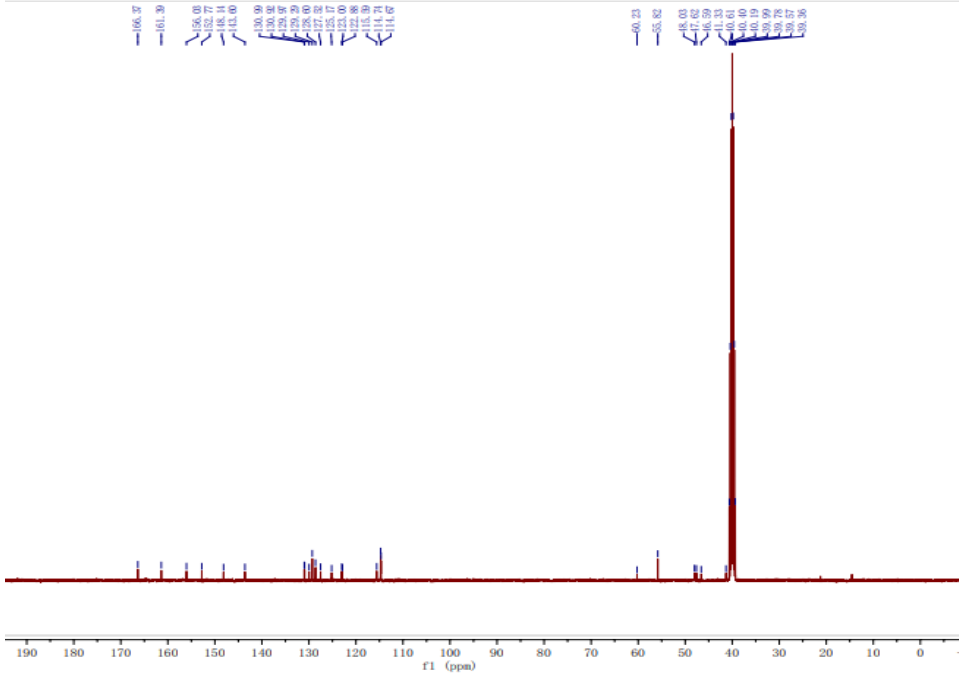

^{13}C-NMR spectrum of D6

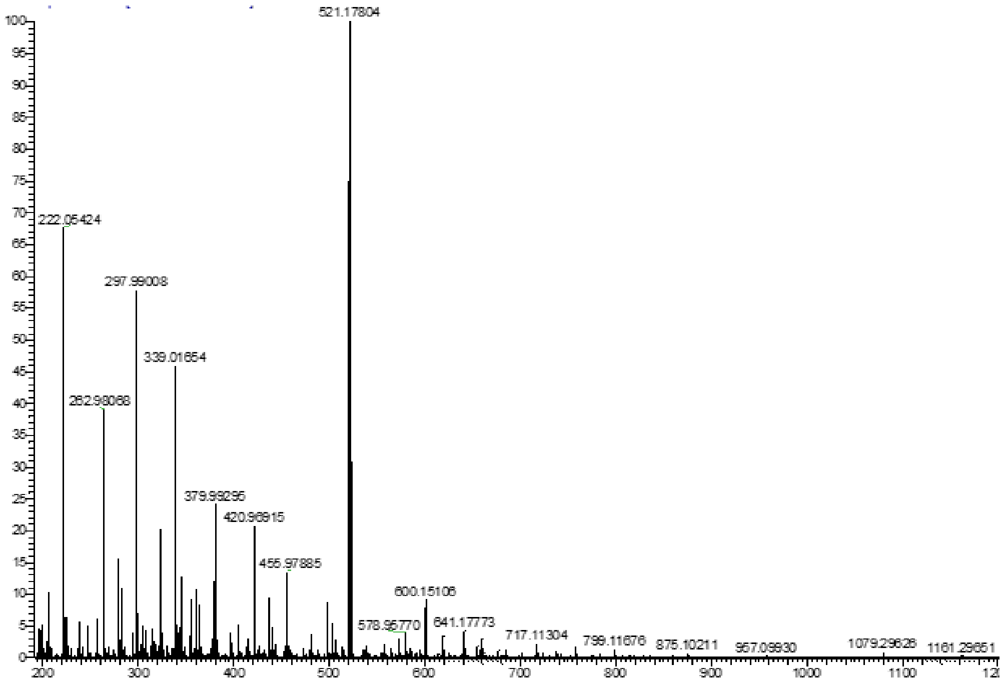

HRMS spectrum of D7

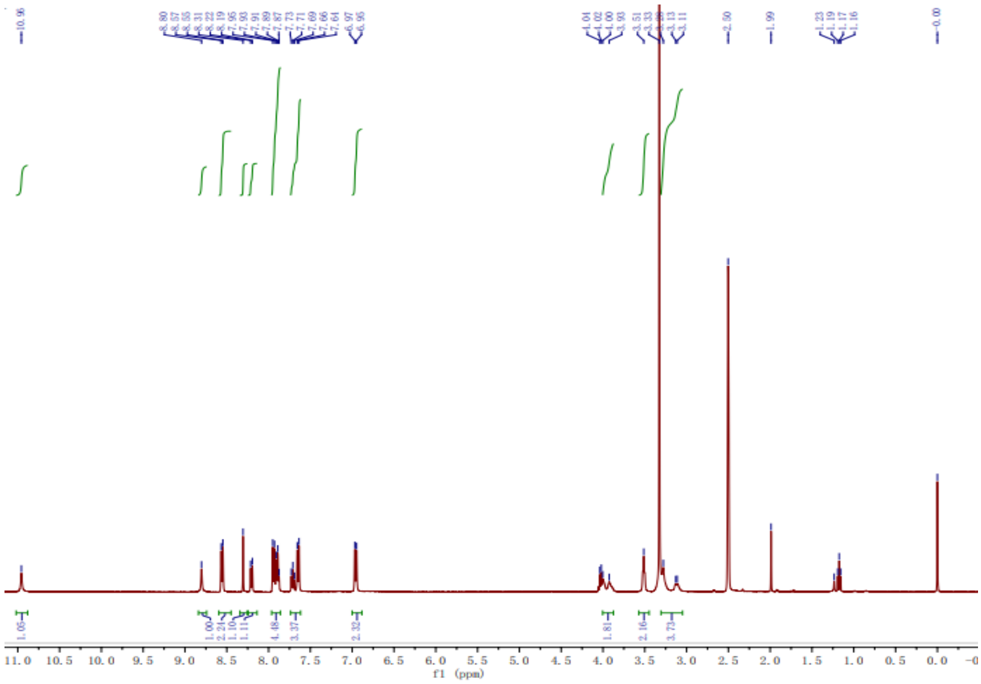

^{1}H-NMR spectrum of D7

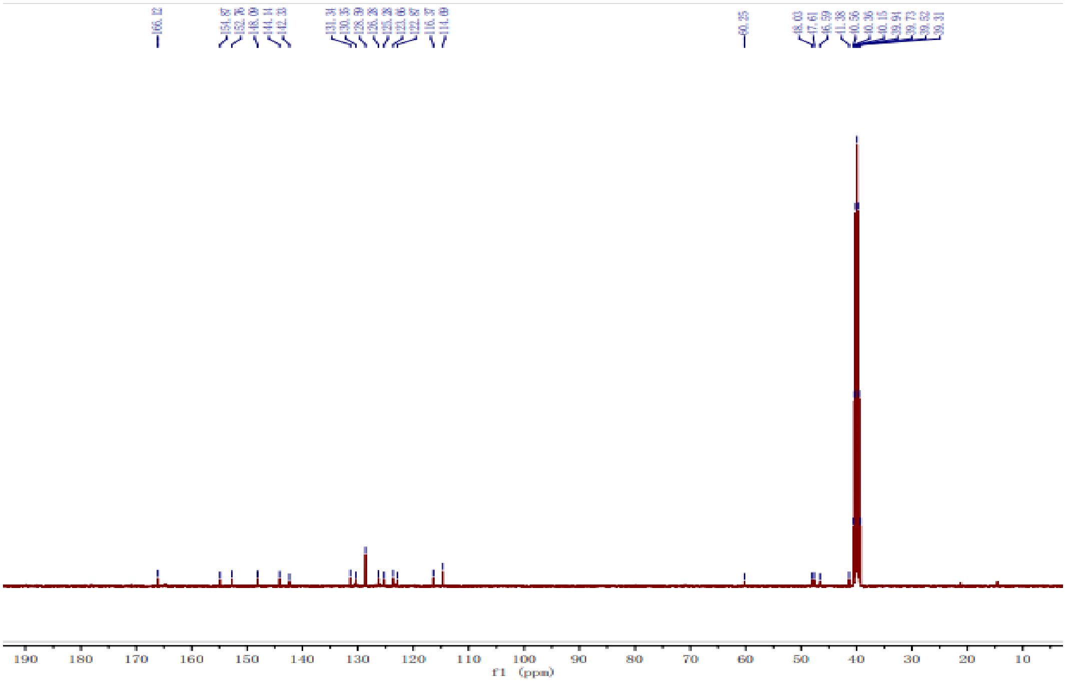

^{13}C-NMR spectrum of D7

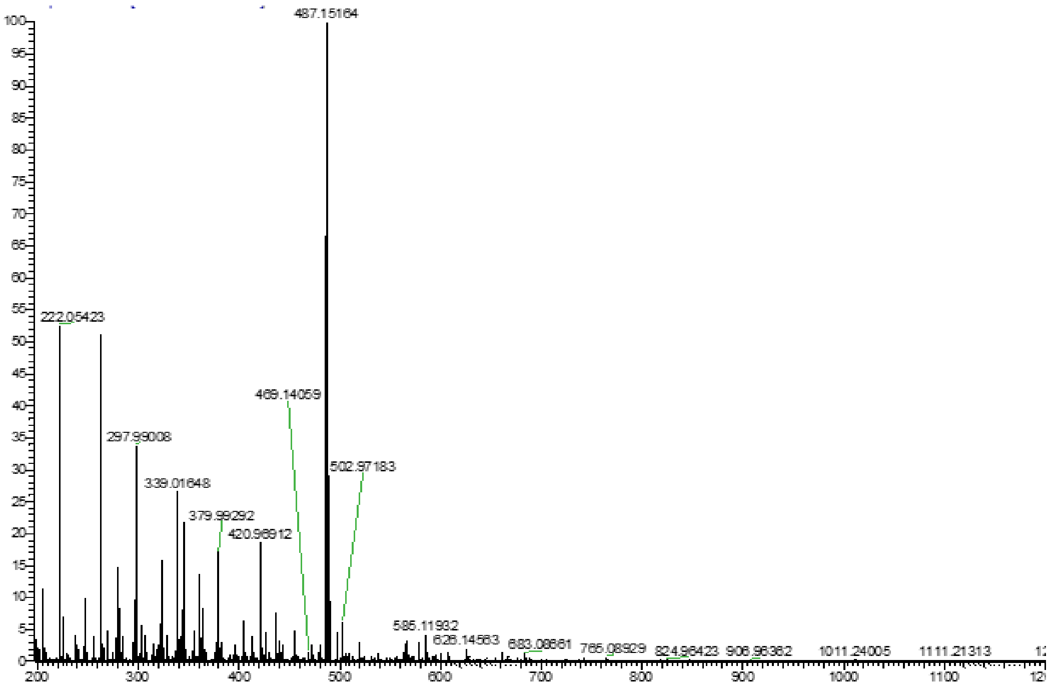

HRMS spectrum of D8

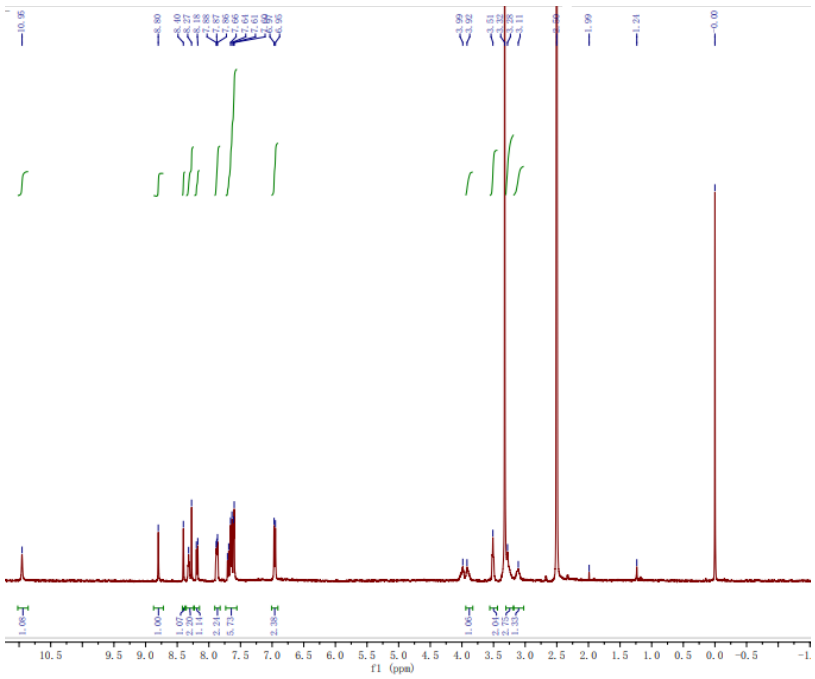

^{1}H-NMR spectrum of D8

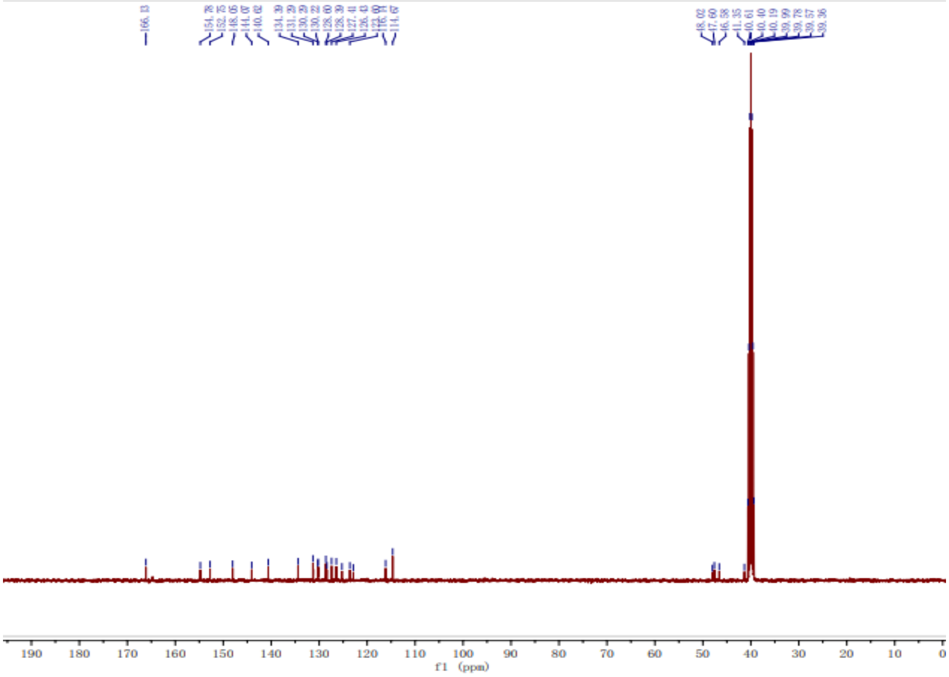

^{13}C-NMR spectrum of D8

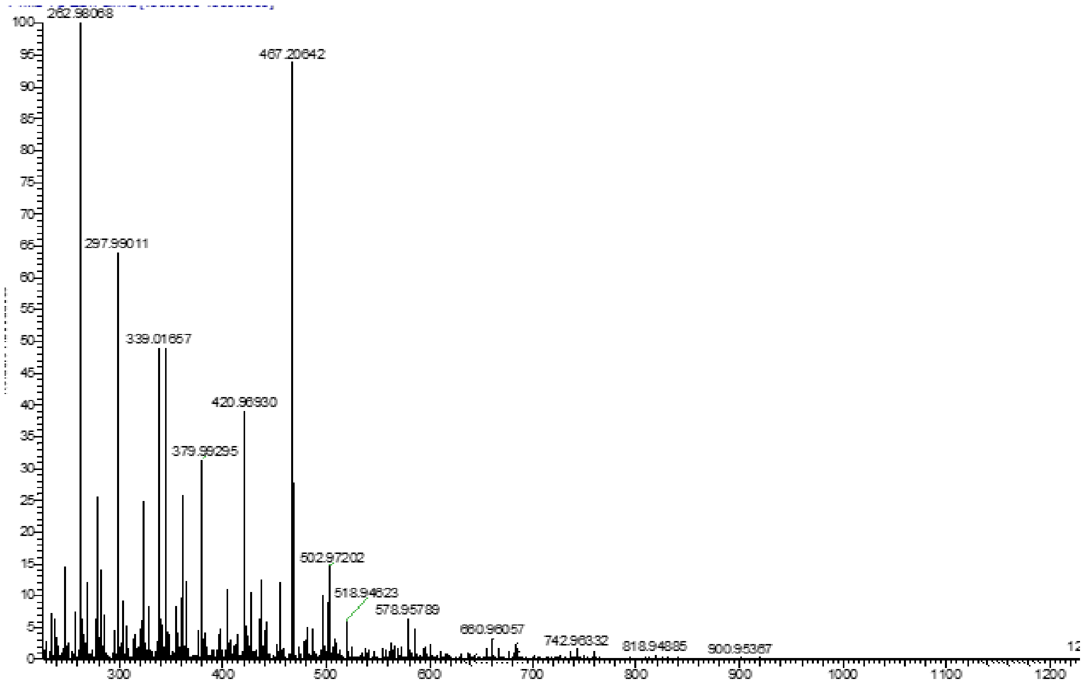

HRMS spectrum of D9

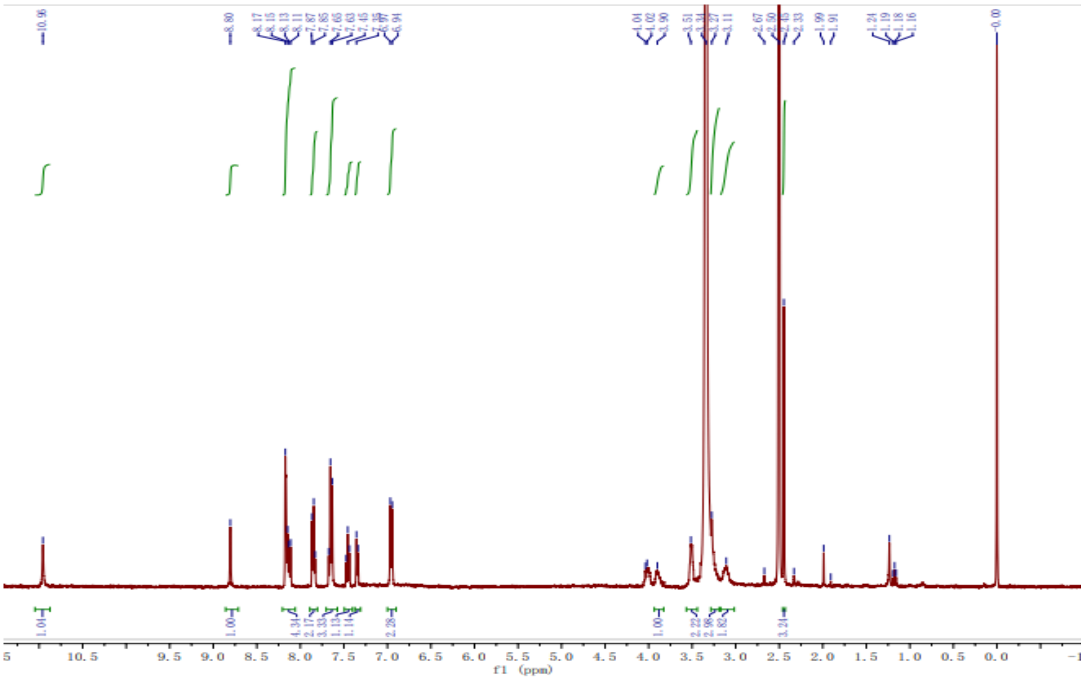

^{1}H-NMR spectrum of D9

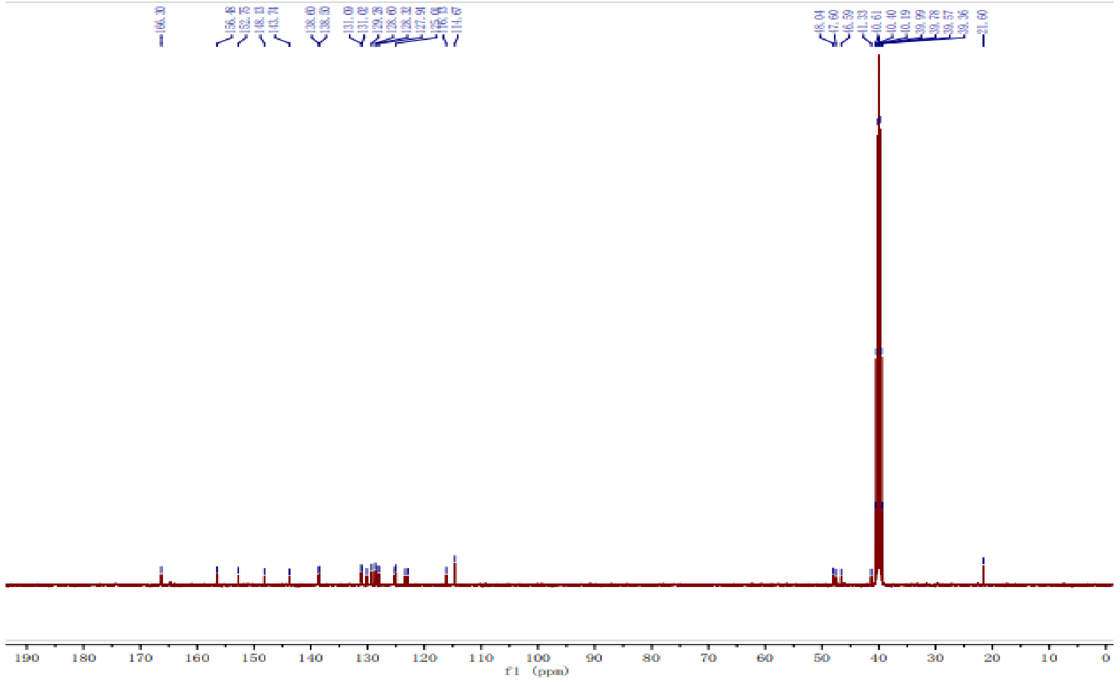

^{13}C-NMR spectrum of D9

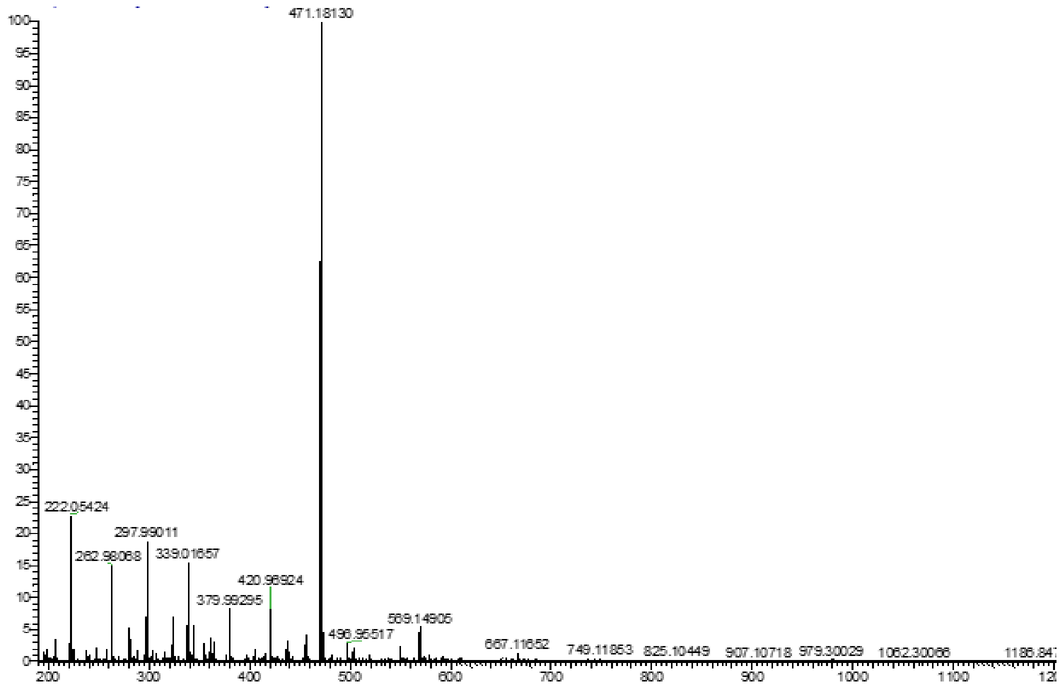

HRMS spectrum of D10

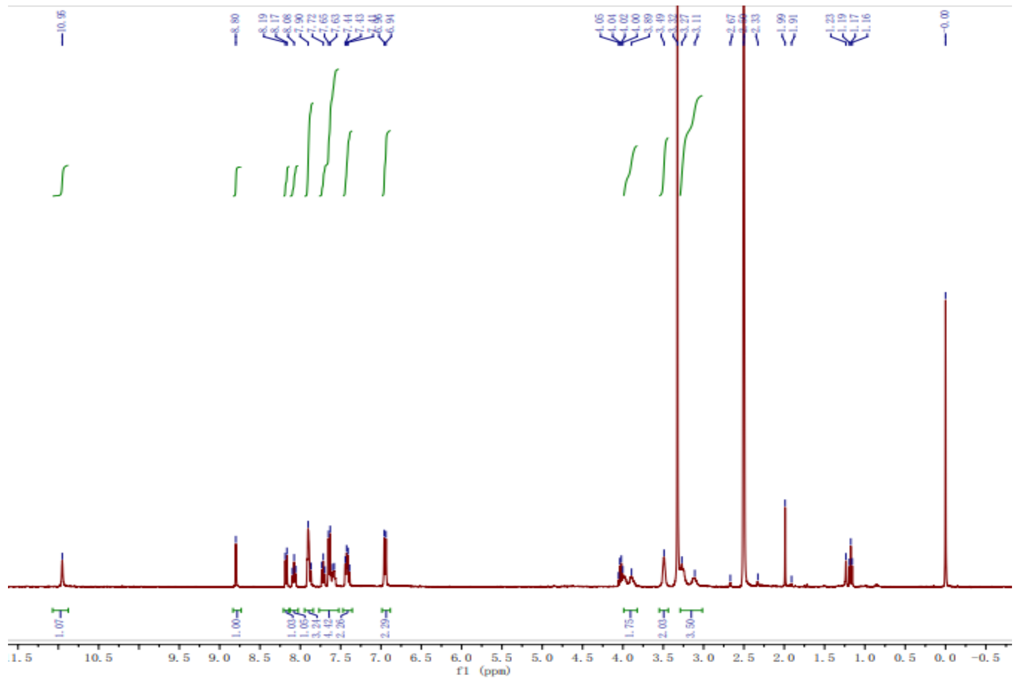

^{1}H-NMR spectrum of D10

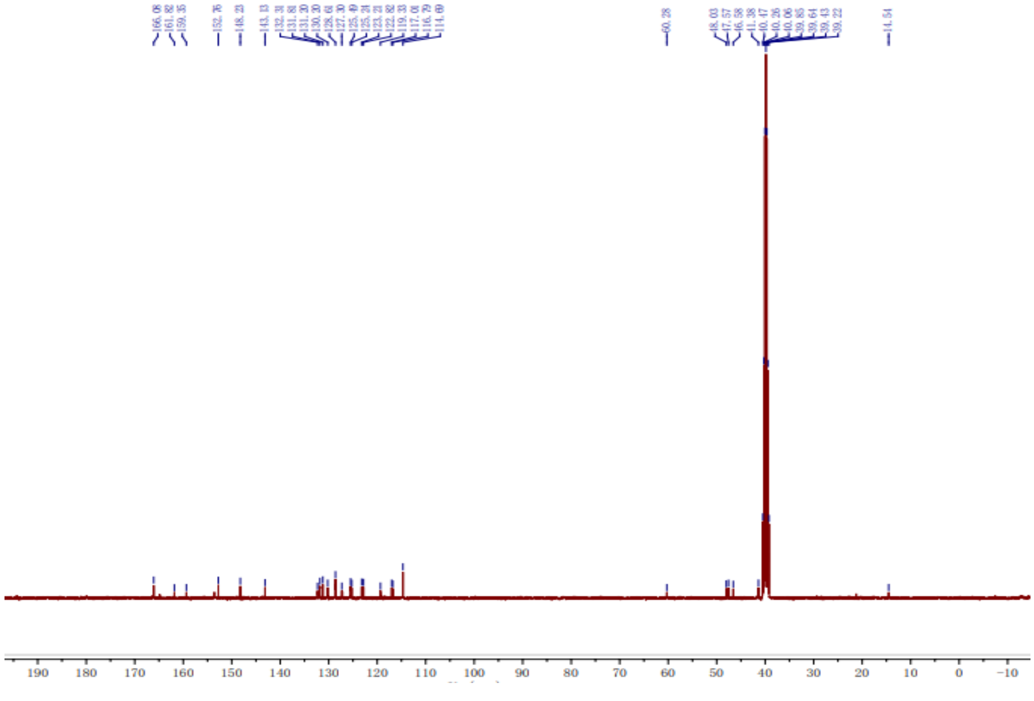

^{13}C-NMR spectrum of D10

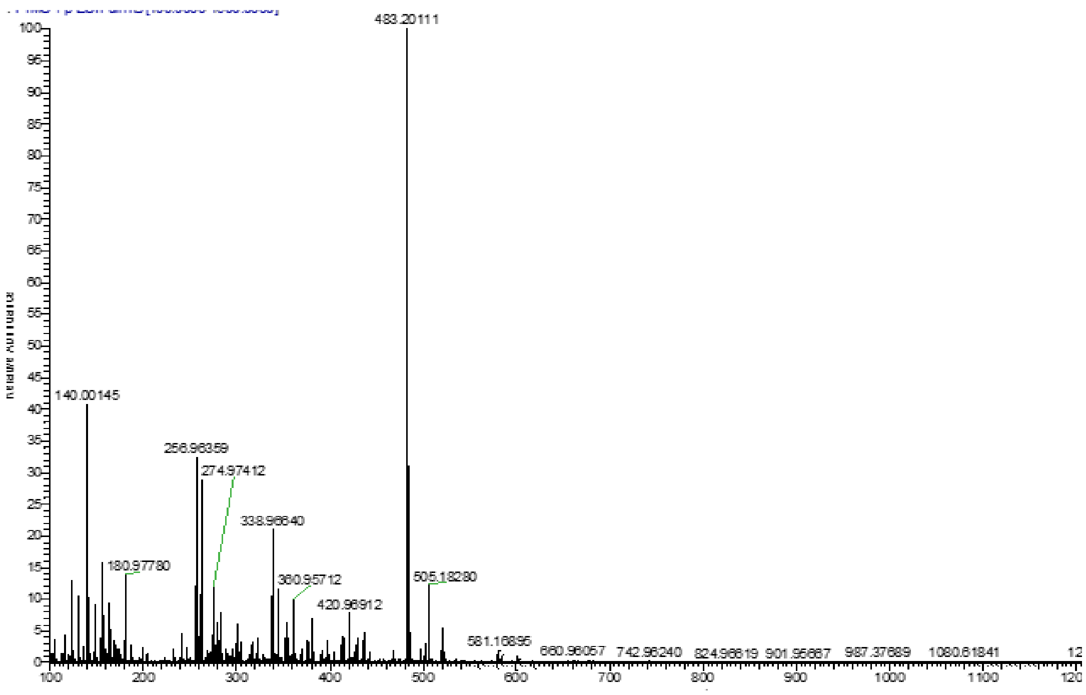

HRMS spectrum of D11

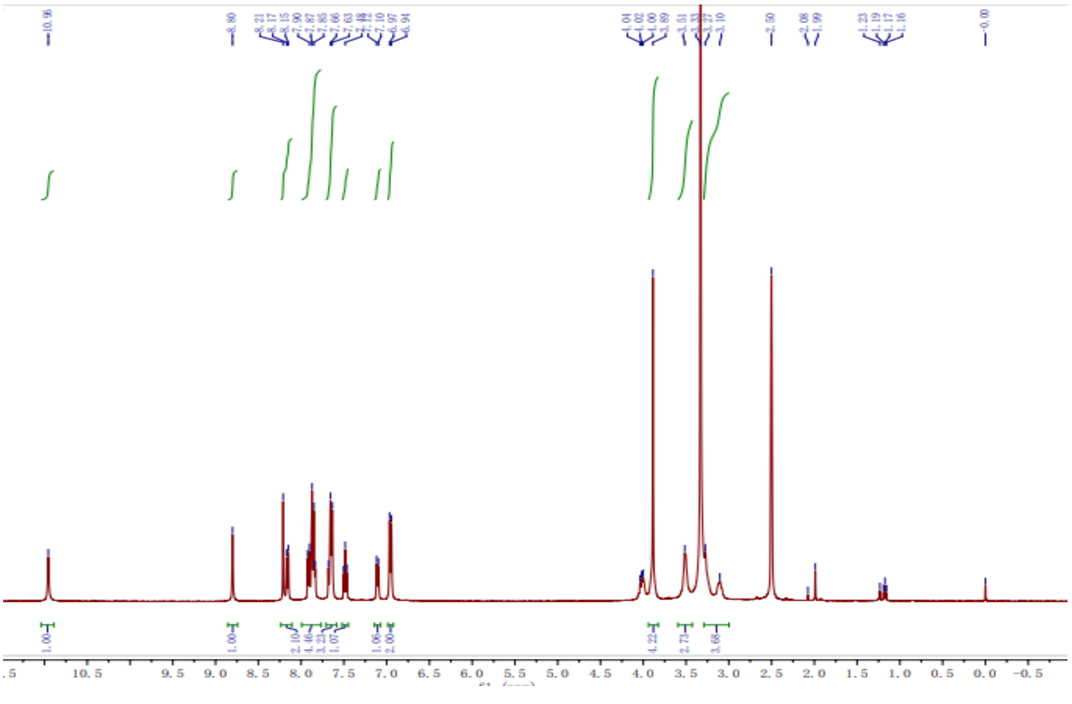

^{1}H-NMR spectrum of D11

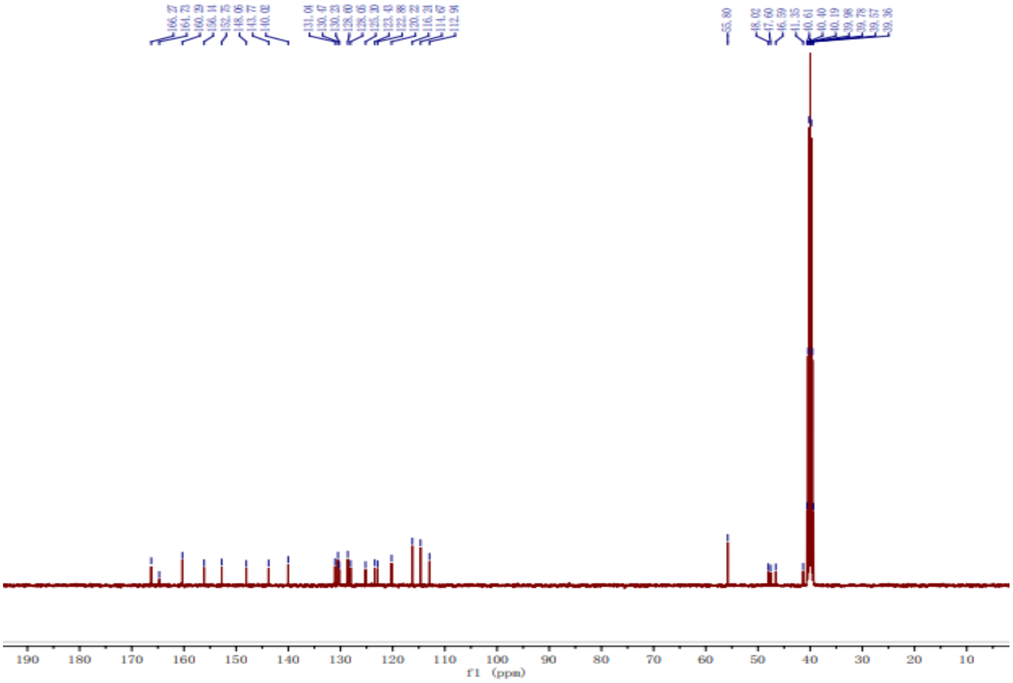

^{13}C-NMR spectrum of D11

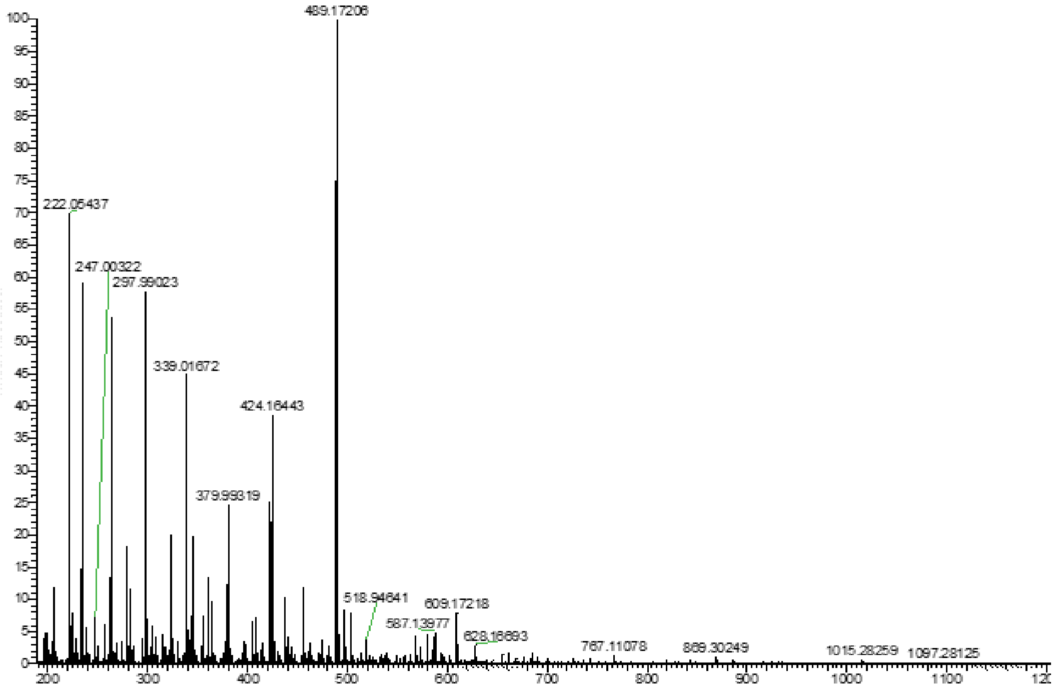

HRMS spectrum of D12

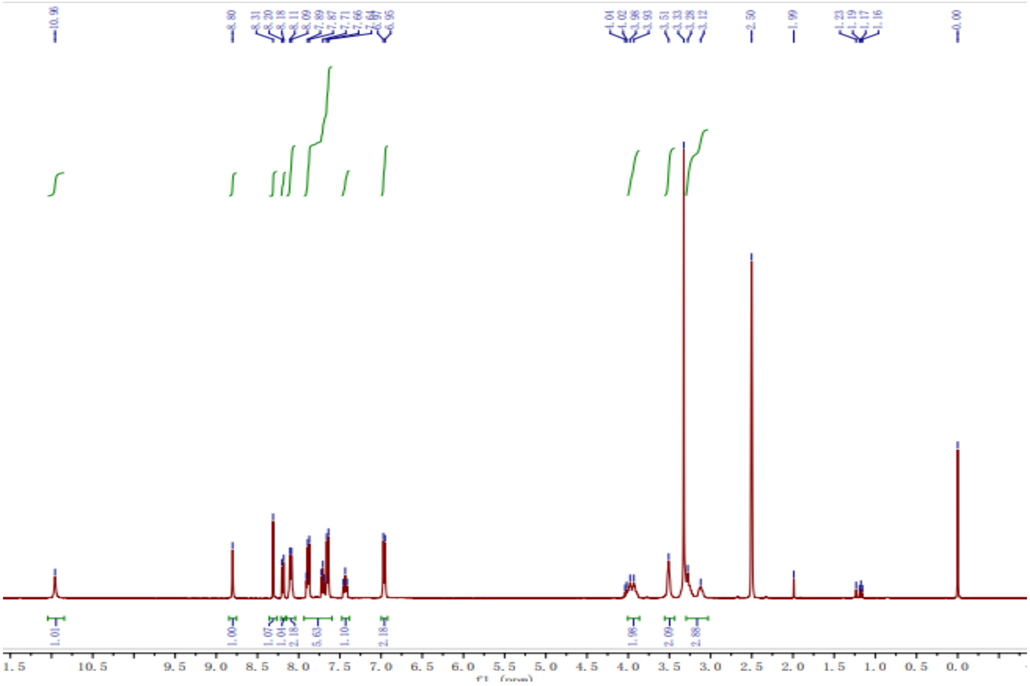

^{1}H-NMR spectrum of D12

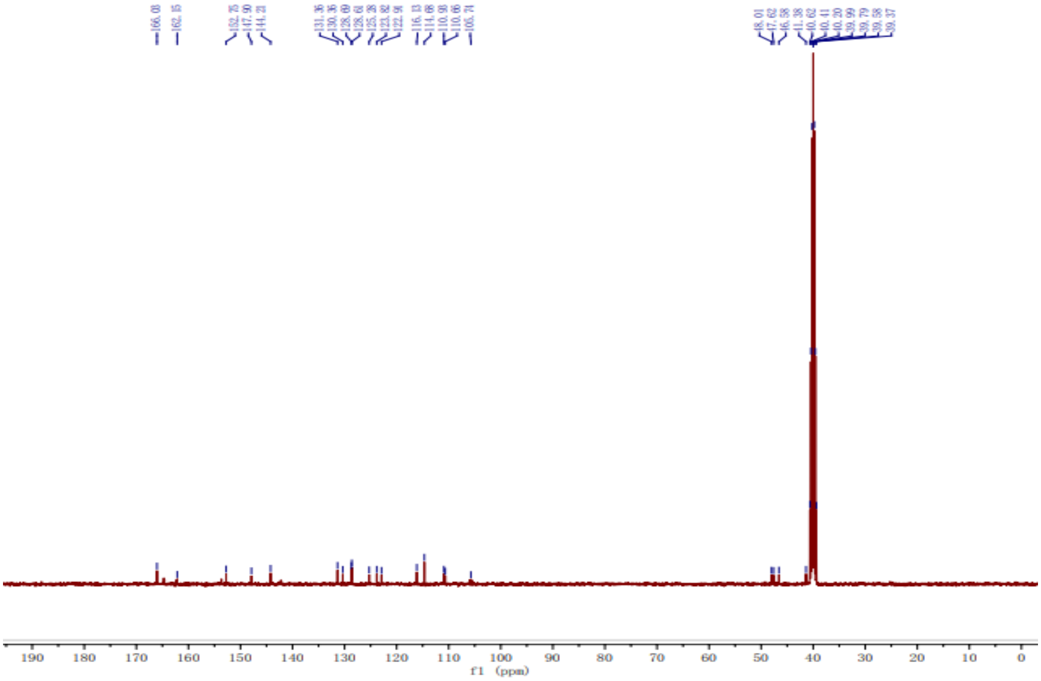

^{13}C-NMR spectrum of D12

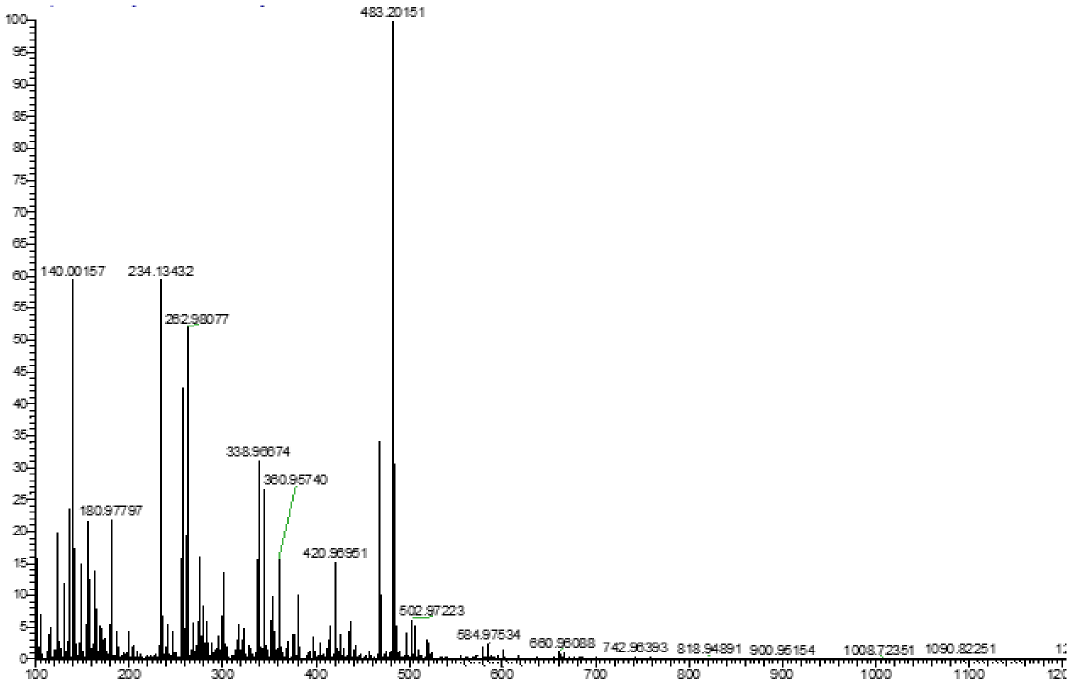

HRMS spectrum of D13

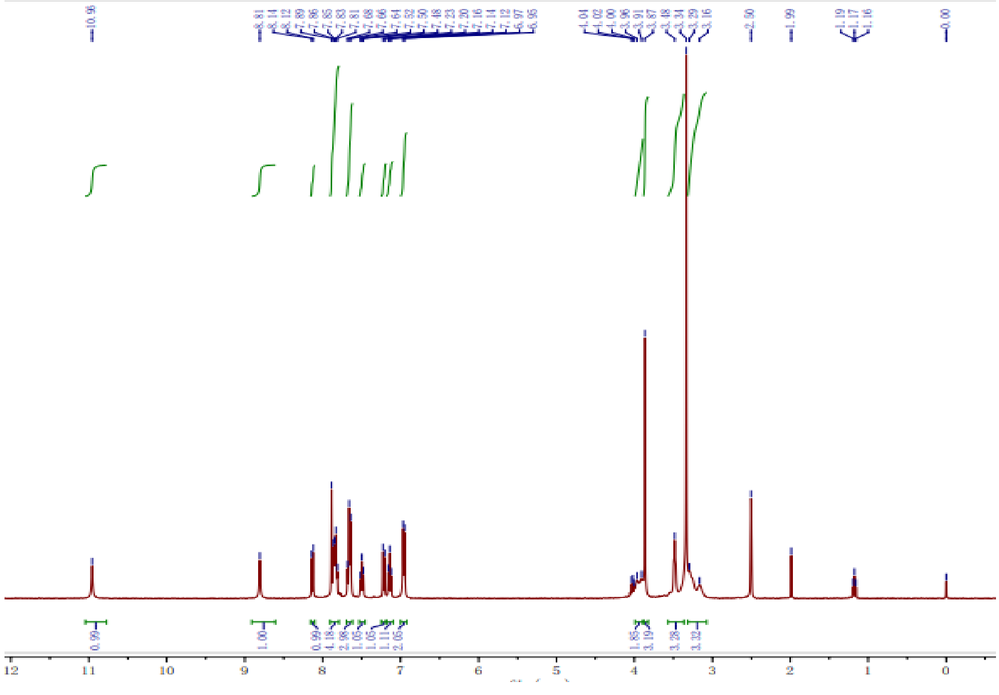

^{1}H-NMR spectrum of D13

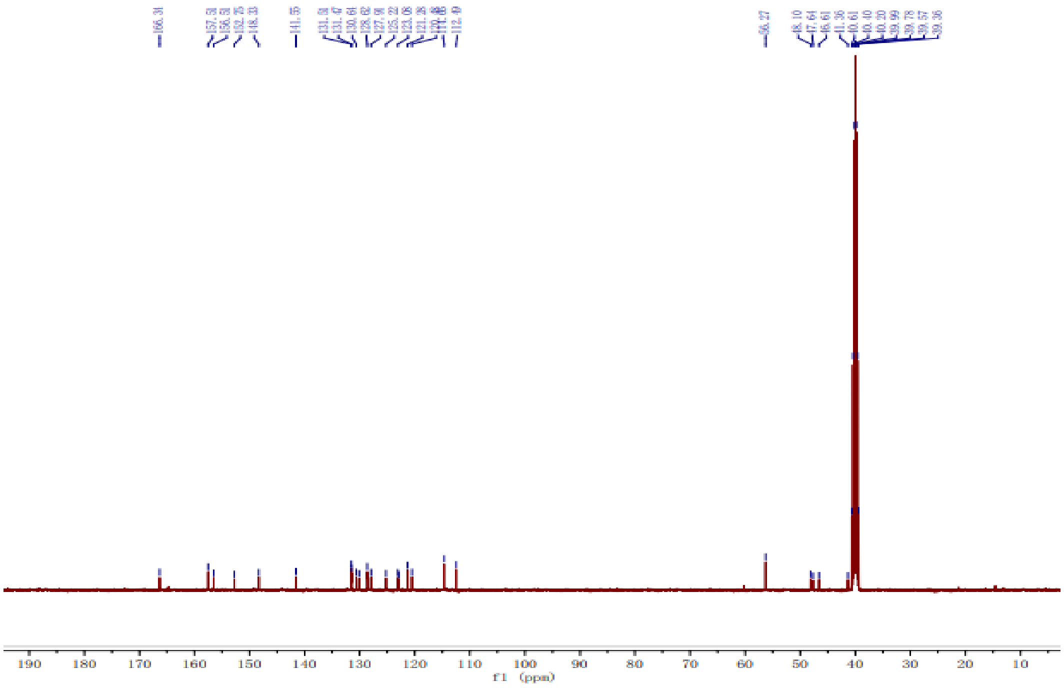

^{13}C-NMR spectrum of D13

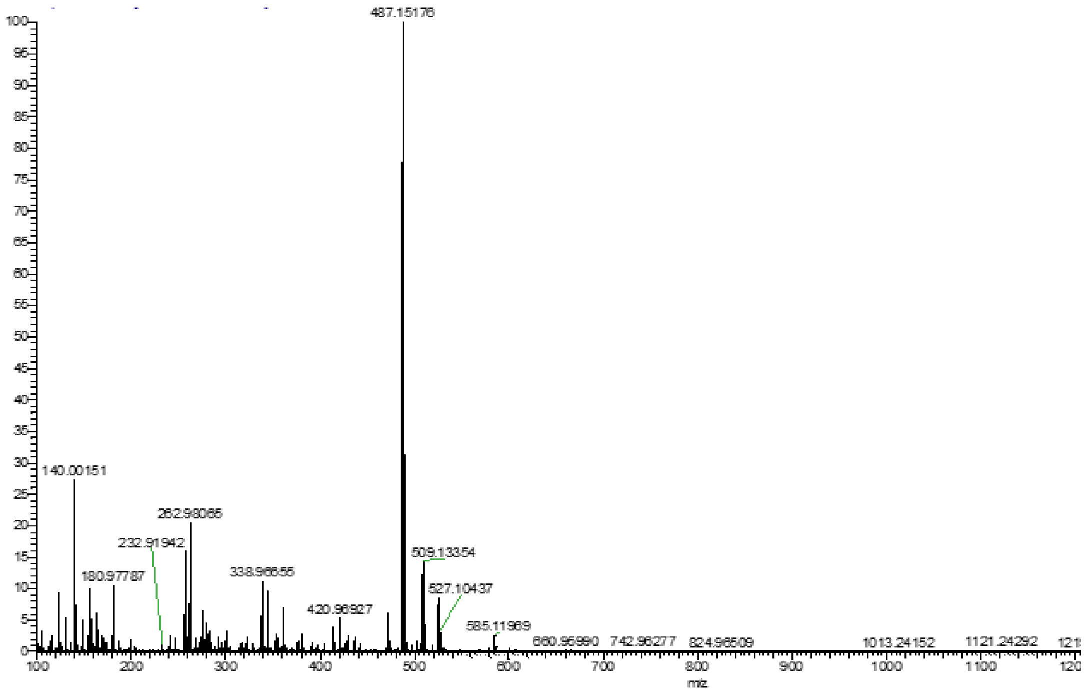

HRMS spectrum of D14

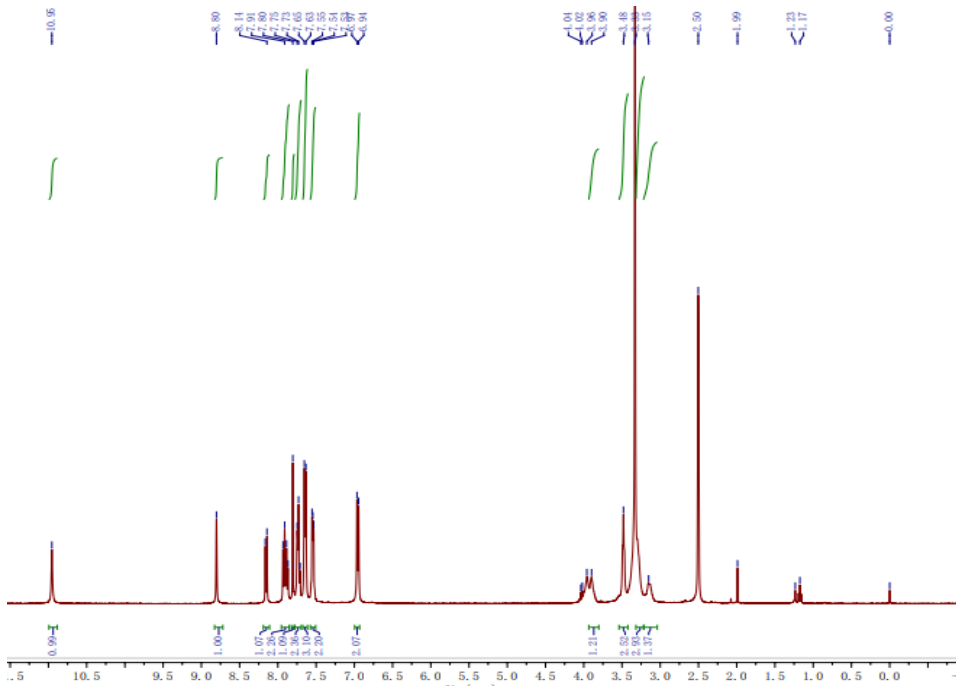

^{1}H-NMR spectrum of D14

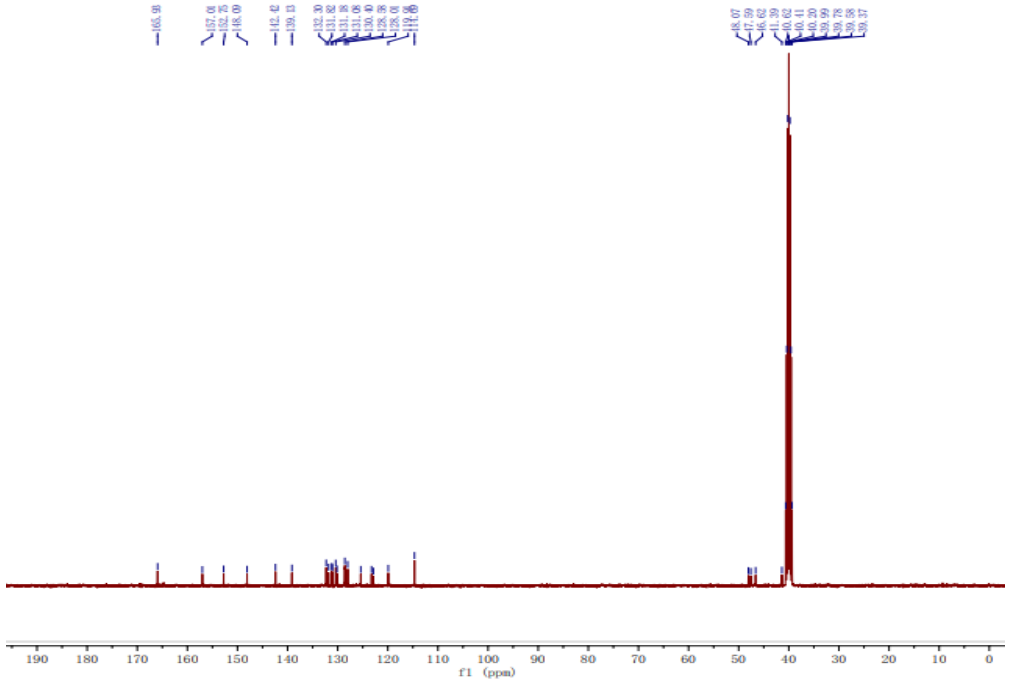

^{13}C-NMR spectrum of D14

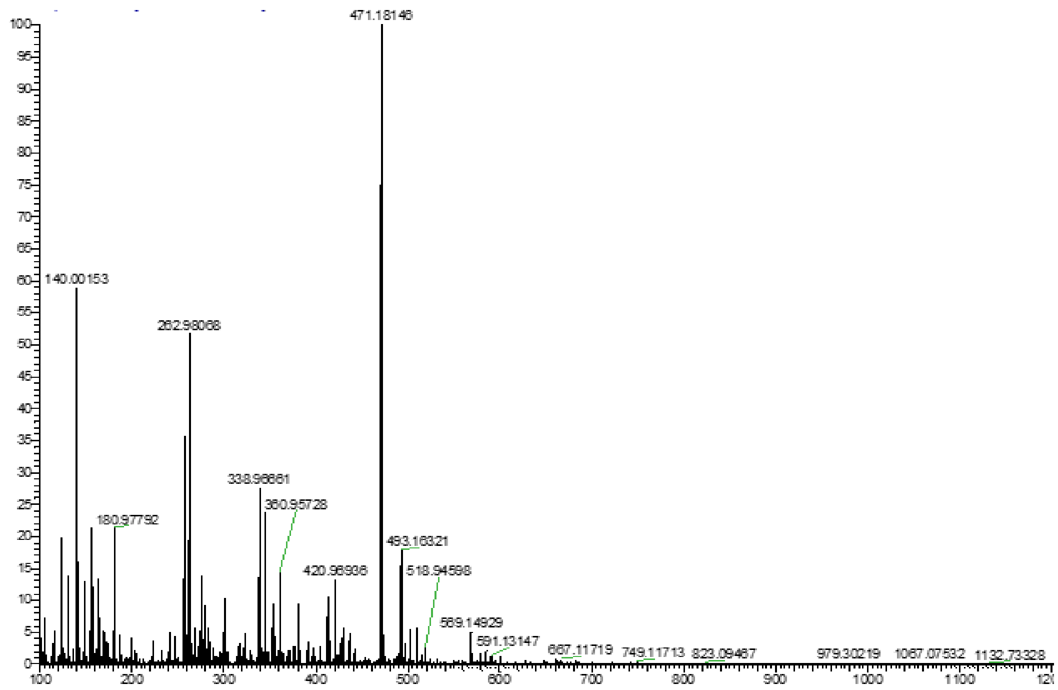

HRMS spectrum of D15

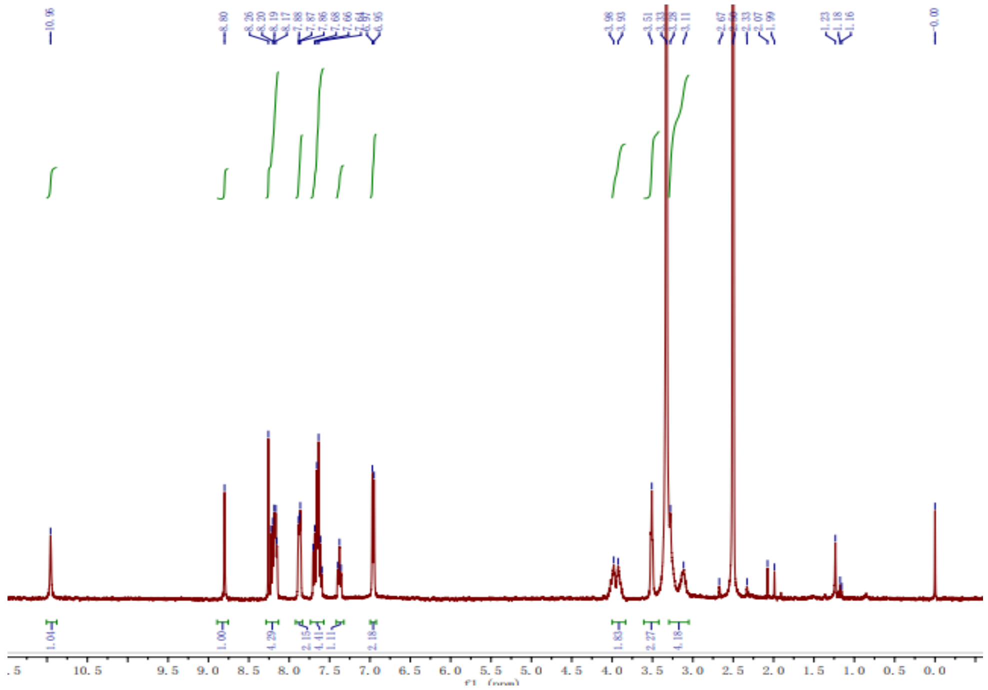

^{1}H-NMR spectrum of D15

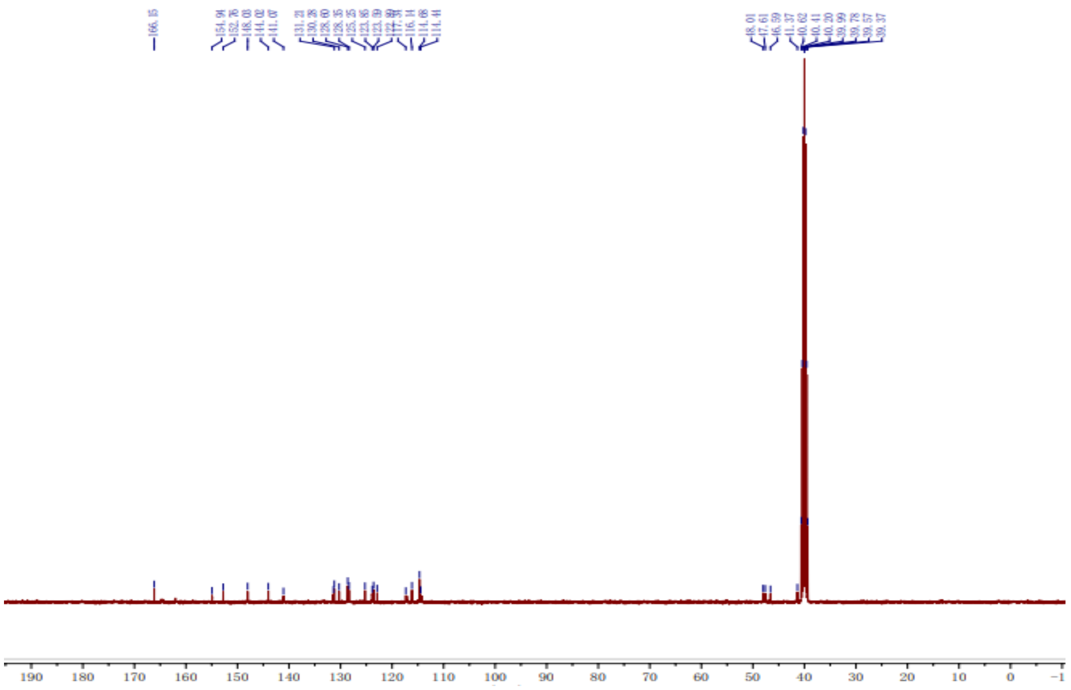

^{13}C-NMR spectrum of D15

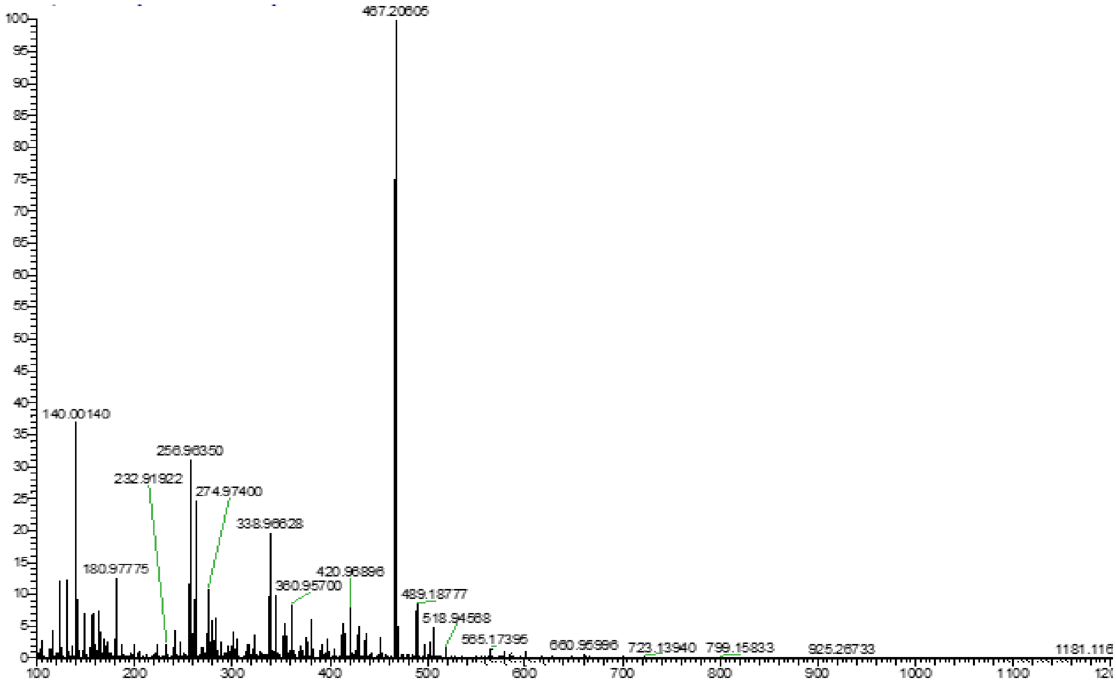

HRMS spectrum of D16

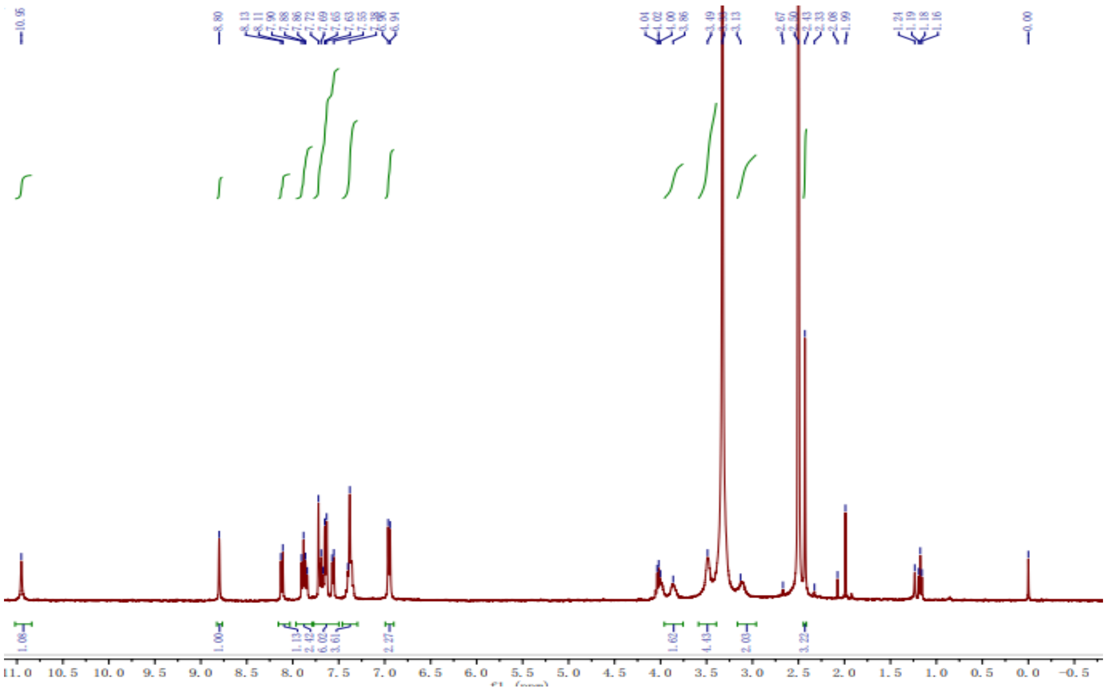

^{1}H-NMR spectrum of D16

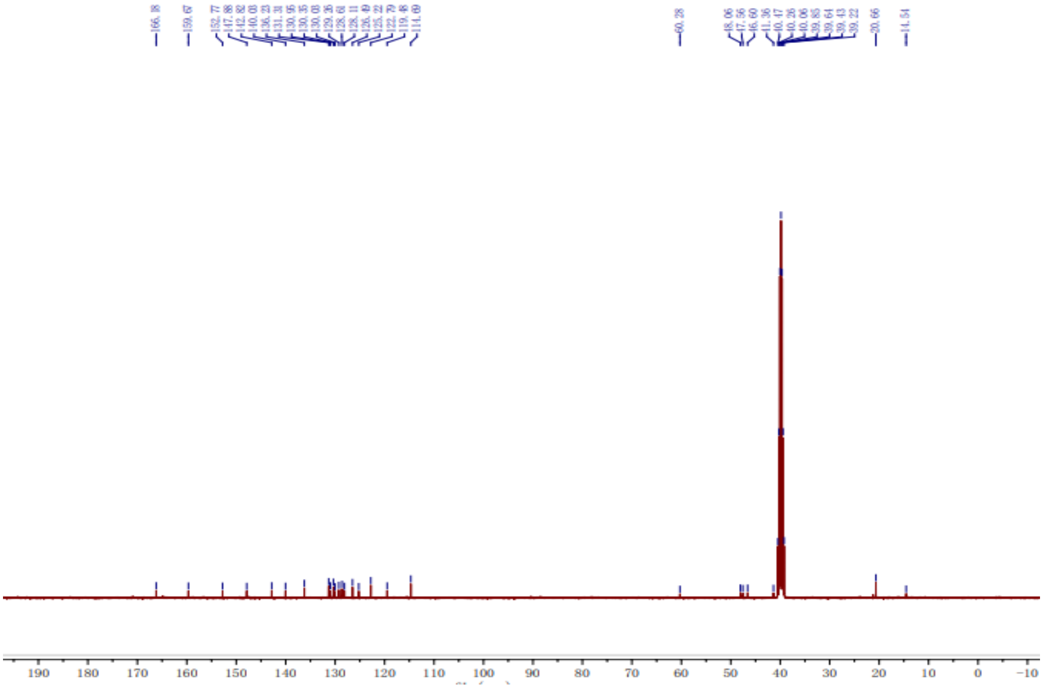

^{13}C-NMR spectrum of D16

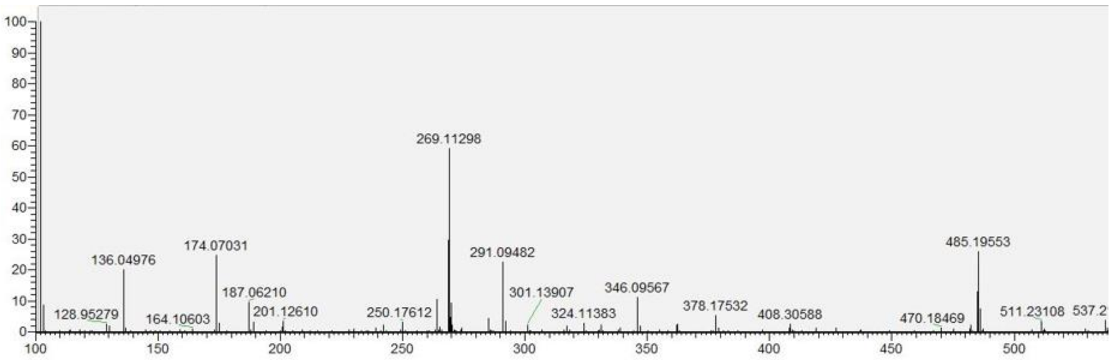

HRMS spectrum of D17

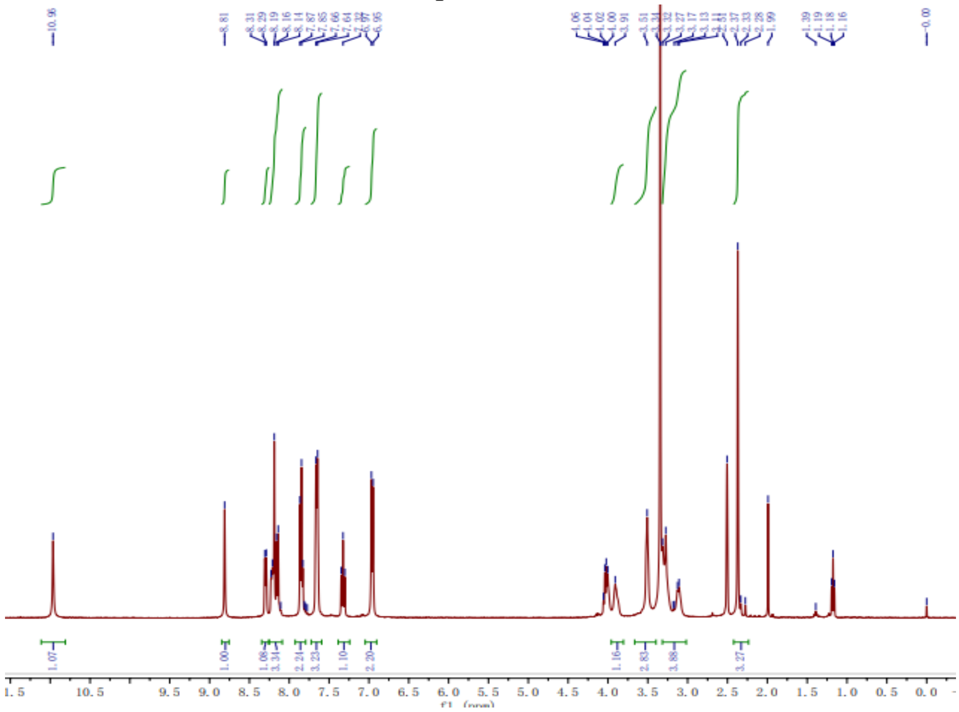

^{1}H-NMR spectrum of D17

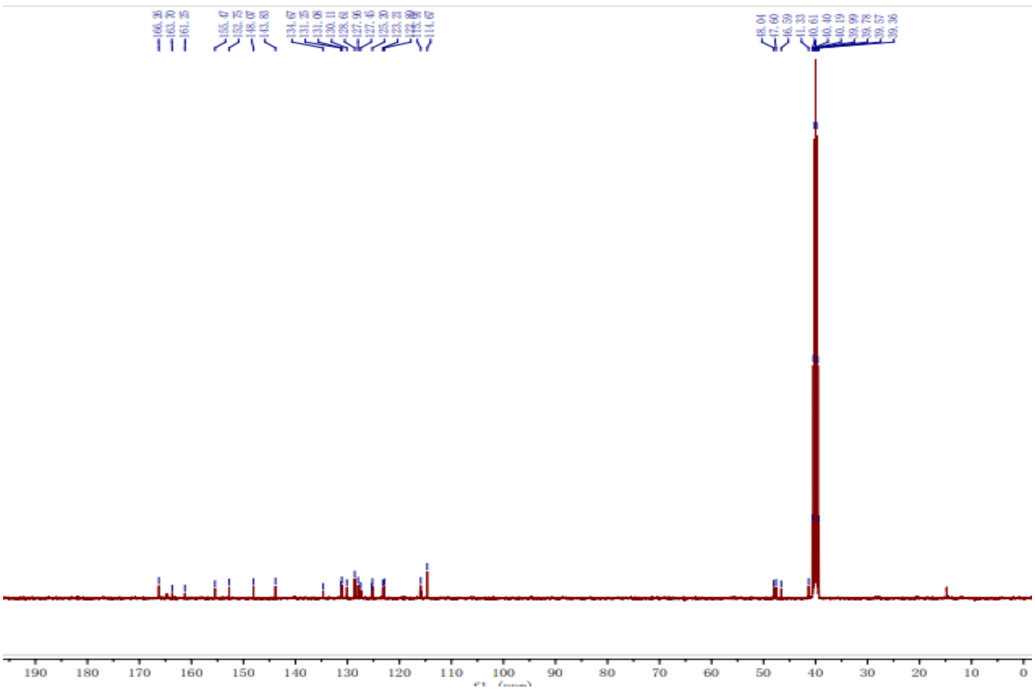

^{13}C-NMR spectrum of D17

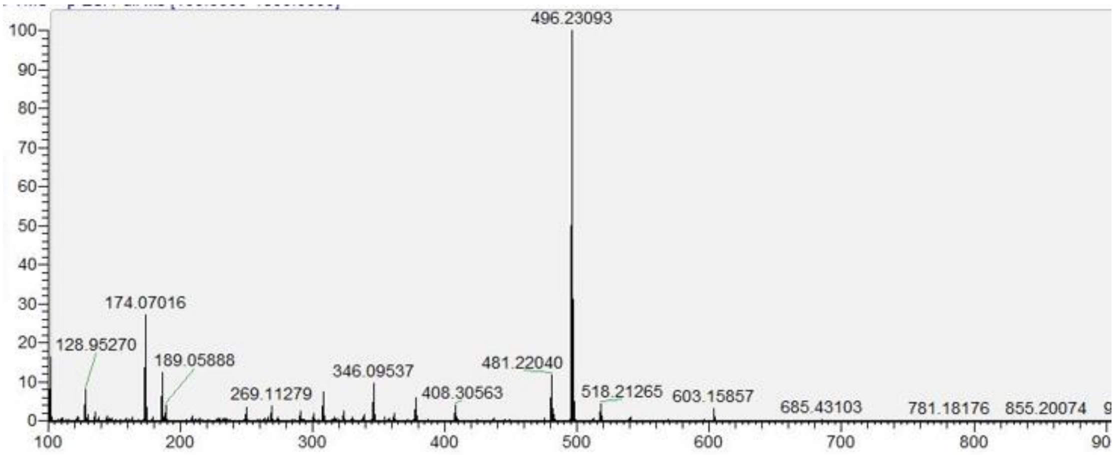

HRMS spectrum of D18

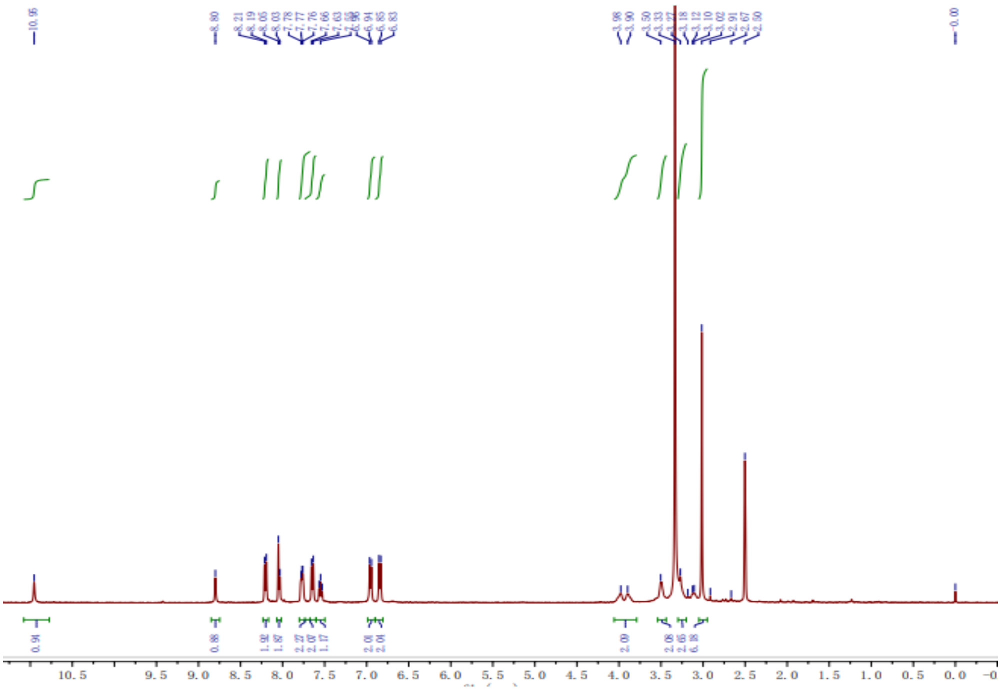

^{1}H-NMR spectrum of D18

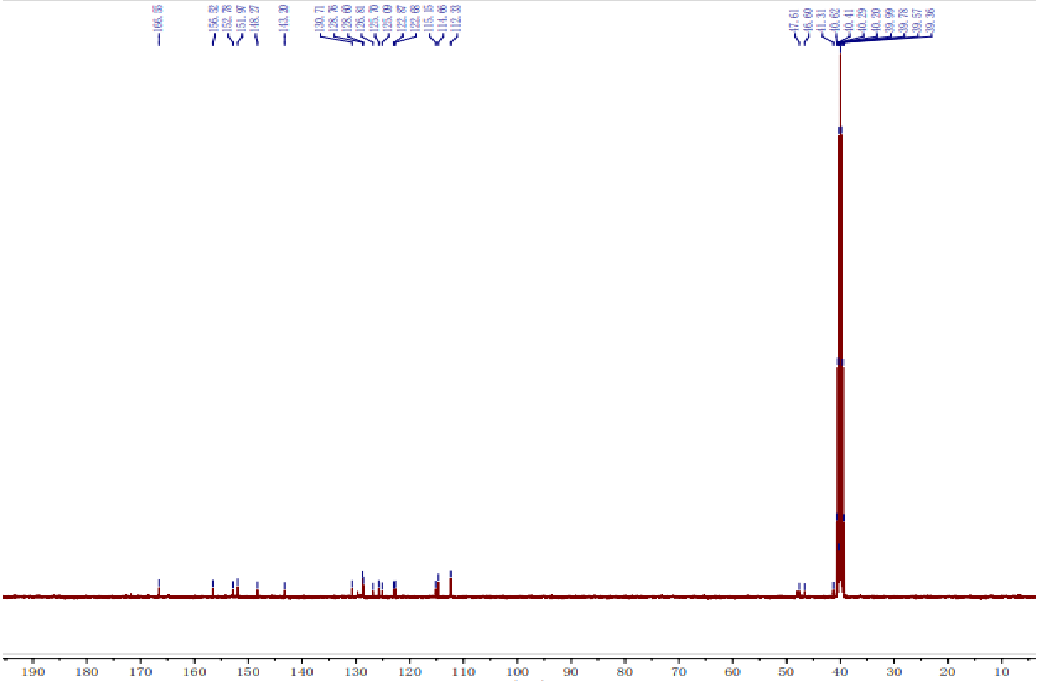

^{13}C-NMR spectrum of D18

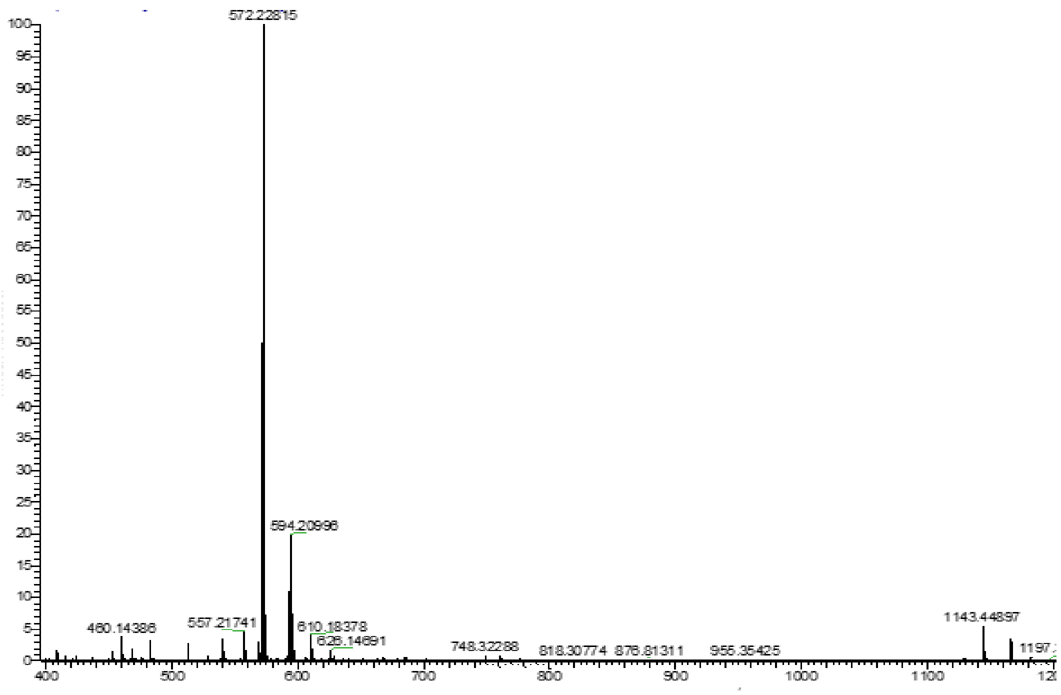

HRMS spectrum of D19

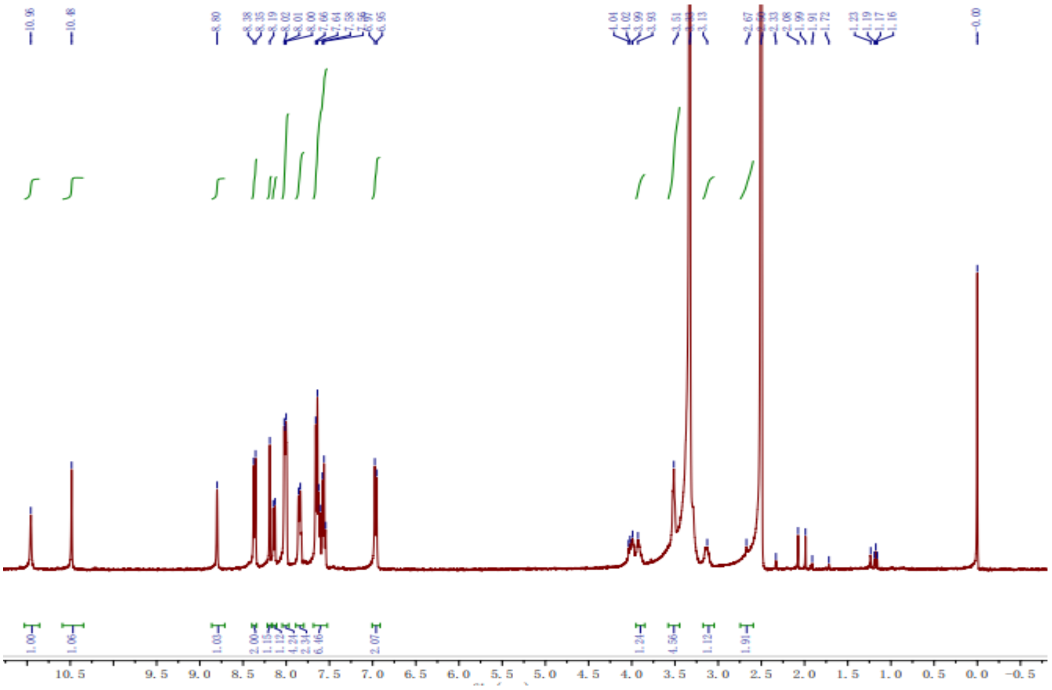

^{1}H-NMR spectrum of D19

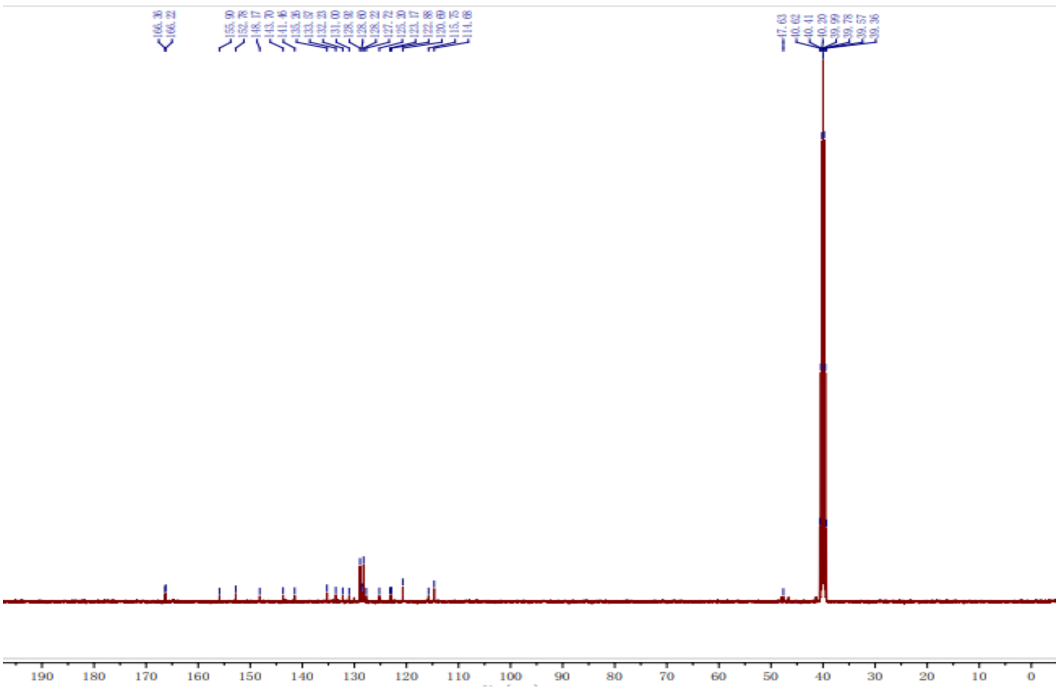

^{13}C-NMR spectrum of D19

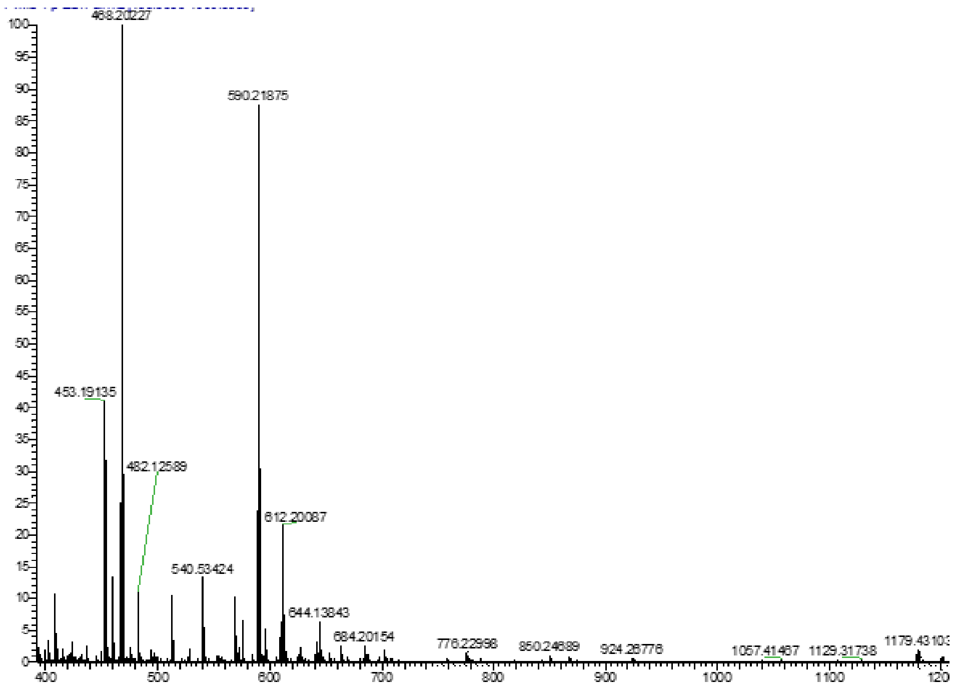

HRMS spectrum of D20

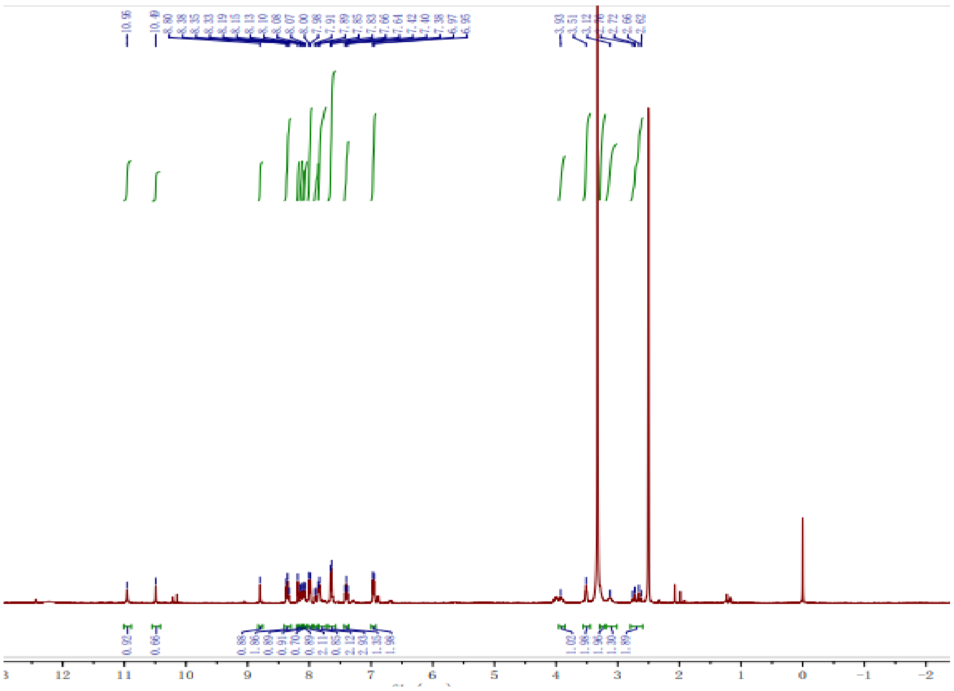

^{1}H-NMR spectrum of D20

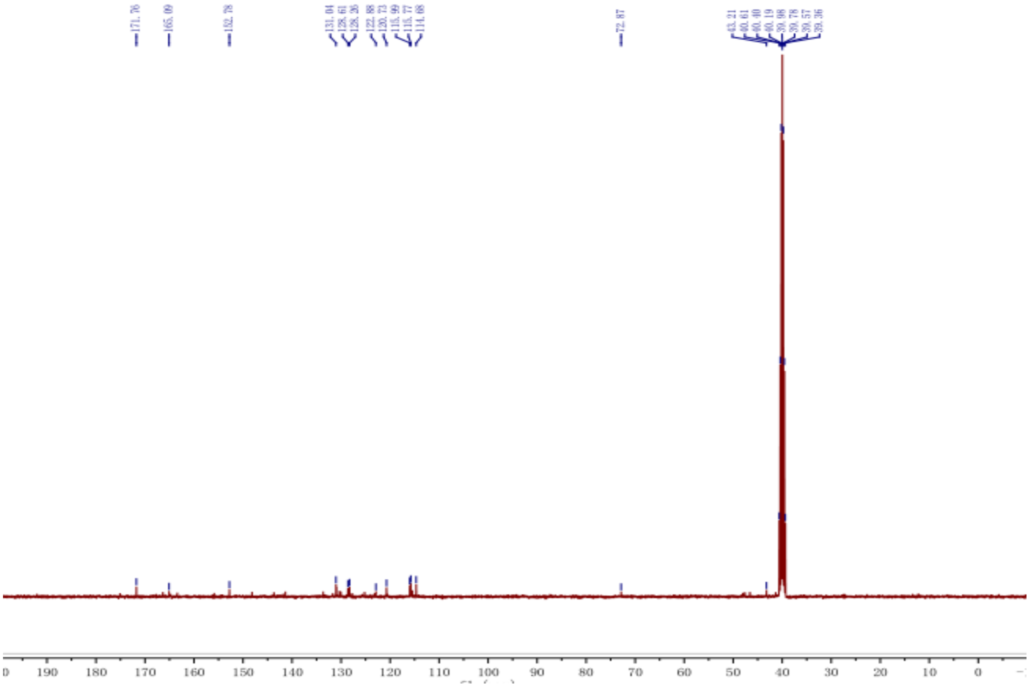

^{13}C-NMR spectrum of D20

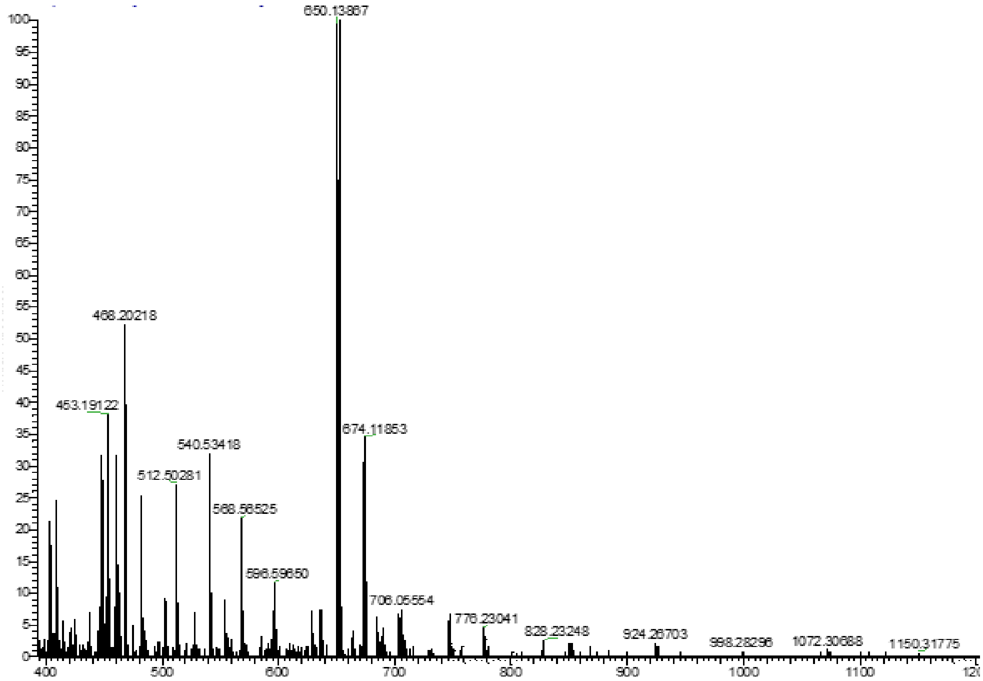

HRMS spectrum of D21

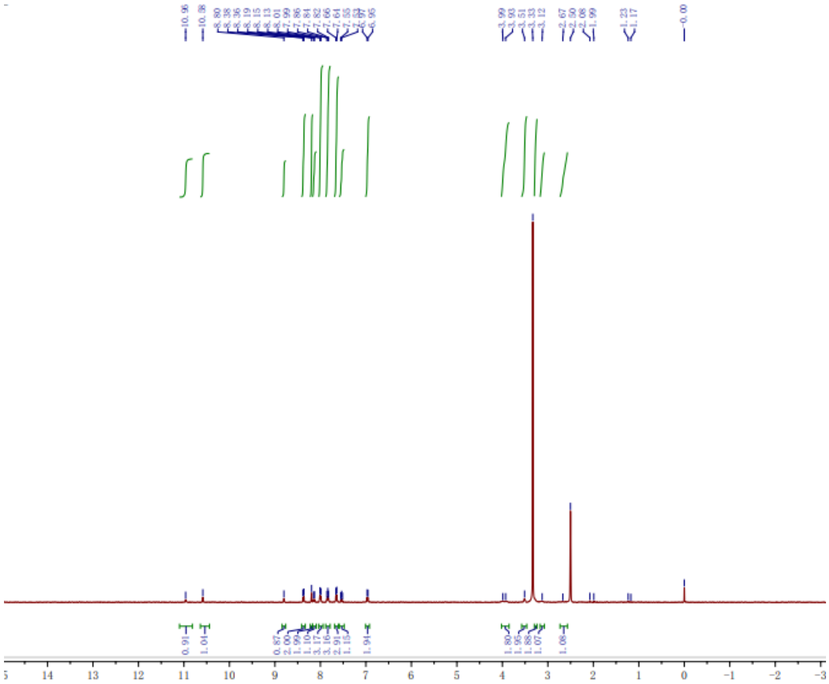

^{1}H-NMR spectrum of D21

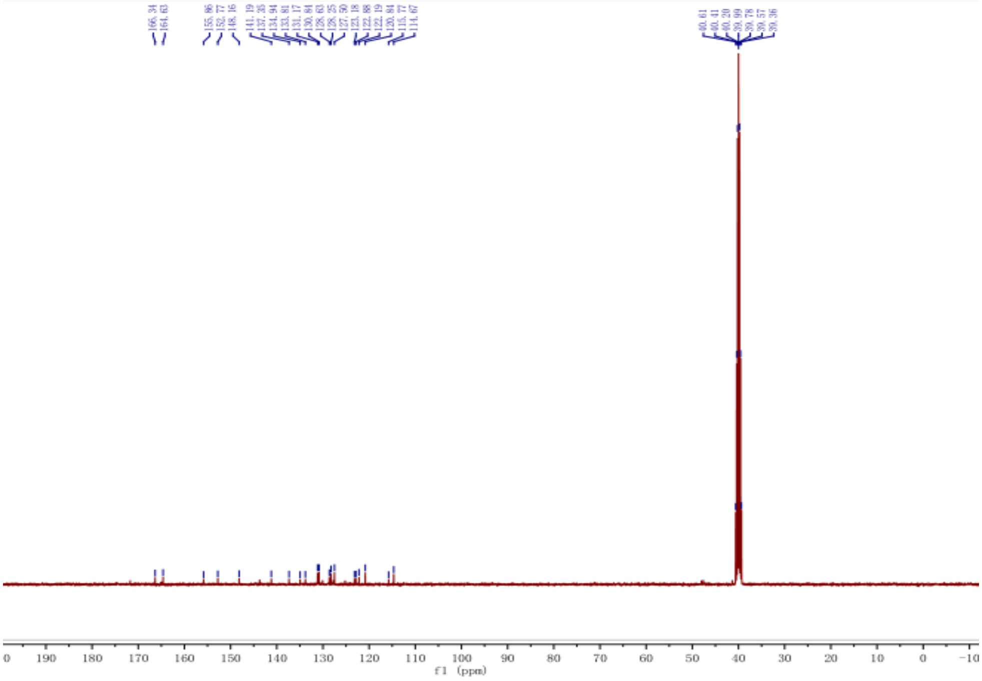

^{13}C-NMR spectrum of D21

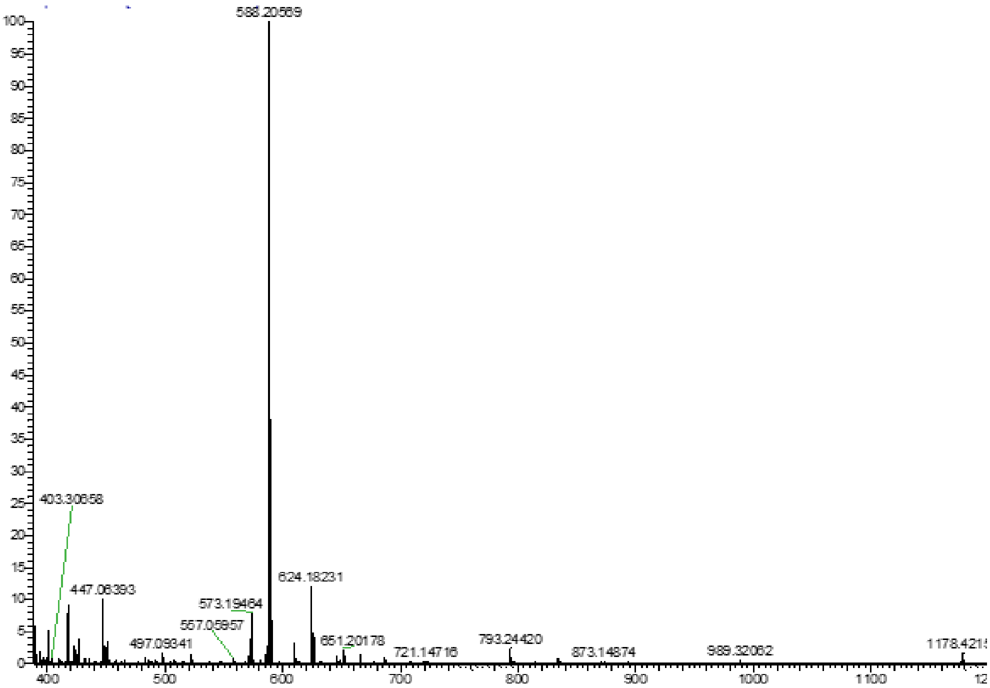

HRMS spectrum of D22

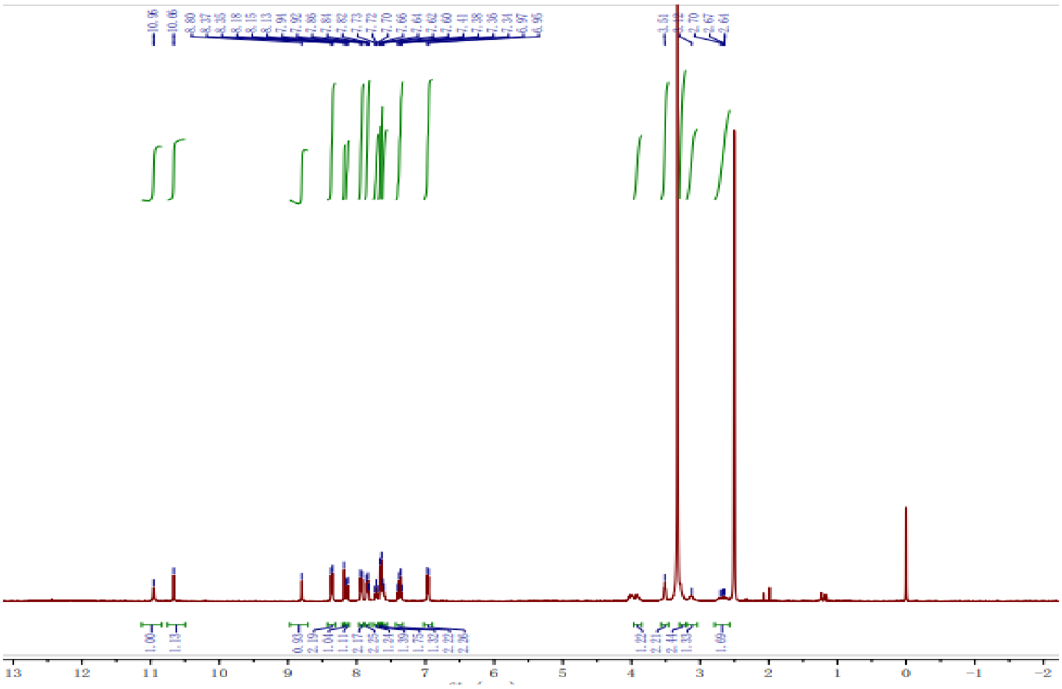

^{1}H-NMR spectrum of D22

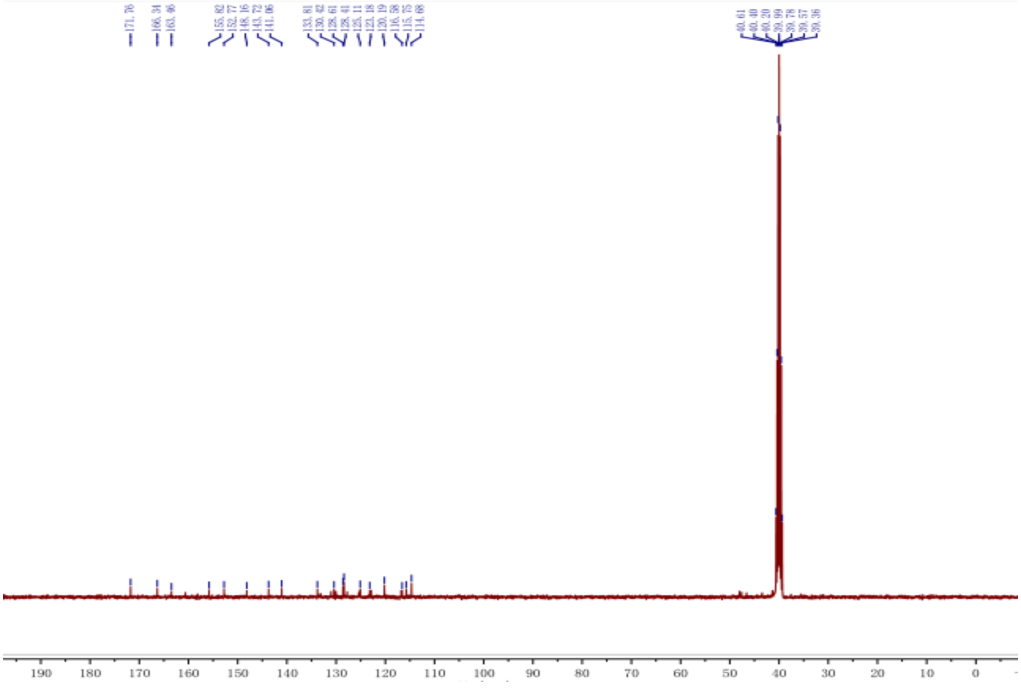

^{13}C-NMR spectrum of D22

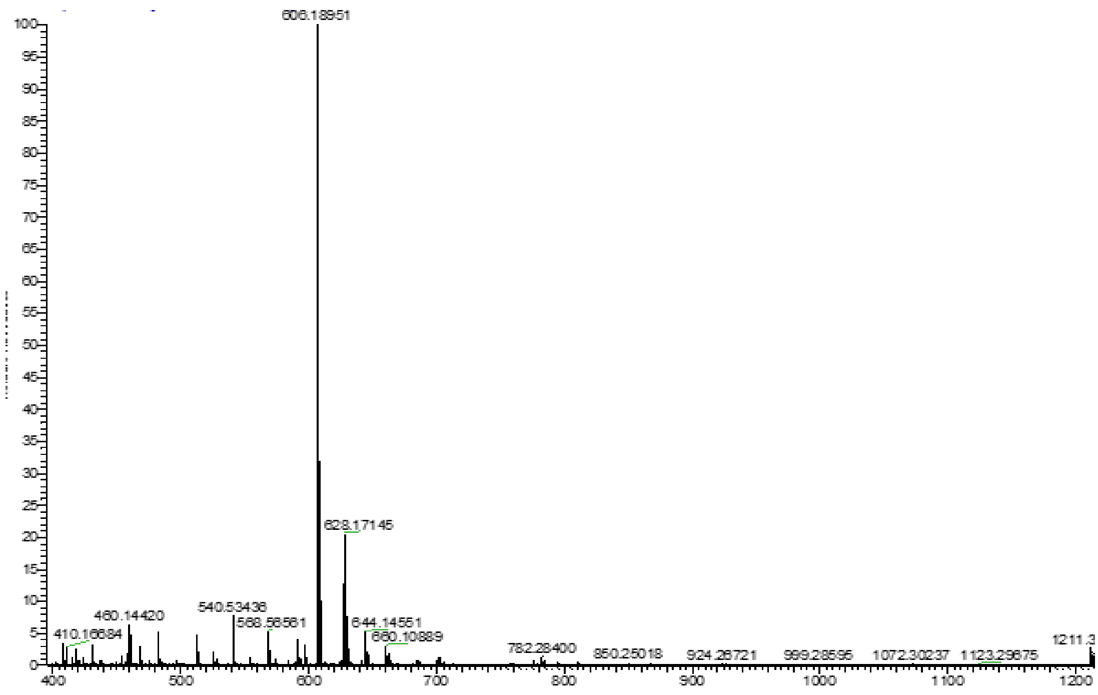

HRMS spectrum of D23

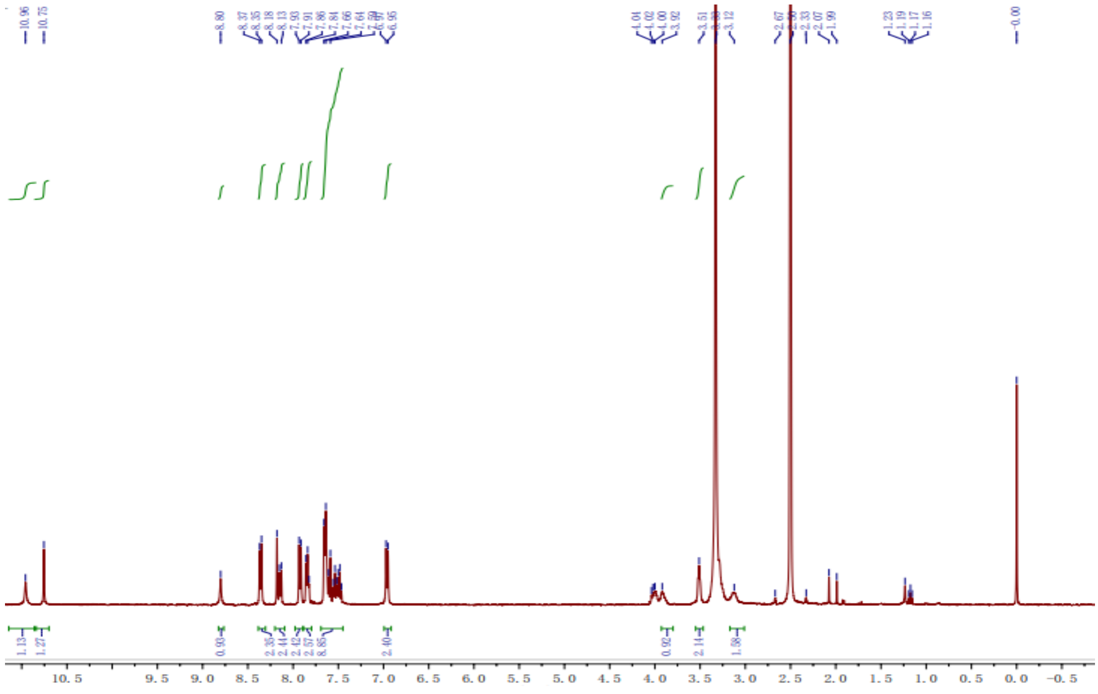

^{1}H-NMR spectrum of D23

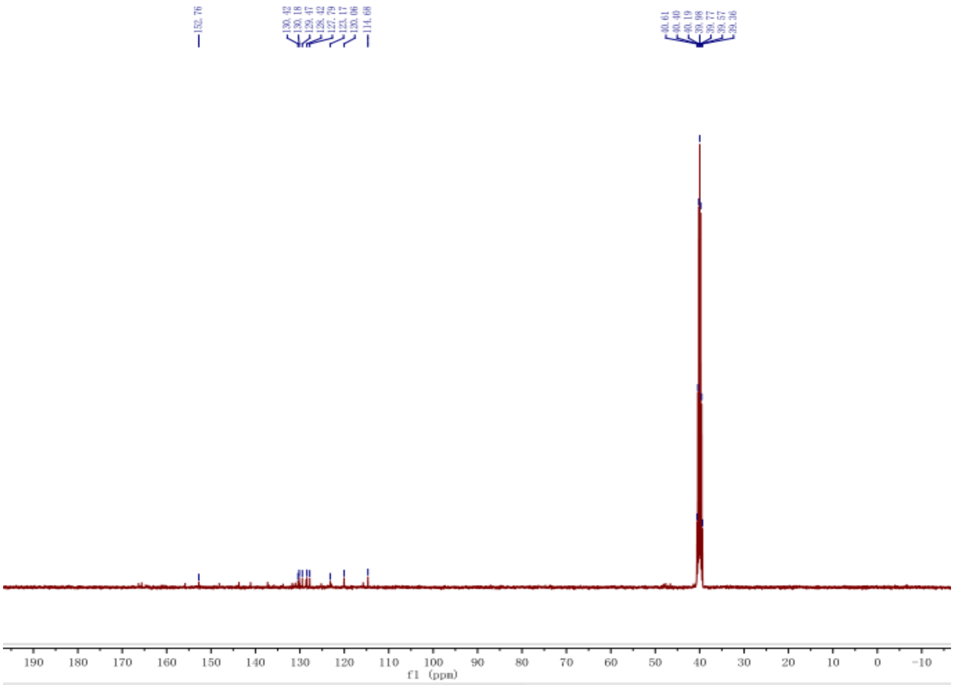

^{13}C-NMR spectrum of D23

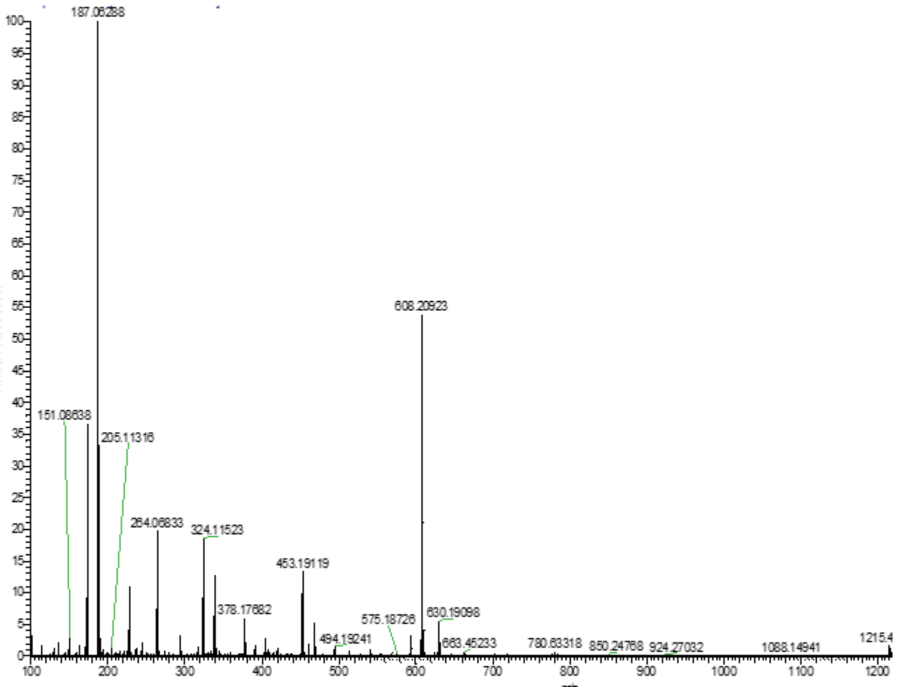

HRMS spectrum of D24

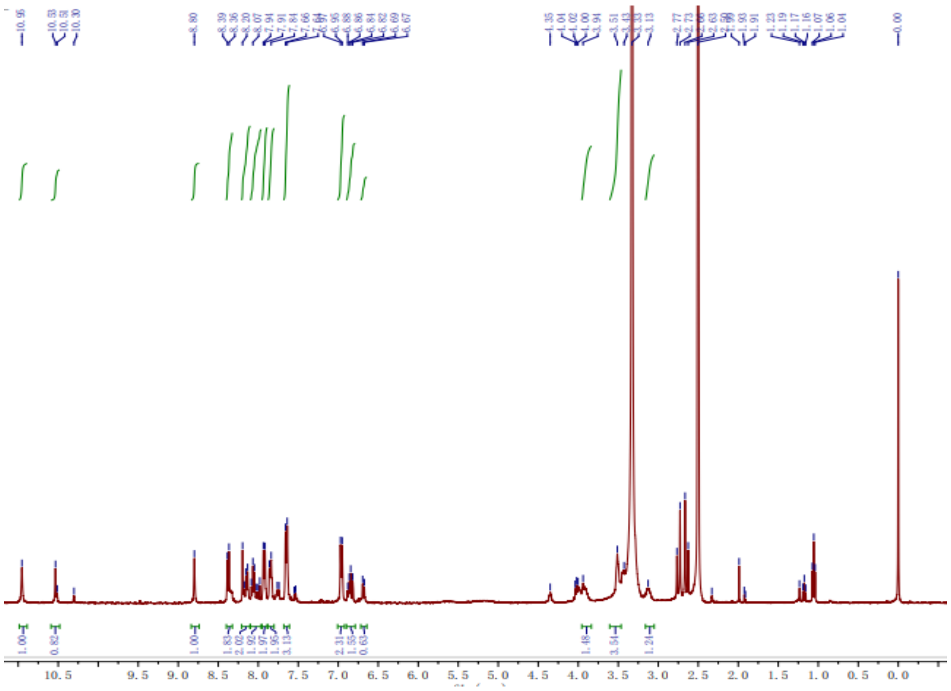

^{1}H-NMR spectrum of D24

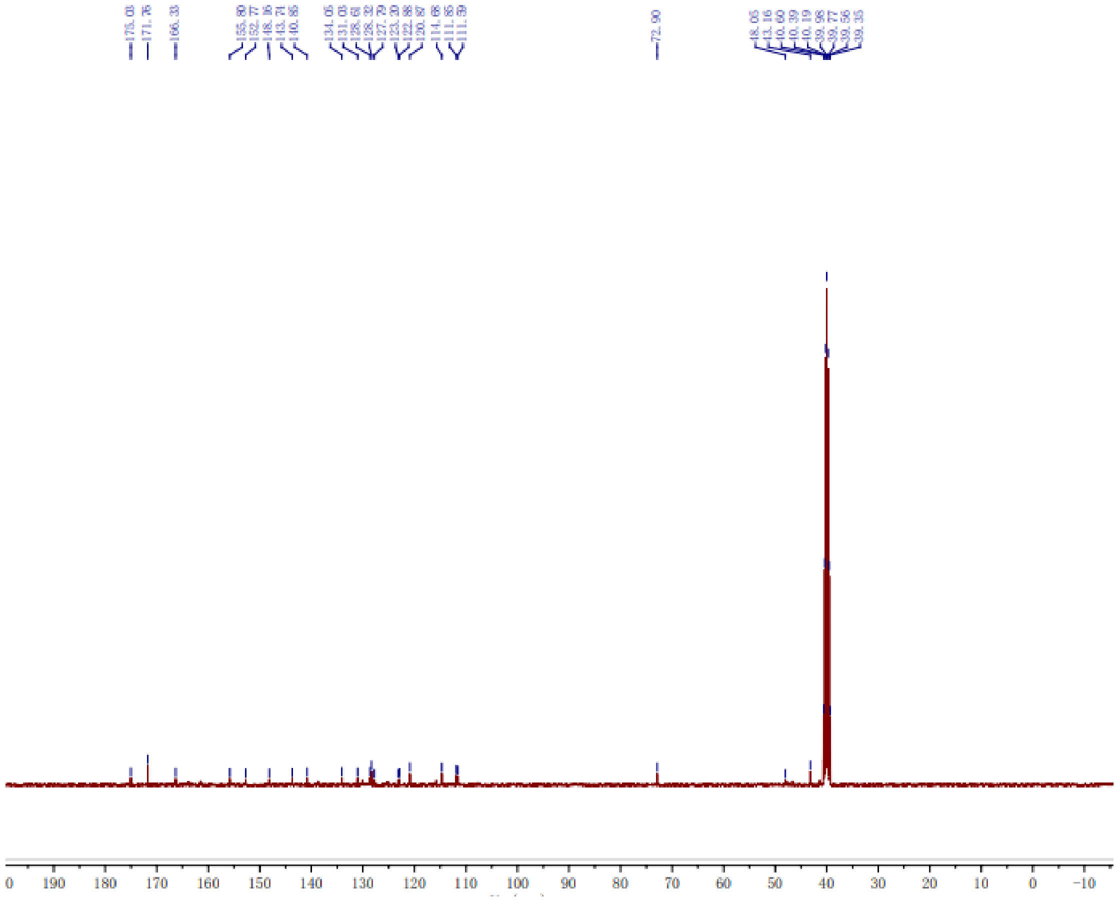

^{13}C-NMR spectrum of D24

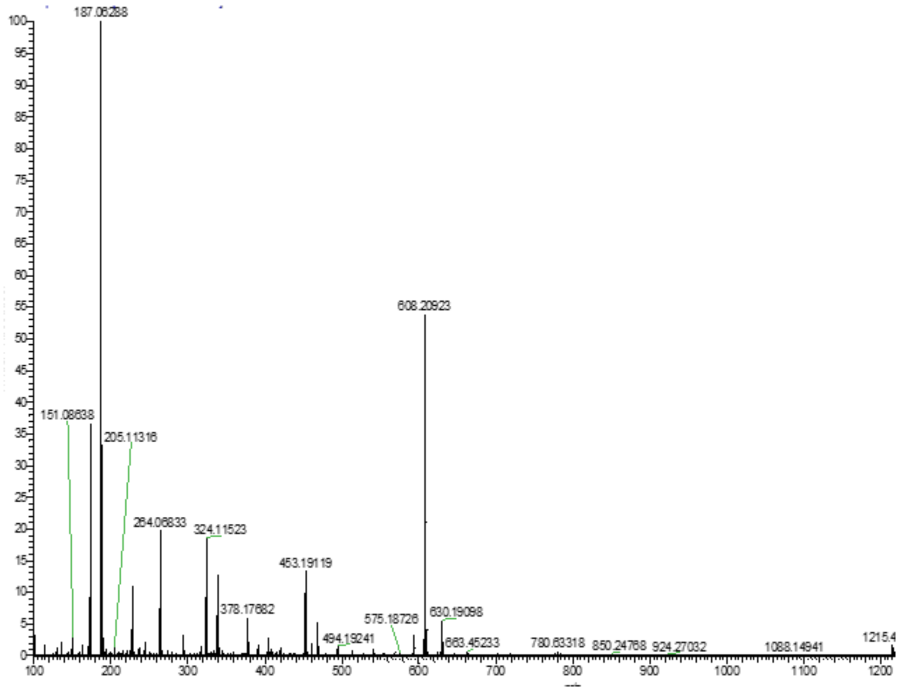

HRMS spectrum of D25

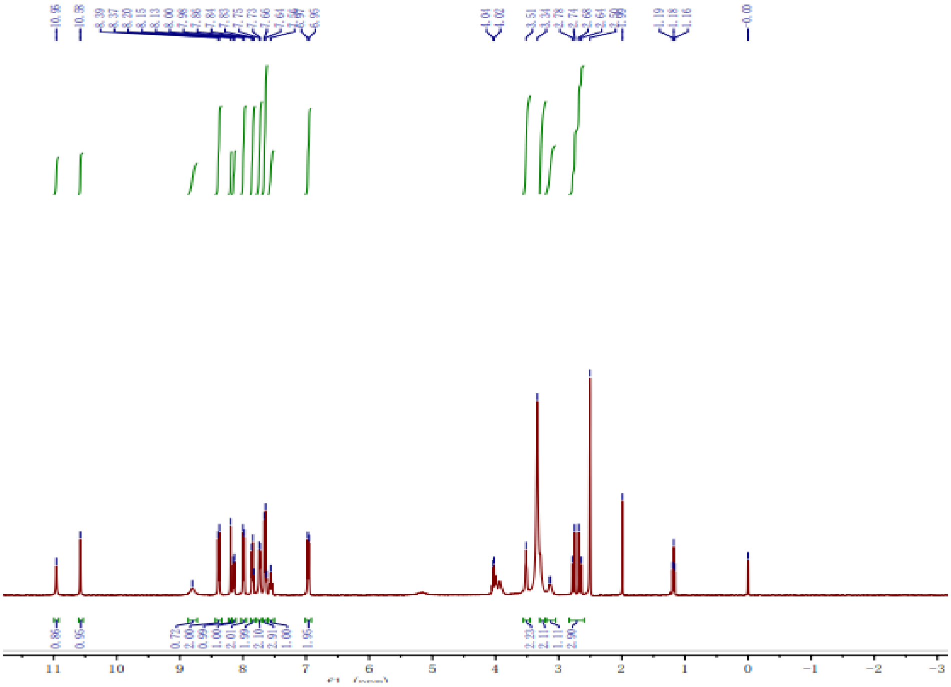

1H-NMR spectrum of D25

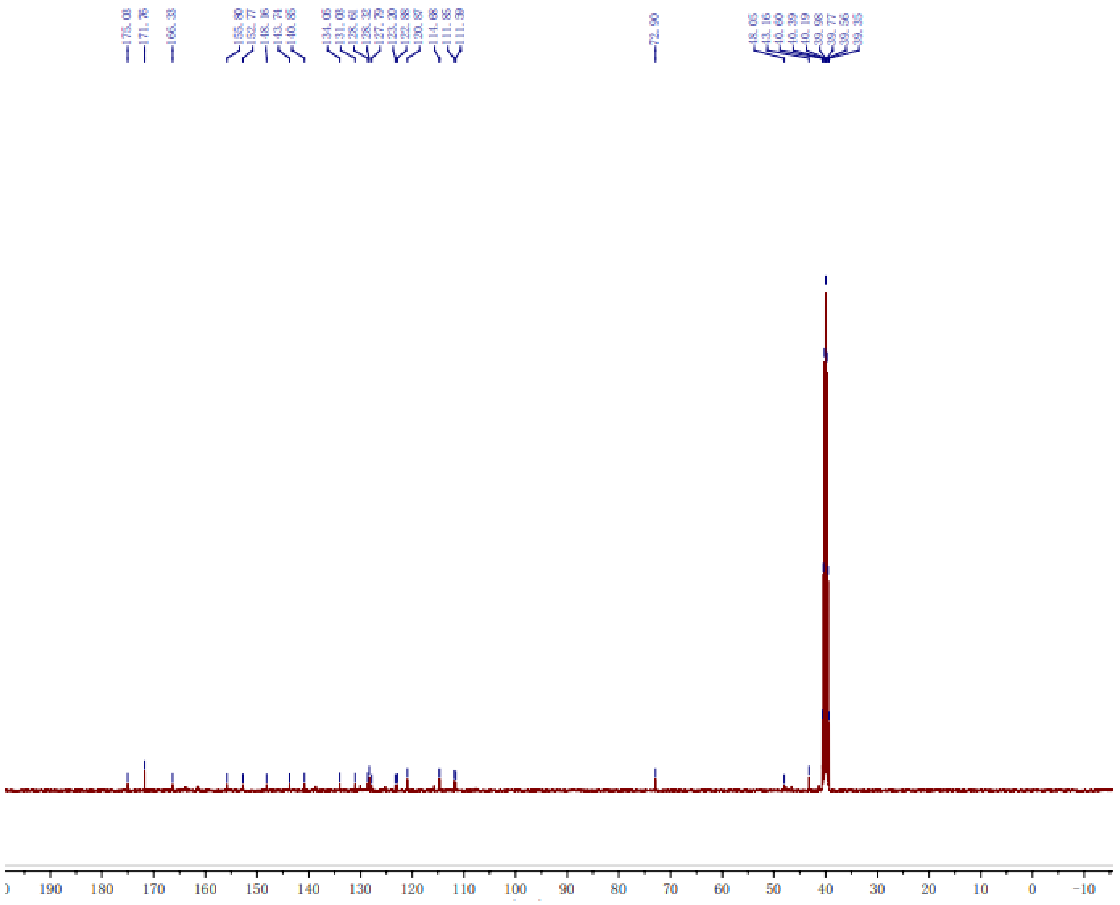

^{13}C-NMR spectrum of D25

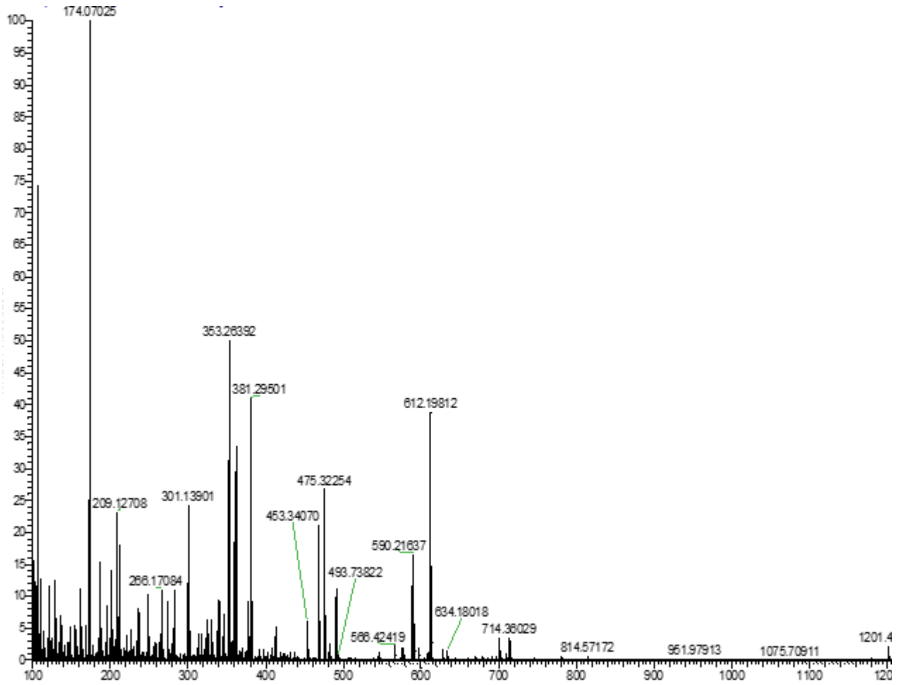

HRMS spectrum of D26

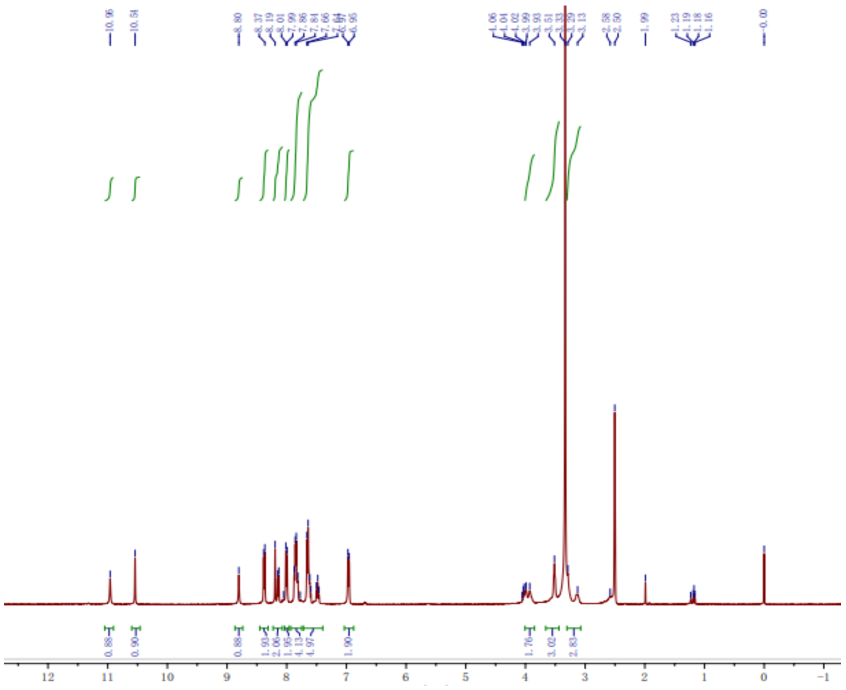

^{1}H-NMR spectrum of D26

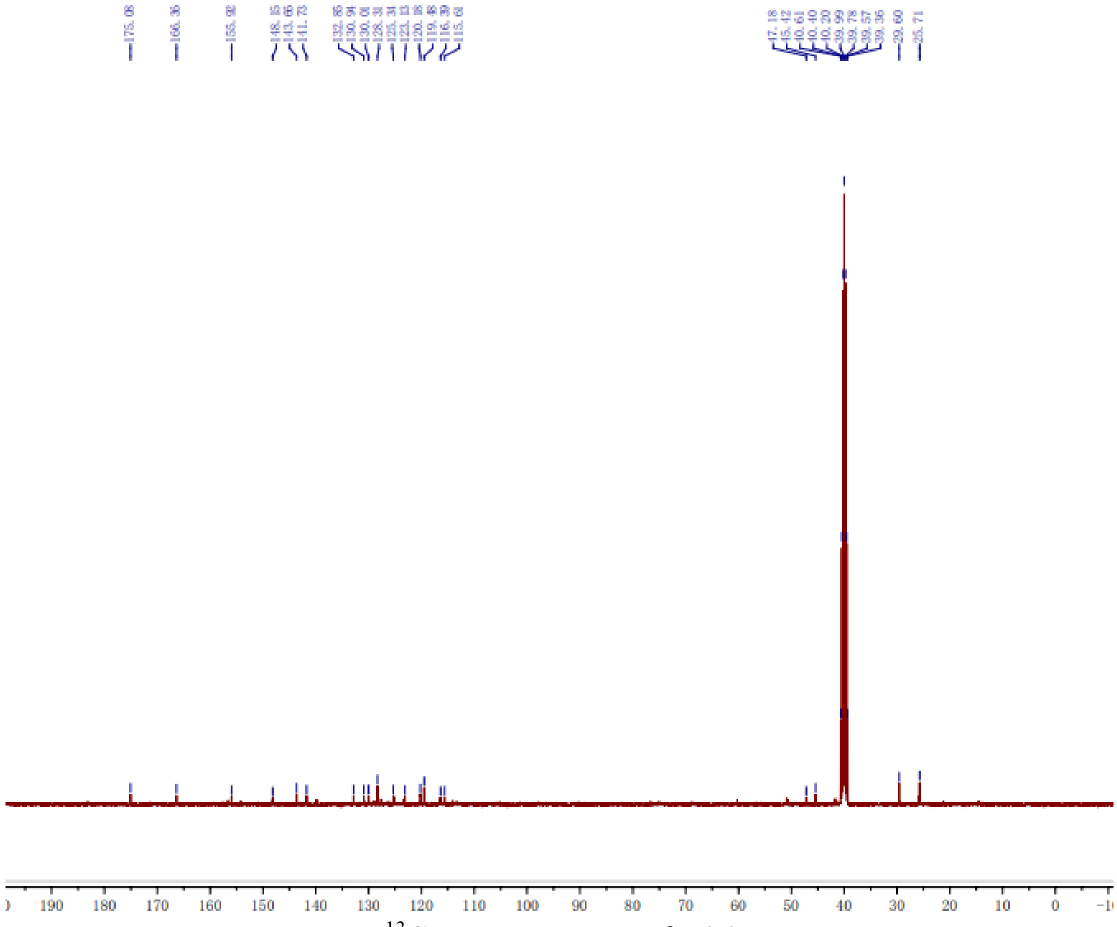

^{13}C-NMR spectrum of D26

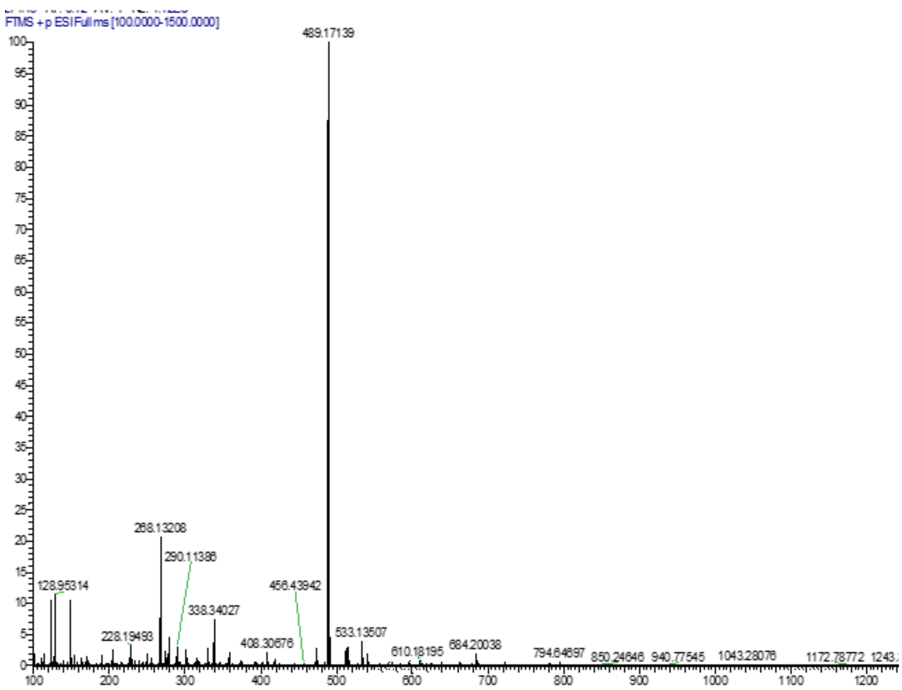

HRMS spectrum of D27

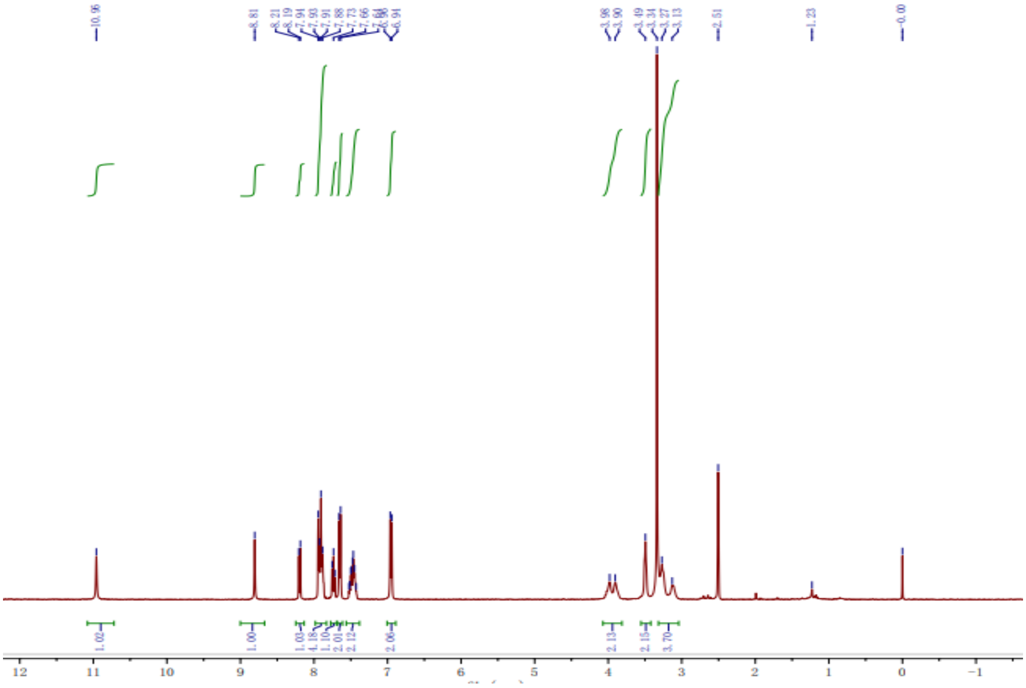

^{1}H-NMR spectrum of D27

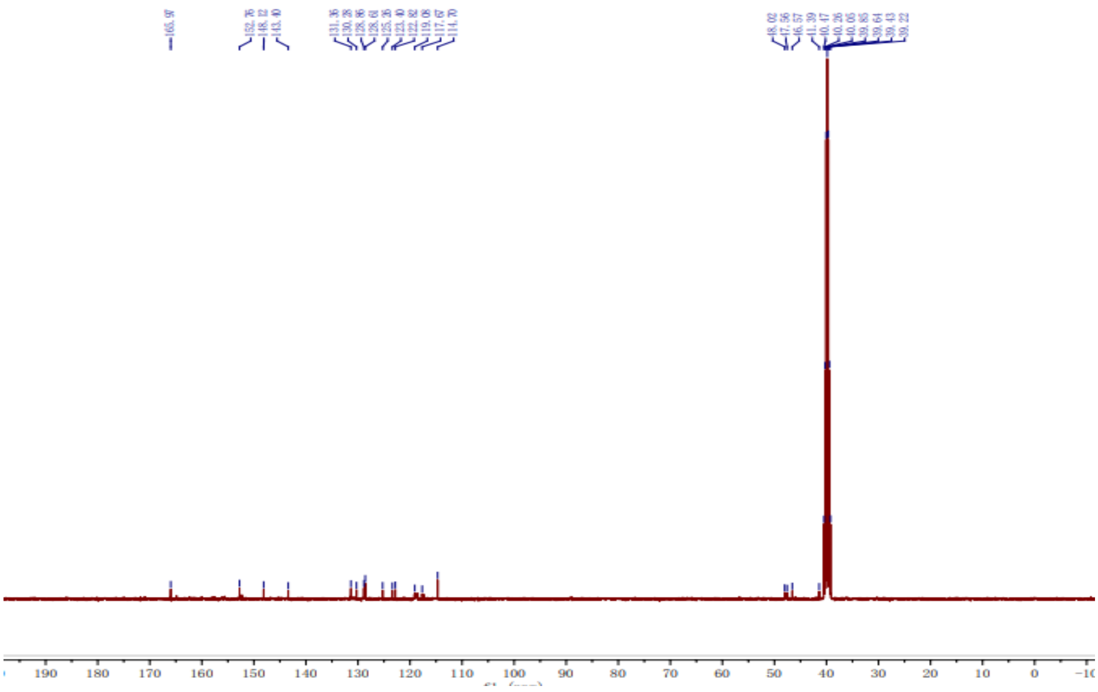

^{13}C-NMR spectrum of D27

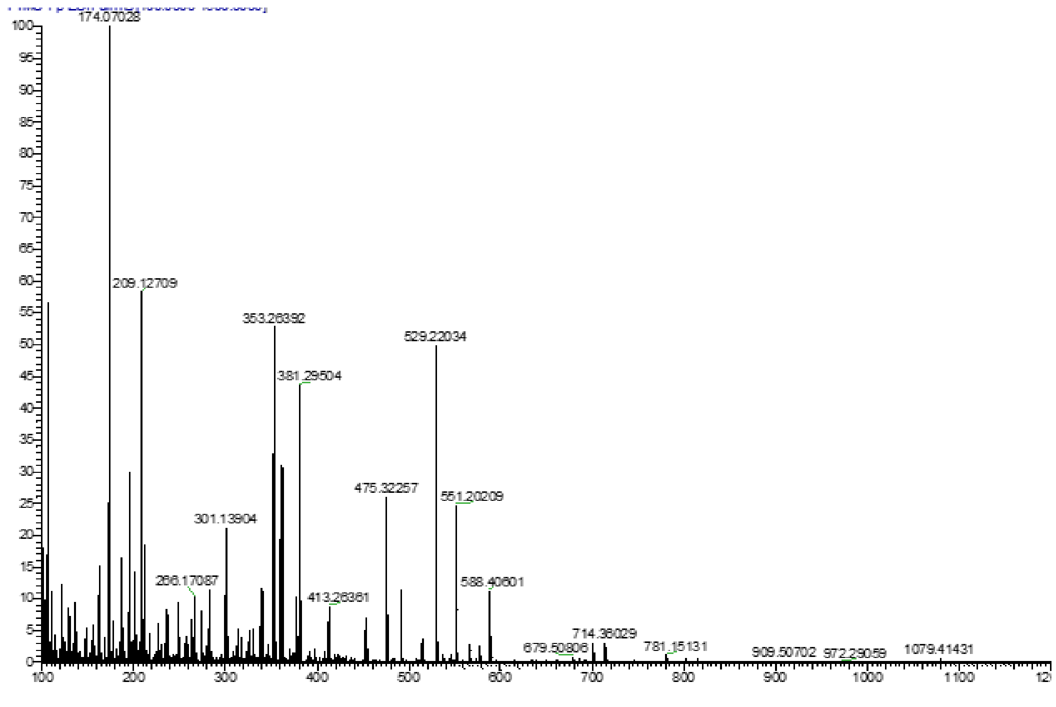

HRMS spectrum of D28

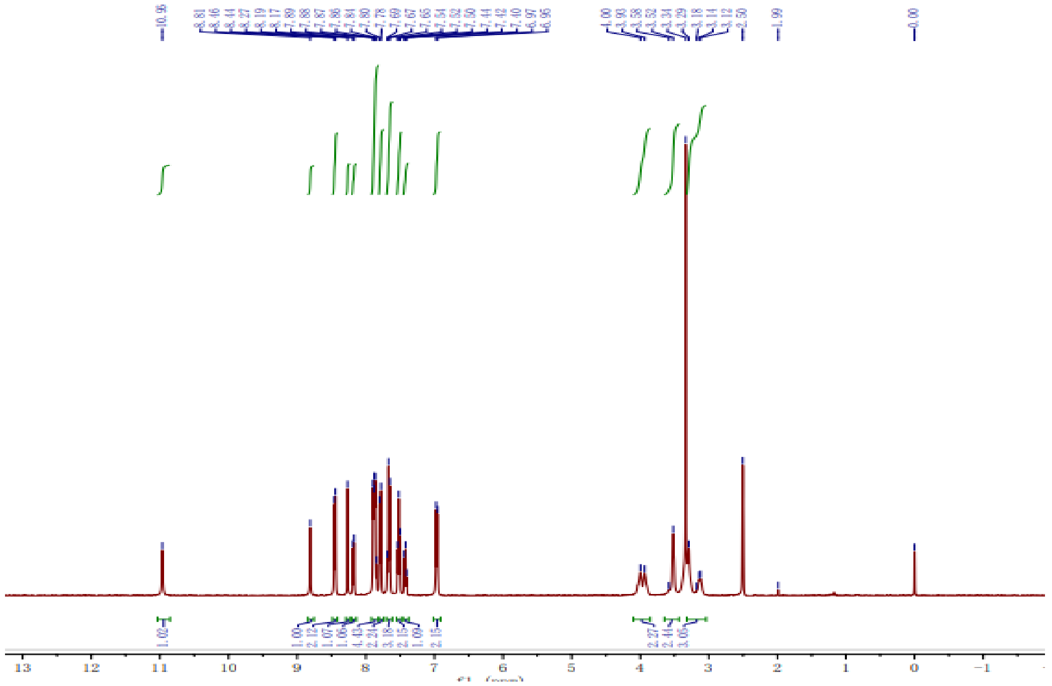

^{1}H-NMR spectrum of D28

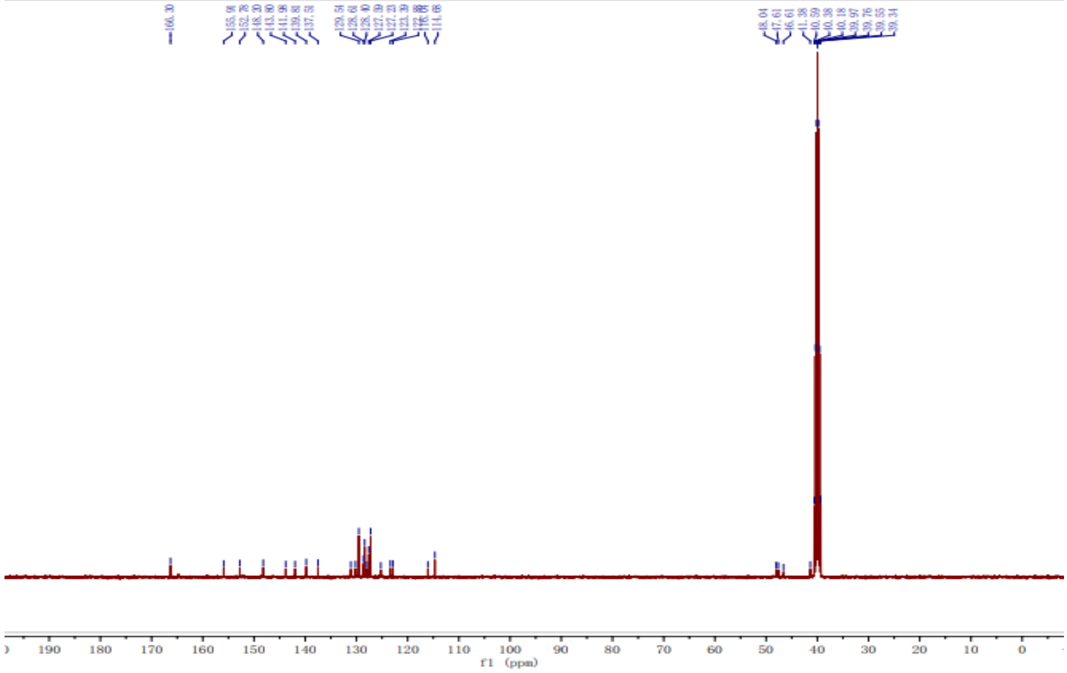

^{13}C-NMR spectrum of D28

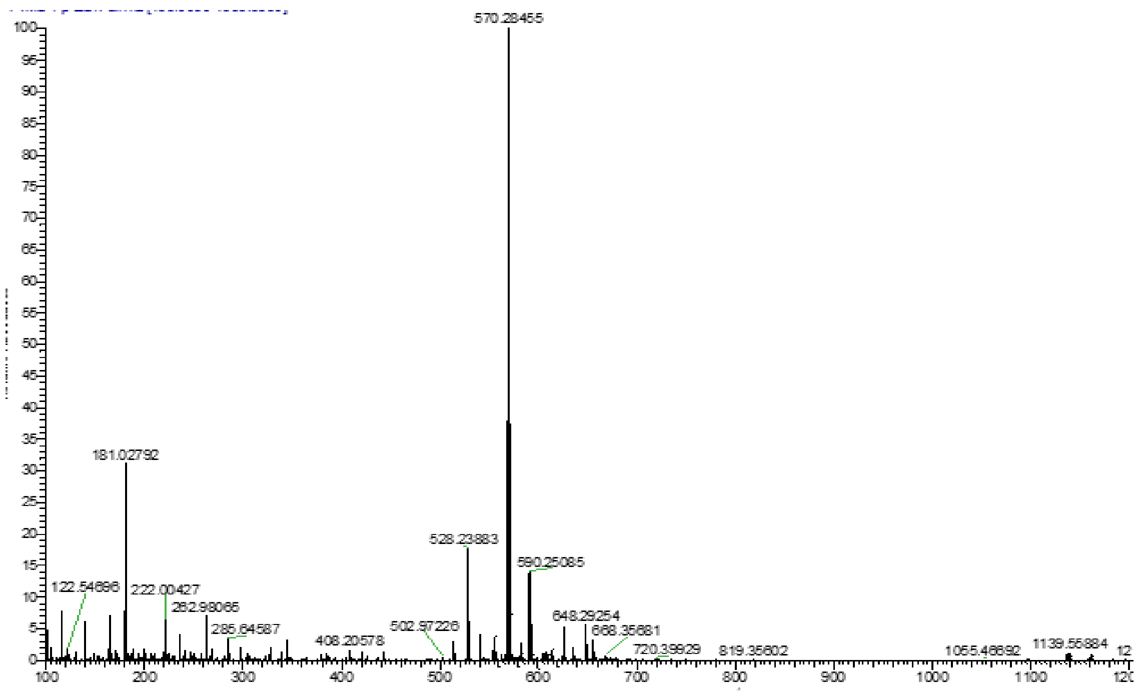

HRMS spectrum of **D29**

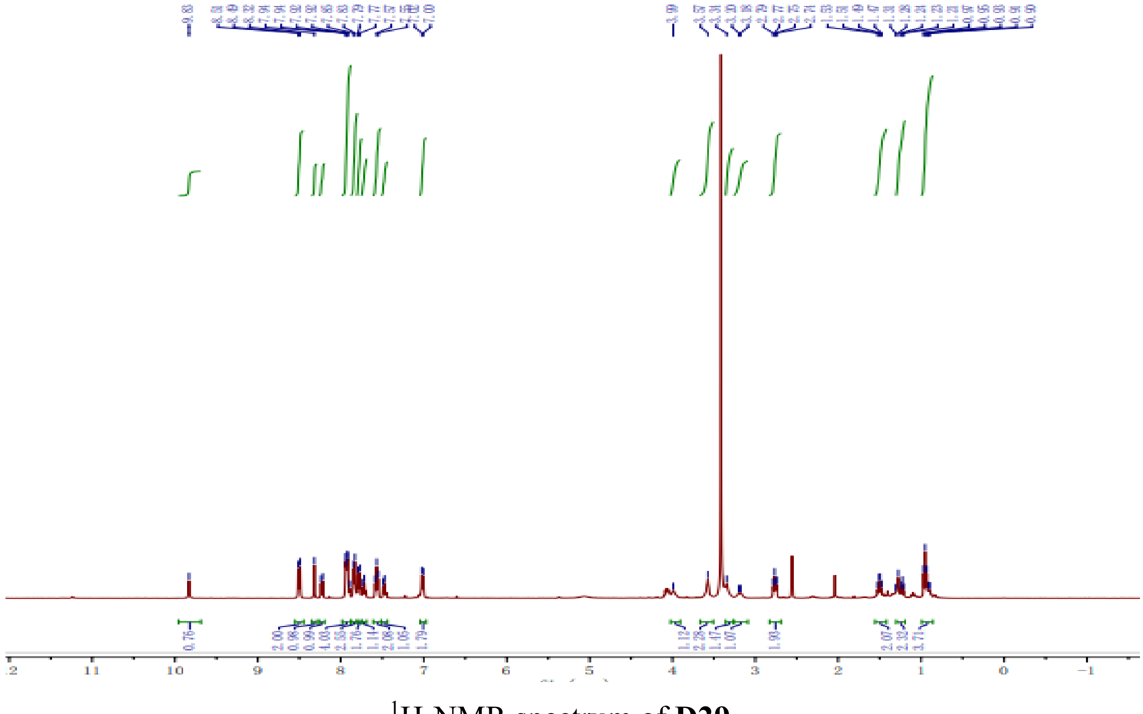

^{1}H-NMR spectrum of **D29**

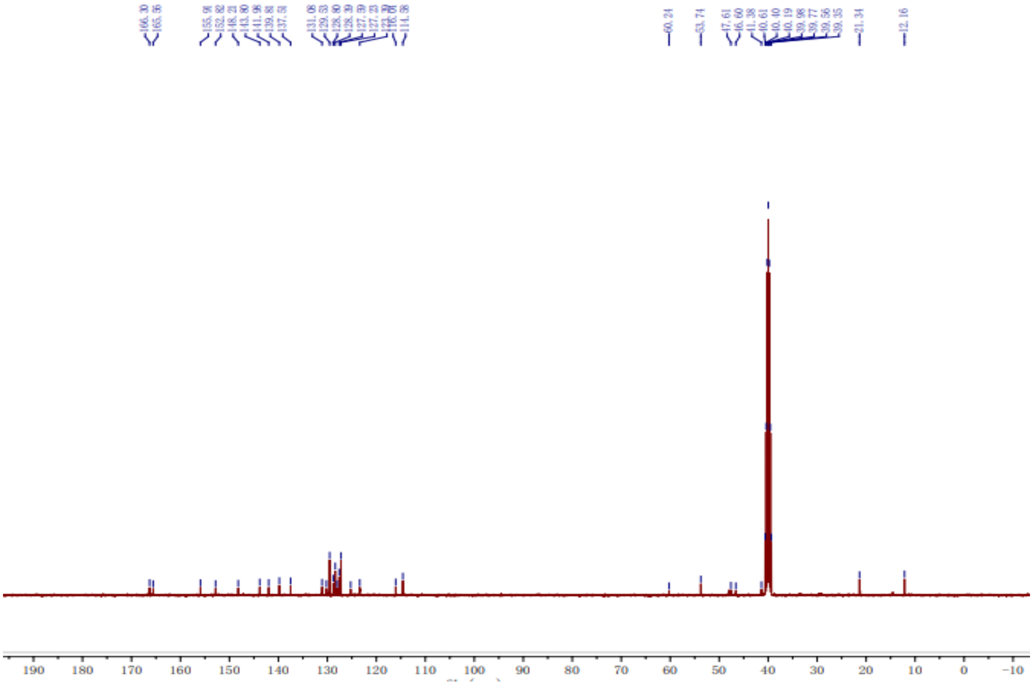

^{13}C-NMR spectrum of D29

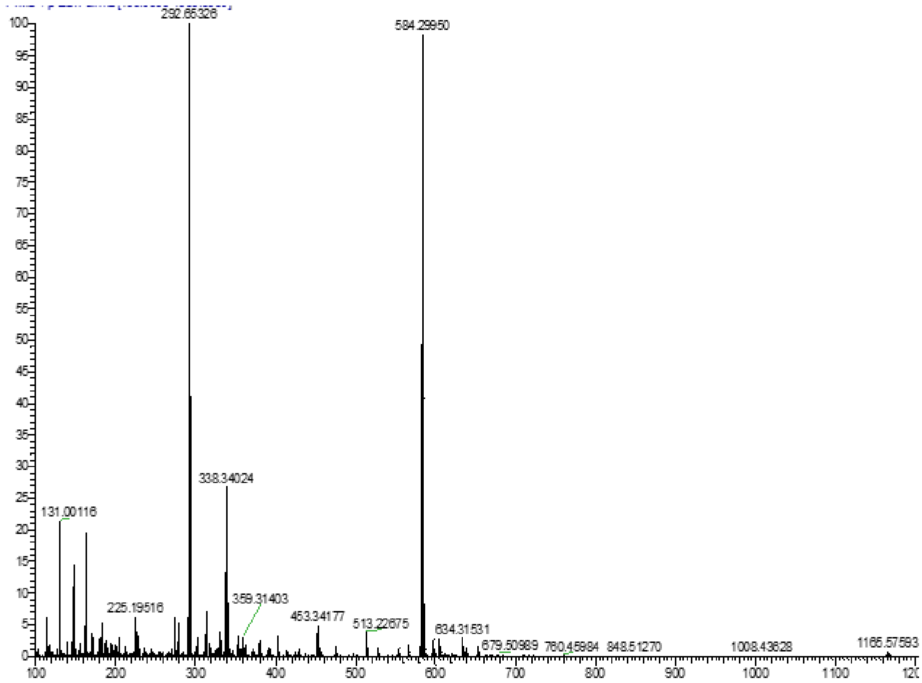

^{1}H-NMR spectrum of **D30**

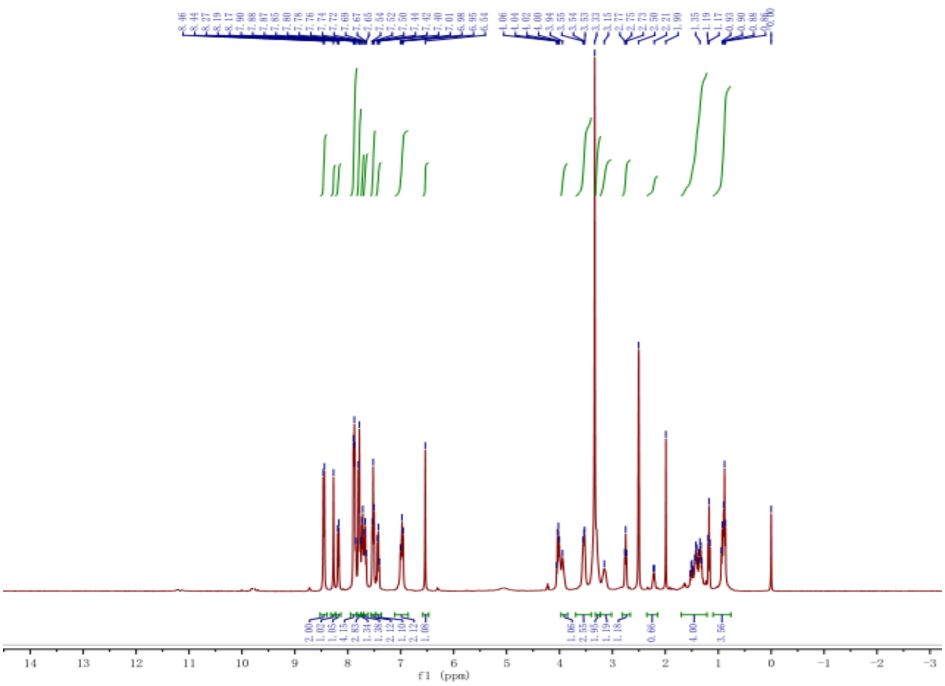

^{1}H-NMR spectrum of **D30**

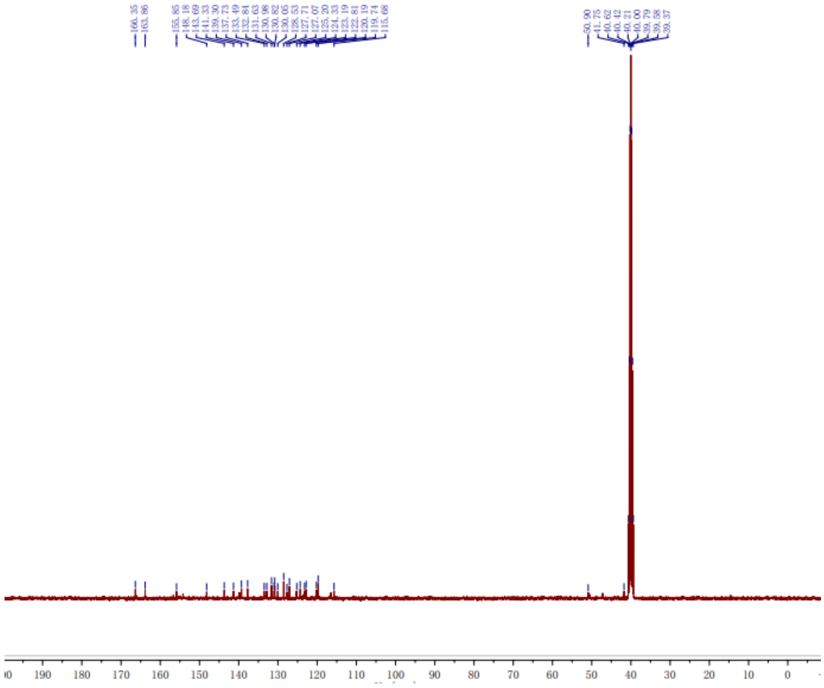

^{13}C-NMR spectrum of D30
